# Responses and adaptations of plants to abiotic stress: transcriptional regulation of secondary metabolic pathways, metabolomics, and nanobiological approaches

**DOI:** 10.3389/fgene.2026.1762633

**Published:** 2026-05-19

**Authors:** Yashdeep Srivastava, Neha Saini, Nishu, Sapna Yadav, Sandhya Tripathi, Akshay Kumar, Neelam Yadav, Monika Saini, Neelam S. Sangwan

**Affiliations:** 1 Department of Biotechnology, Invertis University, Bareilly, Uttar Pradesh, India; 2 Department of Biochemistry, Central University of Haryana, Haryana, India; 3 Department of Biochemistry, ICAR - Indian Institute of Pulses Research, Kanpur, India

**Keywords:** abiotic stress, crop resilience, nanobiology, omics, secondary metabolic pathways, stress signaling, transcription factor

## Abstract

Plants are continuously exposed to diverse abiotic stresses such as drought, salinity, extreme temperatures, and heavy metal toxicity, each of which disrupts cellular homeostasis and reactive oxygen species (ROS) generation and impairs development. To survive under these adverse conditions, plants activate a complex network of transcriptional regulators that remodel primary and secondary metabolic pathways. These regulatory cascades, involving transcription factors such as AP2, WUSCHEL, MYB, bHLH, WRKY, and NAC, orchestrate the biosynthesis of key secondary metabolites, including phenolics, flavonoids, terpenoids, and alkaloids, that function as antioxidants, osmoprotectants, and signaling molecules. Advances in metabolomics have provided deeper insights into stress-induced metabolic reconfigurations, enabling high-resolution profiling of pathway metabolic fluxes and revealing novel metabolites associated with adaptive resilience and tolerance. Alongside engineering abiotic stress resistance, nanobiological approaches have emerged as innovative strategies to modulate and remediate transcriptional responses and secondary metabolite production. This review comprehends current understanding of transcriptional regulation of secondary metabolism under abiotic stress, integrates metabolomics-driven discoveries, and highlights the potential of metabolites and metabolomics-based tools to augment plant adaptive mechanisms. Together, these interconnected perspectives offer a comprehensive framework for developing stress-resilient crops in the era of climate change.

## 1 Introduction

The plants are immobile, so they are continuously exposed to a variety of abiotic stresses such as drought, salinity, extreme temperature, heavy metals, and nutrient deficiencies in their environment. These abiotic stresses severely restrict plant growth, metabolism, and reproduction (Feng et al., 2025). Collectively, abiotic stresses account for the majority of worldwide crop losses in yield (a >50% reduction has been reported on major crops), which are further compounded by climate change-related issues (Feller et al., 2014). Among the abiotic stresses, drought, salinity, heat, and cold stresses are the most deleterious; they modify plant morphology along with physiology and molecular metabolism (Souza et al., 2025; Feng et al., 2025; Feller et al., 2014) (Table 1). Such stresses typically induce a series of interconnected forms of cellular injury resulting from osmotic disturbances, oxidative damage, and metabolic stress (Garcia-Caparros et al., 2021). To cope with such stress, plants activate molecular, cellular, and physiological processes, including stress sensors, signal transduction pathways, and transcriptional regulation of stress-responsive genes (Martin et al., 2021; Qin et al., 2022; Saini et al., 2024).

**TABLE 1:** Primary metabolite regulation in plants under abiotic stress.

Abiotic stress	Metabolite class	Plant	Affected pathway	Metabolite change	References
Drought	Lipids	*Arabidopsis thaliana*; wheat (*Triticum aestivum*)	Phospholipid degradation and jasmonate biosynthesis; galactolipid biosynthesis	Increased phosphatidic acid (PA) and jasmonates for signaling and stomatal regulation; reduced MGDG and DGDG affecting thylakoid membrane stability	Bharath et al. (2021); Wang et al. (2020)
Salinity	Lipids	Soybean (*Glycine max*)	Sphingolipid biosynthesis	Enhanced ceramide and sphingosine levels trigger programmed cell death and salt tolerance	Henschel et al. (2024)
Heat stress	Lipids	Tomato (*Solanum lycopersicum*)	Phospholipid and oxylipin pathways	Accumulation of oxylipins (e.g., hydroperoxyoctadecadienoic acids) for antioxidant protection	Fitoussi et al. (2021)
Cold stress	Lipids	*Arabidopsis thaliana*; barley (*Hordeum vulgare*)	Fatty acid desaturation and lipid remodeling; phosphatidylcholine and galactolipid metabolism	Enhanced polyunsaturated fatty acid (PUFA) levels improve membrane fluidity; increased galactolipid synthesis stabilizes thylakoid membranes	Coniglio et al. (2023); Zhao M. et al. (2024)
Heavy metal stress	Lipids	Cucumber (*Cucumis sativus*)	Lipid peroxidation and oxylipin biosynthesis	Accumulation of malondialdehyde (MDA) and oxylipins for signaling and repair	Gajewska et al. (2024)
Heat stress	Lipids (fatty acids)	Sunflower (*Helianthus annuus*)	Fatty acid metabolism	Altered fatty acid composition maintains membrane stability under high temperature	Rahnama et al. (2024)
Cold stress	Lipids	Soybean (*Glycine max*)	Lipid remodeling and fatty acid metabolism	Altered lipid composition, enhancing membrane fluidity under cold stress	Liu et al. (2024)
Drought	Carbohydrates	Wheat (*Triticum aestivum*)	Glycolysis and sucrose biosynthesis	Increased sucrose, glucose, and fructose for osmotic balance and ROS detoxification	Zurawski et al. (1975)
Salinity	Carbohydrates	Tomato (*Solanum lycopersicum*)	Starch degradation and sucrose biosynthesis	Decreased starch and increased sucrose and glucose for osmotic adjustment	Zhang et al. (2024a)
Heat stress	Carbohydrates	*Arabidopsis thaliana*; soybean (*Glycine max*)	Glycolysis and starch degradation; trehalose biosynthesis	Reduced starch synthesis; increased hexoses and trehalose, protecting cellular structures and mitigating ROS	Korte et al. (2023); Jianing et al. (2022)
Cold stress	Carbohydrates	Potato (*Solanum tuberosum*)	Raffinose family oligosaccharides (RFO) pathway	Accumulation of raffinose, sucrose, and galactinol for cryoprotection and membrane stabilization	Folgado et al. (2014)
Cold stress	Carbohydrates (RFOs)	Pea (*Pisum sativum*)	Raffinose family oligosaccharides pathway	Synthesis of raffinose family oligosaccharides protecting cellular structures under freezing	Elango et al. (2022)
Drought	Carbohydrates (soluble sugars)	*Arabidopsis thaliana*	Glycolysis and sugar metabolism	Accumulation of soluble sugars maintains osmotic balance and protects cellular structures	Yang et al. (2021)
Flooding	Carbohydrates (starch)	Rice (*Oryza sativa*)	Starch degradation	Reduced starch degradation, conserving energy under submergence	Panda et al. (2021)
Drought	Organic acids	Wheat (*Triticum aestivum*); asparagus (*A. officinalis*)	TCA cycle; glycolysis; pyruvate metabolism	Increased malate and citrate support osmotic adjustment, energy production, and metabolic reprogramming	Marček et al. (2019); Zhang et al. (2024b)
Salinity	Organic acids	Tomato (*Solanum lycopersicum*)	GABA shunt	Accumulation of GABA and succinate contributes to osmoprotection and signaling	Guo et al. (2022)
Heat stress	Organic acids	Soybean (*Glycine max*)	Malate valve pathway	Enhanced malate production maintaining redox balance during heat stress	Dinakar et al. (2016)
Cold stress	Organic acids	Potato (*Solanum tuberosum*); *A. thaliana*	Glyoxylate cycle and TCA cycle; shikimate pathway	Accumulation of malate, citrate, shikimate, and α-ketoglutarate supporting cryoprotection and secondary metabolism	Lee et al. (2023); Xu et al. (2020)
Heat stress	Organic acids	Maize (*Zea mays*)	TCA cycle and organic acid metabolism	Increased organic acid synthesis maintains metabolic flux and reduces heat damage	Qu et al. (2018)
Salinity	Amino acids (proline)	Wheat (*Triticum aestivum*)	Proline biosynthesis	Proline accumulation acts as an osmoprotectant and an ROS scavenger	Renzetti et al. (2024)
Salinity	Polyamines	Barley (*Hordeum vulgare*)	Polyamine biosynthesis	Enhanced polyamine synthesis stabilizes membranes and mitigates ionic toxicity	Pottosin et al. (2014)
UV radiation	Antioxidants (ascorbic acid)	Tomato (*Solanum lycopersicum*)	Ascorbate–glutathione cycle	Increased ascorbic acid reduces UV-induced oxidative stress	Mishra et al. (2024); Mishra et al. (2017)
Drought	Osmoprotectants (glycine betaine)	Spinach (*Spinacia oleracea*)	Glycine betaine metabolism	Accumulation of glycine betaine maintains water potential and protects macromolecules	Yang et al. (2021)

Plants can dynamically reprogram their primary and secondary metabolism in response to abiotic stress such as drought, salinity, or extreme temperatures (Ghasemi et al., 2023; Sangwan et al., 1994; Mishra et al., 2024). This reprogramming is a vital survival mechanism that involves redirecting resources toward the production of specific protective metabolites. Phenolics, terpenoids, and alkaloids accumulate in response to stresses and act as antioxidant agents with the ability to scavenge free radicals (Sangwan et al., 1994). They also serve as osmoprotectants, as well as protectional for UV radiations and contributors to cell wall reinforcement. This metabolic adjustment increases plant tolerance, enabling acclimatization and survival under stress conditions (Mishra et al., 2024). Finally, this mechanism ensures not only survival by several physiological modifications but also can enhance the medicinal and nutritional quality of the plants (Mishra et al., 2024). Understanding these mechanisms is essential for molecular breeding and biotechnological approaches aimed at producing stress-resilient crop varieties, thereby ensuring sustainable agricultural yields under the changing environmental conditions (Feng et al., 2025).

**FIGURE 1:**
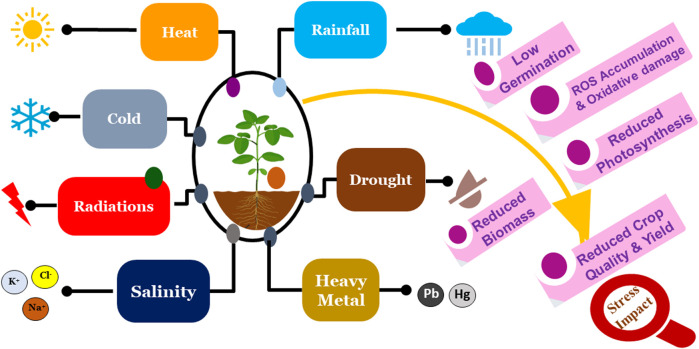
Effects of abiotic stress on plant growth and productivity.

**FIGURE 2:**
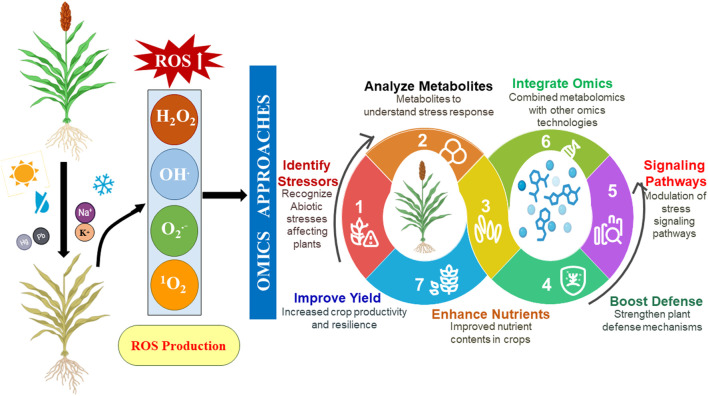
Omics-driven functional understanding of abiotic stress-induced ROS signaling for improving plant resilience.

Crop productivity is affected from seed germination to the reproductive stage through abiotic stresses like higher salinity, drought, temperature variations, and heavy metals (Figure 1) (Table 1). Heavy metals are extremely harmful and hazardous abiotic stressors for plants. Non-biodegradable metallic elements with a very high density are known as heavy metals. Trace amounts of many heavy metals, like nickel (Ni), arsenic (As), cadmium (Cd), zinc (Zn), lead (Pb), copper (Cu), and cobalt (Co), are important for plant growth and development (Mishra et al., 2017; Mishra et al., 2024). However, excessive accumulation of these heavy metals within plants causes harmful effects. Heavy metal toxicity elevates the oxidative stress in plants by decreasing the antioxidant enzyme activity, which negatively affects plant development. Another harmful environmental factor for plant growth is high temperature. Plants have regulatory systems to protect them from extreme heat. Elevated environmental temperatures cause denaturation and aggregation of proteins, membrane-damaging lipid peroxidation, enzyme inactivation, protein synthesis suppression, and imbalances between the antioxidant system and reactive oxygen species (ROS) (Saini et al., 2024). Cellular homeostasis is also disturbed by drought stress, which increases ROS generation and lowers antioxidant scavenging system activity. In response to ROS, plants modify their morphology, physiology, biochemistry, and molecular systems to protect themselves (Figure 2; Saini et al., 2024). Salt stress, which results from factors like soil salinity, saline irrigation, and poor soil management, has a significant impact on agricultural production worldwide. It disturbs the ion balance, causes osmotic stress, and damages plants oxidatively, which limits growth, productivity, and reduces crop quality (Saini et al., 2024). These abiotic factors disrupt the balance of agricultural ecosystems, significantly influencing crop productivity, quality, and sustainability. Increasing temperatures are changing the duration of growing seasons, thereby affecting crop yields and productivity, while changes in rainfall season lead to droughts or floods that disrupt soil moisture and nutrient availability (Lesk et al., 2022). Increased soil salinity, driven by natural processes and human activities, has further degraded agricultural land, while severe weather events like heatwaves, storms, and cyclones cause extensive harm to crops (Kumar et al., 2023). As climatic conditions become more unpredictable, these stress factors threaten the stability of agricultural methods and our ability to sustain a rising global population (Shahzad et al., 2021).

Agricultural practices are greatly sensitive to abiotic stress and face a high risk as these stresses intensify due to changing climatic conditions. This necessiate to develop plants which should be more potent, to grow more food, and to keep a safe food supply as the climate changes. It is crucial to recognize how abiotic stress impacts agricultural systems mainly through abiotic stressors to develop sustainable farming practices and improve crop resilience against stress-related challenges.

Plant stress responses have been the subject of extensive research. Most existing reviews focus on transcriptional regulation, metabolomics, or stress physiology separately. This approach creates a limited view of how plants achieve stress tolerance. In this work, we provide a complex synthesis of existing knowledge that highlights metabolomics as a key framework connecting stress perception, acclimation, and crop resilience (Wang H. et al., 2022). Environmental stress is interpreted as a disturbance of metabolic homeostasis. The tolerance to stress is enacted through well-organized metabolic reprogramming and signaling via metabolites. A significant breakthrough of this review article is the mechanism-based grouping of stress-responsive metabolites into the categories of protective compounds, stress-induced by-products, and signaling molecules, thus making a time-resolved view of acclimatization possible. The present review, through its comprehensive integration of metabolomics with transcription factor networks, redox and hormonal signaling, plant–microbe interactions, and novel nanobiological techniques, offers a systems-level and translational viewpoint that immediately supports predictive modeling and metabolomics-assisted breeding for climate-resilient crops.

## 2 Signaling pathways affected under stress conditions

A number of genes and signaling pathways are affected under stress conditions in plants. Abscisic acid (ABA) plays a significant role in regulating the closing of stomata and gene expression during drought and salinity stress. Stress-responsive gene expression is caused by the activation of ABRE-binding factor (ABF) transcription factors and the mediation of ABA perception by the PYR/PYL/RCAR–PP2C–SnRK2 core signaling module (Dong et al., 2022). In *Arabidopsis thaliana*, the SnRK2 gene plays an important role in seed germination, seedling growth, and increasing stress tolerance (Amjad et al., 2024). Similarly, the salt overly sensitive (SOS) pathway helps to balance ion homeostasis in salinity stress. SOS3, a calcium-binding protein, detects salt stress and triggers SOS2, which phosphorylates the SOS1 Na^+^/H^+^ antiporter, thereby decreasing the buildup of Na^+^ ions in cells (Hendricks, 2021). In *A. thaliana*, the SOS pathway plays an essential role in salt tolerance. Similarly, the maize SOS3/SOS2 complex phosphorylates, activating Na^+^-transporting function. Loss-of-function mutants exhibit phenotypes that are hypersensitive to salt (Zhou et al., 2022). In heavy metal stress conditions, plants use phytochelatins (PCs) and metallothioneins (MTs). They chelate and sequester metals such as Cd^2+^ and Pb^2+^ into vacuoles through ABC transporters (Leopold et al., 1999). Heat shock proteins (HSPs), which act as molecular chaperones to stop the denaturation and aggregation of proteins, are activated by heat stress-induced heat shock transcription factors (HSFs). The PIF4 protein, which increased stress tolerance by binding with the HSAF2 promoter and triggering the heat stress response to increase tolerance under heat stress, was found to accumulate in *Arabidopsis* and wheat (Yang et al., 2022). In *Aquilegia vulgaris*, under salt stress, the metabolism of starch and sucrose, as well as the signal transduction of plant hormones, were significantly improved (Chen L. et al., 2023). ROS behave as both stressors and signaling molecules. Oxidative stress triggers the activation of the MAP kinase (MAPK) cascade (MAPK3/MAPK6), which controls genes that counter stress and antioxidants like superoxide dismutase (SOD) and ascorbate peroxidase (APX) (Figure 2). Under stress conditions, plant growth is adversely affected due to the inhibition of cell cycles. Stress causes activation of the signaling cascade that results in the S-phase’s prolonged and delayed entry into mitosis. In maize, downregulation of CDKA, CYCA, CDKB, and CYCB was found under drought and salinity stress, leading to a low cell proliferation rate (Kamal et al., 2021). Similarly, it was found that CDKG-2s might be the primary regulators in cotton (*Gossypium* spp.), while the CDKF4 gene was upregulated in *Arabidopsis* under salinity and drought stress (Magwanga et al., 2018). Metabolomics can be used to analyze the metabolites in biological systems affected by abiotic stress (Tinte et al., 2021), providing a valuable tool to understand the plant’s adaptation and responses against stress conditions, helping to expose the metabolic pathway network that influences plant growth and development. This helps identify key biomarkers and metabolites crucial for stress-resilient crops. This review aims to give an overview of how various abiotic stresses impact crop yield, productivity, and agricultural sustainability. It covers the impact of various types of abiotic stress on metabolites, pathways, regulation, and nanobiotechnological management. The review further emphasizes that a deeper understanding of stress-responsive metabolites can improve crop resilience, optimize resource use, and encourage sustainable farming practices, thereby providing a strategy toward global crop systems during this era of significant environmental change.

## 3 Metabolomics under abiotic stress

**FIGURE 3:**
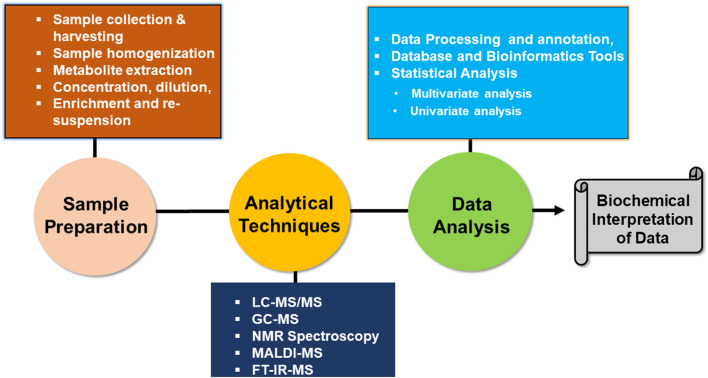
Schematic workflow representing the major steps involved in metabolomics studies, including sample preparation, metabolite extraction, analytical platforms, data processing, and biological interpretation.

In biological systems, metabolomics provides a comprehensive analysis of all metabolites under particular conditions (Klassen et al., 2017). This includes primary and secondary metabolites, which are important for energy production, cell signaling, stress responses, and various metabolic pathways (Saini et al., 2024; Tripathi et al., 2016). Metabolomics also helps to advance precision agriculture practices, promoting sustainable farming and boosting the plant breeding process (Figure 3). This makes it sensitive to stress conditions, disease processes, and other physiological conditions (Satrio et al., 2024). Metabolomics enhances crop quality and yield, and the combination of metabolomics with other “omics” techniques like proteomics, transcriptomics, and genomics helps us understand entire plant systems and their response against stress conditions (Carrera et al., 2021; Yang et al., 2024a). Its aim is to examine qualitative and quantitative forms of metabolites, as well as the metabolic state of samples. This approach provides a unique insight into the biochemical pathways that might not be identified through other molecular biology techniques (Tyagi et al., 2022).

### 3.1 Role of metabolomics in stress biology and crop resilience

Metabolomics plays a pivotal role in stress biology as it expresses the functional biochemical state of plants in response to environmental stress. This directly links stress perception to physiological and adaptive outcomes (Anzano et al., 2022). Environmental stresses alter metabolic homeostasis, which triggers a cascade of signaling events. Metabolic reprogramming is aimed at achieving a new equilibrium through acclimation in stress conditions (Ghatak et al., 2018). As metabolites are the final products of gene expression and protein activity, metabolomic profiling offers the closest and most comprehensive method of understanding stress responses (Singh et al., 2020).

Stress resistance in plants is essentially a metabolic trait that is governed by the complex dynamics of primary and secondary metabolites. Metabolomics contributes to the identification and quantification of molecules participating in different types of stress. These molecules, viz., sugars, amino acids, organic acids, polyols, phenolics, terpenoids, alkaloids, and phytohormones, are involved in redox homeostasis, osmotic adjustment, membrane stabilization, detoxification, and signaling. They function together as osmoprotectants, antioxidants, metal chelators, and signal molecules that affect how plants develop and survive under stress (Khan et al., 2017).

An important role of metabolomics in relation to crop resilience is its ability to detect specific metabolic fingerprints for stress responses (Głuchowska et al., 2025). This function enables the distinction of stress-tolerant crop varieties from stress-sensitive varieties. These metabolic fingerprints serve as ideal biomarkers for crop resistance to stress. They can be exploited for the identification and early detection of stress. In major food crops such as cereals, metabolomic analysis of biomarkers for crop performance concerning grain quality under stress conditions has been used to address food security (Dawid and Hille, 2018).

Metabolomics is an important platform for the interconnections between genotype and phenotype, which allows the interpretation of complex phenotypes for stress tolerance (Satrio et al., 2024). Utilization of the metabolomics platform with genomics helped researchers to identify the locus between genomics and metabolomics, resulting in the identification of metabolomics quantitative trait loci (mQTL) and metabolome-wide association studies (metGWAS) (Hamany Djande et al., 2020; Wei et al., 2018). This shows regions of the genome that control the concentration of metabolites produced by the plants during stress (Wei et al., 2018).

Beyond plant-intrinsic responses, metabolomics uniquely describes the chemical interactions between plants and beneficial microbes (Olanrewaju et al., 2024). Metabolomics helps to decipher processes such as how root exudates and microbial metabolites modulate nutrient acquisition, hormone signaling, immune priming, and stress tolerance (Anzano et al., 2022). This metabolic perspective allows for the rational design and creation of a microbiome resilient to fluctuations, successfully integrating the roles of plant metabolism with microbial functions for an improved agricultural yield under stress-prone settings (Rosier et al., 2018).

Moreover, metabolomics allows for the investigation of metabolic plasticity under combined and fluctuating stress conditions that are more relevant to field conditions than single-stress experiments (Olanrewaju et al., 2024). By elucidating how metabolic networks are rewired to maintain homeostasis and produce protective compounds under complex stress regimes, metabolomics delivers essential information for the breeding of crops resilient to climate variability (Wei et al., 2018; Yang et al., 2024b). Combined with other omics techniques, metabolomics enables a system perspective on stress acclimation and lays the basis for the translation of molecular information into robust crop phenotypes (Satrio et al., 2024).

### 3.2 Primary and secondary metabolites: response against abiotic stress

Plants developed different types of adaptations, such as changes in metabolism, under unfavorable environmental conditions (Ashapkin et al., 2020). Several plant metabolites may function as osmolytes and osmoprotectants, helping to reduce abiotic stress impacts. Primary metabolites are organic compounds that play an important role in an organism’s growth, development, and reproduction (Sangwan et al., 1994). They are important for the basic metabolic processes necessary for life by providing a carbon source from photosynthate and energy pathways (Sangwan et al., 1994). These primary metabolites also include proline amino acids and soluble and insoluble carbohydrates such as glucose, sucrose, starch, lipids, and organic acids (Bosco and Butnariu, 2022).

#### 3.2.1 Amino acids

Amino acids are the building blocks of proteins and other organic molecules like nucleic acids, which play an important role in how a plant reacts to various stresses. Amino acids are important for the production of proteins, like indole alkaloids, phenylpropanoids, polyamines, auxins, and glucosinolates, as well as for nitrogen fixation into glutamine during cold stress (Zandalinas et al., 2022). Signaling and molecular regulation depend on amino acids. Additionally, proline and other amino acids, well-known osmoprotectants, are produced when plants are subjected to different abiotic stresses (Singh et al., 1993; Hosseinifard et al., 2022). Proline acts as a molecular chaperone, protecting the protein structure and function. It also serves as a singlet oxygen quencher, which helps to balance ROS levels (Godoy et al., 2021). NADPH, ATP, and pyrroline-5-carboxylase synthetase (P5CS) are required for the reductive process of proline biosynthesis, as they catalyze the conversion of glutamate into pyrroline-5-carboxylate (P5C). Pyrroline-5-carboxylate reductases (P5CR) then reduce the P5C to proline (Salam et al., 2023). Another mechanism of proline biosynthesis occurs via the ornithine-δ-aminotransferase (OAT) enzyme, which is able to convert the ornithine into P5C (Mushtaq et al., 2025). A study showed that ornithine pathways were activated when the nitrogen supply was higher than the optimal during seedling growth. However, under a nitrogen deficiency, the glutamate (Glu) pathway is the dominant pathway for proline biosynthesis (Hosseinifard et al., 2022). It was found that tolerant species show more proline content than the sensitive species. Proline oxidase enzyme activity was increased in wheat lines, whereas the P5CR and OAT enzyme activities were reduced. Proline plays an important part in ROS scavenging by either increasing the enzyme activity involved in osmotic stress or protecting the glutathione-ascorbate cycle. Depending on the species, accumulation of proline shows variation under stress conditions. Another study found that excessive proline accumulation can cause P5C to produce more ROS and malondialdehyde and inhibit ABA and ethylene biosynthesis, lowers stress tolerance. In addition to proline, phenylalanine, tryptophan, and histidine were observed in maize hybrids under drought stress (Wang C. et al., 2025). It was found that the transgenic plants with proline metabolism genes enhanced plant tolerance against salinity and drought stress (Renzetti et al., 2024). The proline content of *Portulaca oleracea* L. was increased under 300 mM salinity stress (Hnilickova et al., 2021).

##### 3.2.1.1 The proline accumulation in relation to the metabolic trade-offs

Although proline accumulation is a sign of the plant’s resilience to abiotic stresses, studies have shown that its participation is very much dependent on the context of the situation and that it necessitates significant metabolic and oxidative trade-offs (Zulfiqar and Ashraf, 2023; Tiwari, 2024; Khan, 2025). The conversion of glutamate to proline is an energy-consuming process that requires a large amount of NADPH. During long or harsh stress periods, the overuse of reducing power for proline production may limit the activity of the other antioxidant systems, such as the ascorbate–glutathione cycle (Naliwajski and Skłodowska, 2021). This redirection may both deplete and disrupt the balance of overall redox homeostasis instead of strengthening it.

The metabolism of proline via proline dehydrogenase in mitochondria produces pyrroline-5-carboxylate (P5C), which is an intermediate that can lead to the generation of ROS if it accumulates in large quantities (Chalecka et al., 2021). Several investigations have indicated that the proline turnover during stress recovery or rehydration is not well controlled, and this results in increased levels of malondialdehyde and oxidative damage (Hanif et al., 2021). Thus, proline is not simply a universally protective metabolite; it is also a double-edged sword. In certain plant types, the buildup of proline has been linked to the reduction of ABA and ethylene biosynthesis, which may interfere with stress signaling and the development of adaptive responses (Wang et al., 2025a).

Another debate is the question of whether proline accumulation is an active tolerance mechanism or simply a metabolic symptom of stress injury. Even though tolerant genotypes usually have higher proline levels, the transgenic manipulation of proline biosynthesis does not always result in the consistent increase of growth or yield under field-relevant stress combinations (Raja et al., 2019; Renzetti et al., 2024). When subjected to combined or sequential stresses, especially drought and heat or salinity, proline accumulation may be so high that it causes carbon and nitrogen depletion, which then leads to penalties in growth and decreased reproductive fitness (Siddique et al., 2018).

Moreover, the functional results of proline buildup vary according to the tissue and developmental stage (Trovato et al., 2019). Proline might give a protective effect to the vegetative tissues by reinforcing the proteins and membranes, but in the reproductive organs, its excessive accumulation has been associated with decreased pollen germination and reduced seed formation in some plants (Siddique et al., 2018). Such findings underscore the necessity of examining the proline changes in the context of a larger metabolic network instead of treating them as an isolated indicator of stress (Renzetti et al., 2024). Taken together, these results showed that the buildup of proline is not advantageous by itself but is a sign of a well-managed equilibrium between osmoregulation, redox regulation, and metabolic cost. It will be necessary to bring together fluxomics, spatial metabolomics, and redox profiling conducted in single and combined stress situations to resolve these disputes and understand how proline metabolism can be best tuned to the stress-resilient breeding and engineering strategies.

#### 3.2.2 Lipids

The essential component of the plasma membrane is lipids, which function as the interface between the cell and its environment. On the basis of chemical structure and distinct hydrophilic and hydrophobic components, lipids can be classified into eight major types: glycerides, steroids, glycolipids, phosphoglycerides, fatty acids, polyketides, sphingolipids, and isoprenoids (Shiade et al., 2024). All biotic and abiotic stressors trigger lipid-mediated signaling (Table 1). Plants frequently use their plasma membrane, which is usually the source of lipid signaling, to detect these stimuli and convert the signal into biochemical metabolism (Hou et al., 2016). Signaling lipids like lipid kinases, phosphatases, and phospholipases are all acclimating enzymes. The major contributing signaling lipids are fatty acids, triacylglycerol, sphingolipids, phosphatides, lysophospholipids, oxylipins, triacylglycerols, and inositol phosphate (Table 1). It was found that the ability of signaling lipids to attract membrane molecular markers affects the shape and function of cellular proteins and metabolites. Phospholipase A (PLA) plays an essential role in the formation of lysophospholipids and fatty acids (Yaginuma et al., 2022). Lysophospholipids were found in small amounts in plant tissues, but they can be found in greater quantities under stressful conditions. Phospholipase A2 (PLA2) has been shown to increase the formation of certain elicitors in poppies, whilst lysophosphatidylcholine and lysophosphatidylethanolamine function as signal transducers in potato arbuscular symbiosis (Okazaki and Saito, 2014).

Lipid remodeling is a crucial adaptive process allowing plants to survive under harsh conditions like drought, salinity, heat, cold, and heavy metals, keeping their membranes stable, signaling, and metabolism in balance (Table 1). Similar to proline and other osmolytes, lipid changes are a way of giving stress protection, which is a costly process concerning metabolism and redox. The alterations in the structure of membranes caused by stress require quick adjustment of lipids to keep the membranes fluid, permeable, and able to function with proteins. A decisive factor of lipid remodeling is the control of fatty acids’ saturation degree. Heat stress in general increases the total of saturated fatty acids to limit the hyperfluidity of membranes. In contrast, cold and drought stresses boost unsaturated fatty acids that, to an extent, through the fatty acid desaturases, help in the regulation of the membrane’s flexibility (Hou et al., 2016). In addition, phospholipid-based signaling substances such as phosphatidic acid and lysophospholipids are formed quickly in large amounts under stress, leading to the activation of calcium signaling, MAP kinase (MAPK) cascades, and ABA-dependent transcriptional responses (Testerink and Munnik, 2011). The galactolipids of chloroplasts, especially monogalactosyldiacylglycerol (MGDG) and digalactosyldiacylglycerol (DGDG), are remodeled for the purpose of thylakoid membrane stabilization and photosynthesis support during drought and salinity stress (Moellering and Benning, 2011). On the other hand, lipid peroxidation produces oxylipins and reactive aldehydes that account for oxidative damage and carbon loss.

#### 3.2.3 Carbohydrates

The main components that provide support and energy to the plant biomass are carbohydrates, produced during photosynthesis. In plants, soluble carbohydrates may be crucial for metabolic processes as a carbon and energy source in a cell, as shown by Sangwan et al. (1994) (Table 1). Many factors can affect the amount of carbohydrates, and the process of photosynthesis is linked to the accumulation of carbohydrates. It has been found that by upregulating stress-related genes and downregulating growth-related genes, soluble sugars such as hexose and sucrose increase stress tolerance. However, in water stress conditions, the myoinositol content was reduced in barley roots (Salam et al., 2023). Furthermore, soluble carbohydrates like polymers of fructose molecules (fructans), oligosaccharides (raffinose and stachyose), and disaccharides (sucrose and trehalose), along with their associated metabolic enzymes, are crucial compatible osmolytes linked to the ROS during their assortment in plant tissues. However, the accumulation of carbohydrates in plants varies according to the type of stress (Godoy et al., 2021). In rice, overexpression of the trehalose biosynthesis gene can promote plant growth, reduce ROS damage, improve mineral balance, and boost trehalose buildup by increasing resistance to salinity and drought stress.

#### 3.2.4 Organic acids

In plants, organic acid metabolite changes take place under different abiotic stresses (Table 1). Citrate synthase enzyme converts oxaloacetate and acetyl-CoA into citric acid (CA), the first intermediate of the CAC cycle. In addition to being an antioxidant, CA can alleviate metals like aluminum, copper, and lead. Furthermore, it was reported a significant accumulation of calcium (Ca) in tomatoes cultivated under irrigation on calcareous soils, where elevated calcium levels enhanced the assimilation and uptake of Mn, Mg, and P in fruits, as well as Ca, Zn, Na, and N in leaves. Under Cd stress, antioxidant responses were enhanced by the increase in CAT, SOD, and POX enzyme activity in mustard (*Brassica juncea*) leaves by the exogenous applications of CA (Godoy et al., 2021).

## 4 Secondary metabolites and their response to stress

Secondary metabolites are crucial for defense mechanisms against biotic and abiotic factors. In higher plants, various kinds of secondary metabolites are produced from primary metabolic pathways, like the shikimate pathway, the pentose phosphate pathway, the CAC cycle, amino acid pathways, and glycolysis. The three main categories of secondary metabolites that plants produce are terpenes (terpenoids or isoprenoids), phenolic compounds (lignins, flavonoids/isoflavonoids, and tannins), and compounds that contain sulfur or nitrogen, like glucosinolates and alkaloids, respectively (Amiripour et al., 2021; Ali et al., 2024; Sabir et al., 2012; Yadav et al., 2014; Zandalinas et al., 2022). Aromatic amino acids such as tyrosine, phenylalanine, and tryptophan are precursors for the synthesis of secondary metabolites (Table 2). A variety of secondary metabolites from plants are crucial to the production of high food quality (taste, color, and smell), flavors, and odors and are used to produce, dyes, and pesticides. Under stressful conditions, plants use secondary metabolites, such as different signal molecules, which collectively play an important role in alleviating plant stress (Table 2). Abiotic stressors were found to cause phenyl amide formation and a significant buildup of polyamines in tobacco and beans, indicating a potential antioxidant role. The formation of anthocyanins is also triggered by a variety of environmental stressors, like UV, intense light, wounding, drought, blue light, pathogen attack, and deficiencies in sugar and nutrients (Table 2). The amount of secondary metabolite products depends on the environmental conditions and other entities, like the transcriptome and proteome, which affect various metabolic pathways that regulate the other metabolite production.

**TABLE 2:** Regulation of secondary metabolites under abiotic stress in plants.

Abiotic stress	Metabolite class	Plant	Pathway/compound type	Metabolite change	References
Drought	Terpenes	Maize (*Zea mays*)	Sesquiterpene (β-caryophyllene)	Significant increase in volatile emissions under drought stress	Vaughan et al. (2018)
Water deficit	Terpenes	Grapevine (*Vitis vinifera*)	Monoterpenes (linalool and geraniol)	Increased terpene concentration in berries	Brillante et al. (2018)
Water deficit	Terpenes	Lavender (*Lavandula* spp.)	Monoterpenes (1,8-cineole and linalyl acetate)	Increased essential oil content	Zheljazkov et al. (2014)
High light/temperature	Terpenes	Maritime pine (*Pinus pinaster*)	Monoterpenes (α-pinene and β-pinene)	Increased emission under combined stress	Loreto and Schnitzler (2010)
Heat stress	Terpenes	Tomato (*Solanum lycopersicum*)	Hemiterpene (isoprene)	Induced emission correlating with thermotolerance	Velikova et al. (2011)
Salinity	Terpenes	*Ginkgo biloba*	Diterpenes (ginkgolides)	Increased accumulation in leaves	Shan and Jin (2025)
UV-B radiation	Terpenes	Oregano (*Origanum vulgare*)	Monoterpenes (carvacrol and thymol)	Increased concentration as a photoprotective response	Ali et al. (2024)
Water stress	Terpenes	Rosemary (*Rosmarinus officinalis*)	Monoterpenes (α-pinene and 1,8-cineole)	Increased concentration in leaves	Sarmoum et al. (2019)
Drought	Terpenes	Sweet wormwood (*Artemisia annua*)	Sesquiterpene lactone (artemisinin)	Enhanced accumulation under drought	Yadav et al. (2014)
Heat stress	Terpenes	Peppermint (*Mentha × piperita*)	Monoterpenes (menthol and menthone)	Altered ratio and total yield	Chrysargyris et al. (2016)
Drought	Terpenes	Lemongrass (*Cymbopogon* spp.)	Monoterpenes (citral, geraniol, and geranyl acetate)	Enhanced accumulation under drought conditions	Sangwan et al. (1994)
Cold stress	Terpenes	Tea (*Camellia sinensis*)	Sesquiterpene ((E)-nerolidol)	Increased volatile emission under cold	Yang et al. (2018)
Salinity	Terpenes	Sweet basil (*Ocimum basilicum*)	Phenylpropene/monoterpene	Increased essential oil yield	Caliskan et al. (2017)
Drought	Terpenes	Qinghasu (*Artemisia annua*)	Mono- and sesquiterpenoids	Enhanced terpenoid content	Yadav et al. (2017)
Ozone stress	Terpenes	Norway spruce (*Picea abies*)	Hemiterpene (isoprene)	Induced emission as an antioxidant response	Kivimäenpää et al. (2022)
Waterlogging	Terpenes	Lemon (*Citrus × limon*)	Monoterpene (limonene)	Accumulation in roots with a protective role	González-Mendoza et al. (2018)
Heavy metal (Cd)	Terpenes	Castor bean (*Ricinus communis*)	Diterpene derivative (ricinoleic acid)	Altered biosynthesis in seeds	Dubey et al. (2020)
Water deficit	Terpenes	Geranium (*Pelargonium graveolens*)	Monoterpenes (citronellol and geraniol)	Increased essential oil percentage	Amiri et al. (2015)
Drought	Terpenes	Turmeric (*Curcuma longa*)	Sesquiterpene (ar-turmerone)	Elevated concentration in rhizomes	Bharadwaj et al. (2024)
High temperature and CO_2_	Terpenes	*Eucalyptus* spp.	Monoterpene (1,8-cineole)	Interactive effects on emission rates	Singh (2024)
UV-C radiation	Terpenes	Rice (*Oryza sativa*)	Diterpenes (phytocassanes and momilactones)	*De novo* synthesis of phytoalexins	Kariya et al. (2024)
Ozone stress	Terpenes	Tobacco (*Nicotiana tabacum*)	Sesquiterpenes (duvatrienes)	Induced emission under oxidative stress	Zeng et al. (2021)
High light intensity	Terpenes	Sage (*Salvia officinalis*)	Monoterpenes (thujone and camphor)	Increased synthesis and accumulation	Hazrati et al. (2021)
Salinity	Terpenes	Coriander (*Coriandrum sativum*)	Monoterpene (linalool)	Increased content in leaves and seeds	Amiripour et al. (2021)
Cold stress	Terpenes	Patchouli (*Pogostemon cablin*)	Sesquiterpene (patchouli alcohol)	Reduced yield but altered enzyme activity	Zhang et al. (2023)
Mechanical wounding	Terpenes	Japanese cedar (*Cryptomeria japonica*)	Sesquiterpenes (α-cedrene and cedrol)	Rapid induced emission	Kivimäenpää et al. (2024)
Drought	Terpenes	Thyme (*Thymus vulgaris*)	Monoterpenes (thymol and γ-terpinene)	Increased proportion of thymol chemotype	Hashemifar et al. (2025)
Heat stress	Terpenes	Cannabis (*Cannabis sativa*)	Mono- and sesquiterpenes	Increased total terpene concentration	Yeshi et al. (2022)
Nutrient deficiency (P)	Terpenes	Sunflower (*Helianthus annuus*)	Sesquiterpene lactones	Increased concentration in leaves	Kusvuran (2021)
Mechanical damage	Terpenes	Giant fir (*Abies grandis*)	Monoterpenes (α-pinene and β-phellandrene)	Induced systemic emission	López-Villamor et al. (2021)
UV-B radiation	Terpenes	Lemon balm (*Melissa officinalis*)	Monoterpenes (citronellal and geranial)	Increased essential oil yield and antioxidant activity	Akram et al. (2024)
Salinity	Terpenes	Neem (*Azadirachta indica*)	Triterpenoid (azadirachtin)	Upregulation of biosynthetic genes	Bhambhani et al. (2017)
Boron deficiency	Terpenes	Black spruce (*Picea mariana*)	Monoterpene (bornyl acetate)	Altered terpene synthase activity	Shelyakin et al. (2022)
Waterlogging	Terpenes	Jasmine (*Jasminum* spp.)	Mono- and sesquiterpenes	Altered floral volatile profile	Ali et al. (2024)
Heavy metal (Cu)	Terpenes	Chili pepper (*Capsicum annuum*)	Sesquiterpene phytoalexin (capsidiol)	Induced synthesis under copper stress	Zeng et al. (2021)
Water stress	Terpenes	Valerian (*Valeriana officinalis*)	Sesquiterpene (valerenic acid)	Altered biomass and essential oil composition	Mustafavi et al. (2016)
Heavy metal (Cd)	Steroids	Ashwagandha (*Withania somnifera*)	Steroids (withanolides and sterols)	Induced accumulation	Mishra et al. (2017), Mishra et al. (2024)
Salinity	Steroids	Ashwagandha (*Withania somnifera*)	Steroids (withanolides)	Increased accumulation under *in vitro* salt stress	Sabir et al. (2012)
Cold stress	Steroids	*Arabidopsis thaliana*	Triterpenes (sitosterol and campesterol)	Altered membrane sterol composition under chilling	Feng et al. (2024)
Drought	Saponins	Alfalfa (*Medicago sativa*)	Triterpenoid saponin pathway	Enhanced root growth, water uptake, and ROS reduction	Zhang and Shi (2018)
Drought	Anthocyanins	Maize (*Zea mays*)	Anthocyanin biosynthesis	Reduced oxidative stress and improved osmotic adjustment	Seleiman et al. (2021)
Heat stress	Carotenoids	Tomato (*Lycopersicon esculentum*)	Carotenoid biosynthesis	Stabilization of membranes and photosynthetic protection	Spicher et al. (2016)
Drought	Carotenoids	Maize (*Zea mays*); tomato (*Solanum lycopersicum*)	Carotenoid biosynthesis; ABA biosynthesis pathway	Increased lutein and β-carotene for ROS scavenging and membrane stability; elevated ABA levels regulating stomatal closure and water conservation	Xiang et al. (2019), Rocha Júnior et al. (2023)
Salinity	Carotenoids	Rice (*Oryza sativa*)	Xanthophyll cycle activation	Enhanced zeaxanthin and violaxanthin levels dissipate excess light energy and reduce photodamage	Chauhan et al. (2023)
Heat stress	Carotenoids	Chickpea (*Cicer arietinum*)	Carotenoid biosynthesis	Enhanced zeaxanthin accumulation mitigates thermal damage to photosynthetic machinery	Kumar et al. (2020)
Cold stress	Carotenoids	*Arabidopsis thaliana*	ABA biosynthesis via NCED	Increased ABA levels enhance cold stress tolerance and ROS scavenging	Qian et al. (2024)
Oxidative stress	Carotenoids	Tobacco (*Nicotiana tabacum*)	Carotenoid biosynthesis	Increased carotenoids (lycopene and lutein) contribute to ROS neutralization	Rasmus and Kozłowska et al. (2023)
Salinity	Tannins	Grape (*Vitis vinifera*)	Phenylpropanoid pathway	Improved water retention and reduced ion toxicity	Siemińska-Kuczer et al. (2022)
Heavy metals	Alkaloids (Nicotine)	Tobacco (*Nicotiana tabacum*)	Alkaloid biosynthesis	Chelation of heavy metals reduces bioavailability	Selwal et al. (2023)
Drought	Alkaloids	Periwinkle (*Catharanthus roseus*)	Terpenoid indole alkaloid (TIA) pathway	Enhanced vindoline and catharanthine production to counteract ROS and maintain cellular integrity	Nishanth et al. (2018)
Salinity	Alkaloids	Barley (*Hordeum vulgare*)	Gramine biosynthesis	Increased gramine accumulation conferring protection against oxidative stress	Kotzamani et al. (2021)
Cold stress	Alkaloids	Coffee (*Coffea arabica*)	Purine alkaloid (caffeine) biosynthesis	Increased caffeine accumulation stabilizes membranes and prevents oxidative damage	Acidri et al. (2020)
Waterlogging	Alkaloids	Rice (*Oryza sativa*)	Indole alkaloid biosynthesis	Increased serotonin levels mitigate hypoxia-induced oxidative stress	Liu et al. (2023)
Oxidative stress	Alkaloids	Potato (*Solanum tuberosum*)	Glycoalkaloid biosynthesis	Increased solanine and chaconine production to neutralize ROS and stabilize cellular components	Rainio et al. (2020)
Drought	Phenolics	Wheat (*Triticum aestivum*); olive (*Olea europaea*)	Phenylpropanoid pathway; flavonoid biosynthesis	Increased phenolic acids (ferulic acid and *p*-coumaric acid) for ROS scavenging and membrane stabilization; enhanced flavonoids (quercetin and luteolin derivatives) protect against oxidative damage	Laddomada et al. (2021), Rao et al. (2025)
Salinity	Phenolics	Rice (*Oryza sativa*); tomato (*Solanum lycopersicum*)	Lignin biosynthesis via the phenylpropanoid pathway; flavonoid biosynthesis and phenolic acid pathways	Increased lignin deposition strengthens cell walls and reduces ion permeability; enhanced chlorogenic acid and rutin levels for ROS detoxification	Jia et al. (2022)
Heat stress	Phenolics	*Pinellia ternata*	Flavonoid pathways	Inhibition of chlorogenic acid, pelargonidin, cyanidin, and epigallocatechin biosynthesis under heat stress	Guo et al. (2023)
Oxidative stress	Phenolics	Soybean (*Glycine max*)	Phenolic acid and flavonoid biosynthesis	Accumulation of isoflavones (e.g., genistein) and phenolic acids for ROS scavenging	Król-Grzymała and Amarowicz et al. (2020)
Drought	Lignin	Wheat (*Triticum aestivum*)	Phenylpropanoid–lignin pathway	Strengthened cell walls prevent water loss	Liu et al. (2018)
Heavy metals	Polyphenols	Sunflower (*Helianthus annuus*)	Polyphenol metabolism	Chelation of heavy metals and mitigation of oxidative damage	Saidi et al. (2021)

### 4.1 Terpenoids

#### 4.1.1 Regulation of terpenoid biosynthesis in response to abiotic stress in plants

Terpenes are the largest and most structurally diverse group of natural plant products, consisting of more than 65,000 known compounds (Xavier et al., 2023). They play key ecological roles: They shield plants from herbivores and lure pollinators. Terpenes are derived from the five-carbon isotopic precursors isopentenyl diphosphate (IPP) and dimethylallyl diphosphate (DMAPP), which are produced by two independent pathways, the mevalonate (MVA) pathway and the 2-C-methylerythritol-4-phosphate (MEP) pathway. Following the MVA pathway, farnesyl diphosphate (FPP, C15) is a hub metabolite in the formation of sesquiterpenes, triterpenes, and sterols. In contrast, the MEP pathway generates IPP and DMAPP from pyruvate and glyceraldehyde-3-phosphate, which are condensed in the final common step by a geranyl diphosphate synthase (GPS) or a geranylgeranyl diphosphate synthase (GGPPS) to form precursor monophosphates for mono-, di-, or tetraterpenes: GPP; C10 terpenes: GGPP; C20 terpenes (Couillaud et al., 2021; Shang et al., 2024; Sun et al., 2023). Subsequently, terpene synthases (TPSs) catalyze the cyclization of these intermediates, providing a wide variety of terpenoid skeletons, determining the structural and functional diversity of terpenes (Tholl et al., 2023).

Terpenoids, including monoterpenes, sesquiterpenes, and diterpenes, are important plant secondary metabolites produced from isoprene units. They are synthesized in different plant secretory organs and are involved in diverse aspects of the relationship between plants and the environment, especially under abiotic stress (Saini et al., 2025; Semmar, 2024). As natural antioxidants and signal molecules, terpenoids can attenuate oxidative stress through scavenging ROS and increasing the activity of antioxidant enzymes (Sangwan et al., 2024). As a result of their natural properties, the emissions are highly dependent on environmental conditions such as temperature, humidity, and solar insolation. Apart from abiotic stress alleviation, volatile terpenoids are involved in ecological signaling and antifeedant/anti-invasive compounds that contribute to the general stress tolerance and surge capacity of plants (Ninkuu et al., 2021). Terpenoids play crucial roles in plant acclimation to abiotic stress, acting as antioxidants and signaling molecules to maintain cellular homeostasis and synchronize stress responses (Li et al., 2023). The compounds work by interfusing environmental perception, metabolic control, and cell protection to promote plant resistance to stresses such as heat, drought, and oxidative damage (Li et al., 2023; Garcia-Caparros et al., 2021; Chen Z. et al., 2022). Future research efforts should focus on hormonal cross talk, combined stress interactions, and severe chemical diversity found within terpenoids to reveal new networks of regulation and prompts in a few or metabolically engineered/selectively bred stress-tolerant crop varieties to grow under expected desertification due to climate change (Rosenkranz et al., 2021).

#### 4.1.2 Regulatory factors influencing terpenoid biosynthesis

**FIGURE 4:**
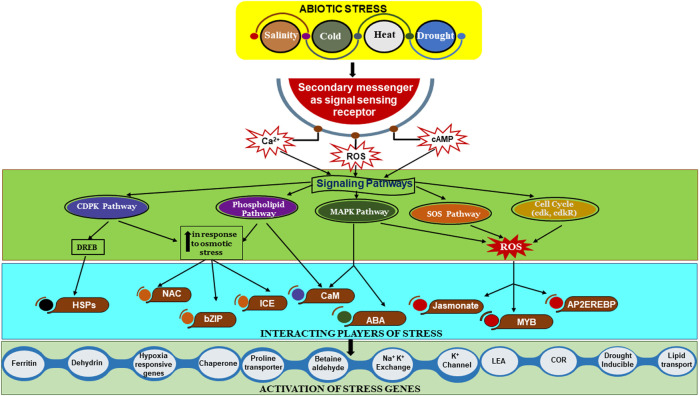
Abiotic stress perception and signal transduction leading to stress-responsive gene activation in plants. ROS, reactive oxygen species; CDK, cyclin dependent kinase; CDPK, calcium-dependent protein kinase; COR, cold responsive; MAPK, mitogen-activated protein kinase; SOS, salt overlay stress; ICE, inducer of C-repeat binding factor (CBF) expression; PLD, phospholipase D; CDKR, cyclin-dependent kinase regulatory; CaM, calmodulin; CaCmpk, Ca calmodulin-dependent protein kinases; EREBPs, ethylene-responsive element-binding proteins; ABA, abscisic acid; AP2EREBP, AP2(APETALA 2); NAC, NAM, ATAF1/2, and CUC2 (No Apical Meristem, Arabidopsis Transcription Activation Factor, and Cup-Shaped Cotyledon); bZIP, basic leucine zipper domain; LEA, late embryogenesis abundant; PLC, phospholipase C.

**FIGURE 5:**
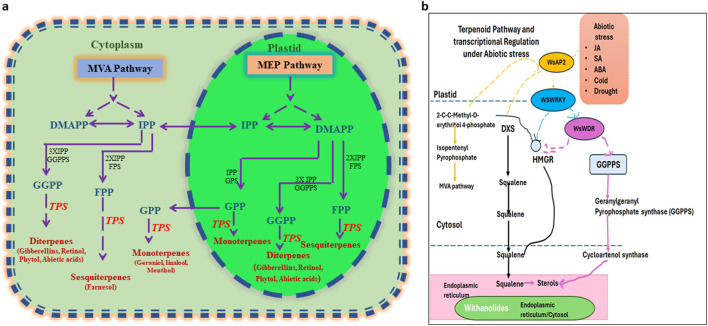
**(a)** Overview of terpene biosynthetic pathways in plants. **(b)** Transcriptional regulation and signaling of terpene synthesis pathways under abiotic stress.

The control of volatile terpenoid formation in plants is coordinated by interactions among key enzymes, transcription factors, and plant hormones (Rosenkranz et al., 2021). HMGR (3-hydroxy-3-methylglutaryl-CoA reductase), DXS (1-deoxy-D-xylulose-5-phosphate synthase), and DXR (1-deoxy-D-xylulose-5-phosphate reductoisomerase) are important to regulate metabolic fluxes in terpenoid biosynthesis (Jadaun et al., 2017; Mishra et al., 2017). Their action affects the rate of precursor generation, which ultimately dictates terpenoid productivity (Noushahi et al., 2022). The MYB, bHLH, and WRKY transcription factors have been shown to regulate the biosynthetic genes in response to environmental signals (Jadaun et al., 2020). In addition, plant hormones, including jasmonic acid (JA), ethylene (ET), and abscisic acid (ABA), participate in the signaling networks that modulate terpenoid biosynthesis (Lv et al., 2021). Among these, JA is the most significant because it mediates the expression of biosynthetic genes as well as terpenoid accumulation under abiotic and biotic stress conditions to contribute to plant tolerance or response against the stresses (Li Y. et al., 2024; Lv et al., 2021). The regulation of terpenoid biosynthesis in plants in response to abiotic stress is a complex network, which includes the early signaling stress events, the interplay with hormones, transcriptional/epigenetic control, and post-translational modifications of its metabolic enzymes (Mishra et al., 2024) (Figures 4, 5).

#### 4.1.3 Biosynthetic pathways and key enzymes

Most terpenes are biosynthesized *via* two independent metabolic routes: the mevalonate (MVA) pathway and the 2-C-methyl-D-erythritol-4-phosphate (MEP) pathway (Noushahi et al., 2022; Singh et al., 2023). The MVA pathway produces precursors for sesquiterpenes and triterpenes in the cytosol, while the MEP pathway works in plastids and makes a precursor for monoterpenes, diterpenes, and tetraterpenes (Singh et al., 2023). Key regulatory enzymes in this pathway are 1-deoxy-D-xylulose 5-phosphate synthase (DXS) and 1-deoxy-D-xylulose 5-phosphate reductoisomerase (DXR) of the MEP pathway and 3-hydroxy-3-methylglutaryl-CoA reductase (HMGR) of the MVA pathway (El Amerany, 2024). Moreover, several isoforms of terpene synthase (TPS) are involved in the diversification of terpenoid skeleton (Singh et al., 2023). The accumulation and activities of these cascaded products are tightly regulated during abiotic stress, typically increasing the production of defensive terpenoids, which contribute to stress tolerance and defense (EI Amerany et al., 2024).

#### 4.1.4 Defensive functions of terpenoids against abiotic stress

Terpenoids serve as a dual dynamic modulator of plant defense in response to abiotic stress, both protecting plants from oxidative damage and strengthening systemic stress resistance (Holopainen et al., 2025). In addition, volatile terpenoids, including isoprene, monoterpenes, and sesquiterpenes, can efficiently scavenge highly reactive oxygen species (ROS) such as hydroxyl radicals (OH) and singlet oxygen, which interfere with lipid peroxidation, protein oxidation, and chloroplast membrane destruction. This antioxidative role keeps the efficiency of photosynthesis, especially under heat and oxidative stress (Holopainen et al., 2025; He et al., 2023). In addition, isoprene reacts with O_3_ in the atmosphere, depleting it within mesophyll tissues and preventing harmful reactive nitrogen species, which may lower oxidative load (He et al., 2023).

In addition, isoprene-emitting plants often have high reduced ascorbate levels, suggesting that, because of its direct scavenging ability, they rely less on enzymatic antioxidants (Srikanth et al., 2024). Isoprene oxidation products serve as secondary signals that induce the expression of antioxidant defense genes and systemic protectives (Ahmad et al., 2023). Volatiles are airborne chemical signals that prepare a defense at the local and systemic (neighboring plants or tissues) levels, achieving systemic stress tolerance. Increased emission of these substances under moderate drought or heat treatment also leads to better physiological resistance, such as rooting and drought tolerance, in plant species such as *Salvia nemorosa* and *Torreya grandis* (Papafotiou et al., 2021; Zhang B. et al., 2025). Together, these mechanisms highlight the multiple protective and signaling roles of terpenoids as biochemical weapons and chemical mediators in plant response to abiotic stress.

#### 4.1.5 Terpene emission and plant responses

Strong relationships between environmental conditions and isoprene emission in plants have been demonstrated by recent study (Malone et al., 2023). Isoprene emission exhibits strong sensitivity to water stress, temperature, and light intensity; temperature and light are the near-determining factors for the rate and the diurnal pattern of emission via their impact on the photosynthetic performance during active metabolism and precursor availability (Zhang et al., 2022a). Temperature has a large impact on post-illumination isoprene emissions of oak and poplar, suggesting that the synthesis and the emission of isoprene are controlled by thermal conditions upon light exposure (Furnell et al., 2024). A thorough mechanistic investigation of the temperature response of isoprene emission explained its variation by substrate (e.g., DMAPP) availability and enzyme activity within the MEP pathway was reported by Souza et al. (2025). Together, these studies demonstrate that isoprene release is a tightly regulated physiological response driven by numerous environmental and metabolic cues contributing to overall plant stress adaptation and thermal profile.

#### 4.1.6 Early stress signaling and cascade activation

Abiotic stress induces prompt production of ROS such as superoxide anions, hydrogen peroxide, and hydroxyl radicals mainly due to photosynthetic and mitochondrial perturbations (Sachdev et al., 2021). Excess ROS lead to oxidative damage, but the same species also serve as signaling molecules. In parallel, stress generates transient cytosolic calcium influxes (Ca^2+^ flux), known as calcium bursts. ROS and Ca^2+^ signals construct a positive (self-sustaining) loop: ROS activates inward-rectifying Ca^2+^-permeable channels (IMCC) to promote Ca^2+^ influx, while Ca^2+^ triggers NADPH oxidases that produce ROS. This primary chain of events involves a quick transmission of signals that are transmitted within and among the plant cells, evoking secondary hormonal and gene expression responses (Hendricks et al., 2021).

#### 4.1.7 The role of monoterpenes and sesquiterpenes in plant defense

Monoterpenes and sesquiterpenes play a critical role in improving the antioxidant capacity of plants under different abiotic stress conditions. Monoterpenes (including α-pinene and β-pinene) boost the thermal stability of the photosynthetic apparatus, which can help to sustain efficient photosynthesis under heat stress (El Amerany, 2024). In addition, these volatile compounds could react directly with ozone (O_3_), thus decreasing mesophyll oxidative damage and secondary reactive species production (Singh et al., 2023). Because of their greater hydrophobicity, sesquiterpenes intercalate into cellular lipid bilayers and help to maintain membrane integrity by reducing the lipid peroxidation. Both monoterpenes and sesquiterpenes have potent antioxidant effects and cross talk with stress signaling pathways, leading to redox homeostasis and better physiological status during environmental stresses (Shan and Jin, 2025) (Figure 4).

As substantiated by several reports, volatile terpenoids form a first line of defense and protect plants from stress. Research showed that isoprene emissions in *Platanus orientalis* have a protective role against high temperature stress by preventing cell membrane damage and photochemical efficiency (Bertamini et al., 2019). Similarly, monoterpene emissions *in Cinnamomum camphora* play a crucial role in thermotolerance, implying the photoprotective properties of monoterpenes against heat stress (Zuo et al., 2017). These findings in combination highlight the complexity of volatile terpenoids for improved plant resilience.

### 4.2 Phenolic compounds

The phenol group is a secondary product produced by plants, a chemically heterogeneous hydroxyl functional group on an aromatic ring (Lattanzio, 2013). It is an important parameter regulated in plant defense mechanisms against stress conditions (Table 2). Phenolics mitigate oxidative stress due to their antioxidant properties.

Phenolic compounds are synthesized through the phenylpropanoid and shikimic acid pathway. Different stress conditions change the phenolic metabolites and affect metabolic pathways, as shown in Table 2. Naringenin is a flavanone intermediate that is widely found in plants and is the source of isoflavonoids (Shen et al., 2025). It is vital for plant growth and defensive response. Production of these flavonoids appears to be an effective defense against ROS. This may be because the phenolic compounds, which may serve as substrates for other peroxidases, are the initial line of defense against environmental stress. The biosynthesis of flavonoids is a well-established pathway for the production of natural dietary antioxidants (Muthusamy and Lee, 2024). Phenylalanine is involved in the generation of flavonoids via the shikimate pathway.

### 4.3 Alkaloids

Approximately 20% of vascular plant species have a large family of nitrogen-containing secondary metabolites, which are most commonly found in herbaceous dicots and less commonly found in a few gymnosperms and monocots (Muthusamy and Lee, 2024; Chen D. et al., 2022). Alkaloids are synthesized from amino acids, such as aspartic acid, tyrosine, lysine, and tryptophan. Alkaloids defend plants against heavy metal toxicity and salinity by changing ion channels, which aids them to tolerate the stress (Table 2). Subgroups of alkaloids, which include isoquinoline, indole, purine, tropane, imidazole, phenylethylamine, and terpenoids, can be distinguished by their basic heterocyclic nucleus (Jadaun et al., 2017; Muthusamy and Lee, 2024).

### 4.4 Carotenoids

The development and defense of a variety of biological processes in plants, like the formation of photosystems, the light-harvesting antenna complexes for photosynthesis and photoprotection, and the regulation of growth and development, depend on carotenoids and on apocarotenoids (Muthusamy and Lee, 2024). According to Sun et al. (2022), carotenoids and their products are necessary for the generation of phytohormones like strigolactones (SLs) and abscisic acid (ABA), which act as signaling molecules mediating developmental and environmental responses. Carotenoids are classified into two categories: unsaturated carotenes (C40 hydrocarbons) and their oxygenated derivatives known as xanthophylls. Apocarotenoids mediate signaling and the connections between the environment and the plant under stress conditions. Exposure to environmental stress changes the plant carotenoid composition, similar to other metabolites (Table 2).

## 5 Impact of abiotic stress on secondary metabolites

Plant secondary metabolites (PSMs) are synthesized and accumulated in response to environmental factors and have important roles in plant adaptation to abiotic stresses (Khare et al., 2020). Alterations in growth conditions and imposition of stressful environments like drought, salinity, temperature extremes, and heavy metals are known to induce changes in the metabolic pathways and subsequently increase production of different PSMs (Yeshi et al., 2022). These molecules, including phenolics, flavonoids, alkaloids, and terpenes, act as antioxidants (Table 2), osmoprotectants, or signaling molecules in plant systems, where they protect plants from oxidative stress and maintain cellular homeostasis. Therefore, the formation of PSM is frequently induced by abiotic stress as an important adaptive and defensive reaction (Yeshi et al., 2022).

### 5.1 Effect of heavy metals on the production of secondary metabolites

Plants have been observed to be impacted by the heavy metal stress. It has been reported that heavy metal exposure alters the biosynthesis of photosynthetic pigments, sugars, proteins, and nonprotein thiols. These metals can alter secondary metabolism pathways and, consequently, the production of bioactive substances. Silver, europium, lanthanum, cadmium, and oxalates are some of the metal ions that regulate secondary metabolite biosynthesis. Nickel, an essential element for the urease enzyme, plays an important role in plant growth and metabolism (Gupta and Pathak, 2025). Additionally, copper (Cu^2+^) and cadmium (Cd^2+^) have been shown to increase the accumulation of shikonin-like metabolites (Ghazagh et al., 2023).

The analysis of *Hypericum perforatum* under cadmium stress showed an increase in phenolic acids such as ferulic acid, but flavonoids such as epicatechin and procyanidin were found to be decreased in roots and shoots (Giebułtowicz et al., 2024). The level of activity of phenylalanine ammonia-lyase (PAL), the first enzyme in the phenylpropanoid pathway, was positively related to heavy metal accumulation, indicating that genes encoding PAL and related enzymes are upregulated by metal stress (González-Mendoza et al., 2018). To protect themselves under heavy metal stress, plants produce phenolic acids, which work as antioxidants and chelating compounds without moving to the synthesis of flavonoid or anthocyanin. For instance, *Matricaria chamomilla* roots overproduce phenolics to chelate cadmium and decrease its toxicity (Kolodziejczyk-Czepas et al., 2015).

There is a fine balance between oxidants and antioxidants in plants that prevents the overaccumulation of ROS. Phenolic and terpenoid secondary metabolites represent important antioxidants for the scavenging of ROS to prevent oxidative damage (Saleem et al., 2022). The radicals are stabilized by the antioxidant activity of flavonoids due to their hydroxyl (–OH) groups. Soluble phenolic content was stimulated by cadmium in *Populus × canescens*, with particular accumulation recorded in bark tissues, suggesting organ-specific metabolic response of the proanthocyanidin pathway to heavy metal stress (Kebert et al., 2022). These results support the adaptive significance of secondary metabolites against heavy metal detoxification and oxidative stress (Sharma et al., 2025).

### 5.2 Effect of temperature stress on secondary metabolite production

Temperature is an important abiotic factor that affects plant growth, metabolism, and the synthesis of secondary metabolites. Extreme heat and cold both induce stress, which can change physiological, biochemical, and molecular mechanisms in plants (Qaderi et al., 2023). Heat stress-induced leaf senescence and decreased photosynthesis efficiency, which can result in limited CO_2_ assimilation, resulting in a negative impact on the growth and yield production. It also involves secondary metabolism: some plants produce more secondary metabolites (SM) in response to heat stress, while others produce fewer. For example*, Panax quinquefolius* displays greater amounts of ginsenosides in increased temperature, while other species demonstrate reduced SM content (Rehman et al., 2024). Elevated temperatures favor the production of osmolytes, phenolic compounds, flavonoids, and sugars, which are involved in osmoregulation, membrane stabilization, and ROS scavenging (Upadhyay and Srivastava, 2019; Rao et al., 2025). Heat shock proteins (HSPs) and compatible solutes, such as glycine betaine and γ-aminobutyric acid (GABA), are important in protecting cellular components and conferring thermotolerance (Zhang et al., 2022c).

Cold stress is harmful, particularly in temperate plants, suppressing metabolism, impeding water exchange, and causing desiccation (Manasa et al., 2022). To survive freezing stress, plants encounter cold acclimation, which results in the accumulation of cryoprotective molecules such as sugars, sugar alcohols, and nitrogenous compounds (Thakur and Nayyar, 2012).

These agents protect membranes and proteins, thus minimizing freezing damage (Feng et al., 2025). Cold stress contributes to phenolic metabolism and the accumulation of phenolic acids and flavonoids, which can serve as antioxidants and strengthen cell walls through lignin synthesis and suberin deposition (Hassan et al., 2021). The phenolic acid and isoflavonoid levels were increased by cold treatment in soybean roots, while *Capsicum annuum* decreased photosynthesis and growth under freezing temperatures (Qian et al., 2024).

Polyamines such as putrescine and spermidine are crucial in cold resistance (Wang Y. et al., 2024). Melatonin attenuates cold-induced apoptosis by modulating polyamine synthesis. Cold stress, even in the absence of freezing, disturbs photosystems and increases ROS concentration (Rahman et al., 2024). Plants acclimate by adjusting hormones such as abscisic acid (ABA) and increasing cold-regulated genes to improve resistance (Raza et al., 2023). Both high and low temperature extremes greatly influence secondary metabolism by inducing the production of stress protective metabolites that help plants to survive and acclimatize in temperature-intensive environments (Table 2).

### 5.3 Effect of salt stress on secondary metabolite production

Salt stress is an important abiotic factor in restricting plant growth, yield, and formation of bioactive compounds (Toscano et al., 2019). This disrupted cell homeostasis results in lowered photosynthetic rate, compromised CO_2_ fixation, and reduced cell enlargement (Noman et al., 2018). Plants respond to these stresses by synthesizing secondary metabolites, including phenolics, flavonoids, and polyamines, as antioxidants and osmoprotectants (Saini et al., 2024; Ahlawat et al., 2024). The aggregations of phenolics when the plant is subjected to salt stress; the aggregations contribute to ROS scavenging and redox balance at the same time (Saleem et al., 2022).

Changes in polyamine metabolism due to salinity stress have been reported as part of plant involvement in stress adaptation (Choudhary et al., 2023). The effects of various salts differ: potassium chloride (KCl) treatments led to increased phenolic and flavonoid contents in the leaves of *Cynara cardunculus* more than sodium or calcium chloride treatments (Lucini et al., 2016). Worldwide, saline soils are a threat to almost 20% of the total irrigated land, which is indeed a major constraint to food production. Plants produce compatible solutes like glycine betaine, trehalose, mannitol, and sucrose to counter the osmotic imbalance, while enzymes such as superoxide dismutase (SOD), catalase (CAT), and ascorbate peroxidase (APX) together with antioxidants like ascorbate (ASC), glutathione (GSH), and carotenoids are activated to protect the cellular components defending against salinity (Yang et al., 2021).

### 5.4 Effect of drought stress on secondary metabolite biosynthesis

Drought stress is one of the most important abiotic factors limiting plant growth, productivity, and survival. Proteins, lipids, carbohydrates, and nucleic acids are irreversibly damaged by it, and the plant height, root system, and leaf area are disturbed (Ghasemi et al., 2023). Physiologically, drought reduces osmotic potential, stomatal conductance, photosynthesis, and transpiration rate, resulting in a reduction of yield that is a major threat to global food security. As a means of coping, plants use morphological, biochemical, and physiological strategies that contribute to drought tolerance (Ahmad et al., 2023).

Plants under drought commonly synthesize secondary metabolites (SMs), including flavonoids, phenolic acids, and essential oils, to protect themselves from oxidative stress (Ahmad et al., 2023). Examples include the higher content of flavonoids and phenolics in leaves and increased total phenolics (TP) in *Salix* (willow) under drought and increased total flavonoids in *Glechoma longituba*. In the same context, under drought stress, *A. annua* was reported to produce the most effective SMs under drought conditions (Jia et al., 2020; Yadav et al., 2014; Wang R. et al., 2024). In contrast, some metabolites, such as saponins in *Chenopodium quinoa*, decreased under severe water deficit (Huan et al., 2022).

To alleviate oxidative damage induced by drought, plants accumulate osmoprotectants (such as proline, glycine betaine, sorbitol, and sucrose) to ameliorate osmotic effects and stabilize proteins and membranes. Enzyme antioxidants, including SOD, CAT, and peroxidases, scavenge superoxide, hydrogen peroxide, and other reactive oxygen species (Ighodaro and Akinloye, 2018). Plant hormones such as ABA, salicylic acid, cytokinins, and even GA play a role in stress response that allows the plant to survive reduced water availability while it reroutes its metabolites (Singh and Roychoudhury, 2023).

### 5.5 Effect of light stress on synthesis of secondary metabolites

Light is one of the most important environmental parameters controlling plant growth, development, and secondary metabolite (SM) biosynthesis. Diverse light intensities, wavelengths, and photoperiods greatly affect or stimulate metabolic processes and the synthesis of bioactive compounds (Wu et al., 2025). *Zingiber officinale* cell cultures showed that synthesis of gingerol and zingerone is increased by light, as are those of zingiberene, with an increase in phenolic accumulation at higher light intensity. In the same way, long light periods increase coumarin content, which decreases during short photoperiods (Gong et al., 2023). In *P. quinquefolius* (American ginseng), ginsenoside concentration in roots is higher when the plant has been exposed to sunlight for a longer period of time (Liu et al., 2022).

Metabolite production is influenced by the quality of light to which plants are exposed. Blue light has been reported as being most effective in promoting the production of SMs in *Scutellaria lateriflora*, and synergistic effects of red/blue LED have been observed for metabolite synthesis in *Peucedanum japonicum* (Costine et al., 2022). Biomass and SM accumulation changes with light versus dark incubation in *Artemisia absinthium* cultures (Sánchez-Ramos et al., 2022).

Ultraviolet (UV) light also affects metabolism; UV-B irradiation decreases chlorophyll, but increases flavonoids and phenylalanine ammonia-lyase (PAL) activity. For example, UV light increases root flavonoids in *Pisum sativum* and rutin and quercetin content in *Fagopyrum esculentum* (Innes et al., 2019). In addition, photo-periodic changes modulate the endogenous indoleamines, including serotonin and melatonin, in algae. Light, in general, is an energy source and signal regulator affecting primary and secondary metabolism, such as flavonoids, anthocyanins, and artemisinin (Innes et al., 2019; Thakur and Sharma, 2024).

## 6 Transcriptional regulation of major secondary metabolic pathways

Transcription factors (TFs) are regarded as the master regulators of plant metabolism. They are attached to the enhancer regions (cis-regulatory elements) in the promoters of their target genes. Depending on this, they either turn on or silence the expression of the specific gene (Shrestha et al., 2018). Among various TF families, the MYB, bHLH, WRKY, AP2/ERF, NAC, bZIP, TCP, and BBX families have been considered as main players in the regulation of secondary metabolism (Jan et al., 2021; Rabeh et al., 2025).

Recent research in genomics, transcriptomics, and functional genomics has dramatically expanded our knowledge of transcriptional regulation in secondary metabolism (Zhou et al., 2024). Together, genome assemblies with multi-omics analyses allowed the identification of regulatory networks that are involved in the control of the tissue-specific and stress-induced accumulation of metabolites. Transcriptomics and metabolomics in integration decoded the complex regulatory hierarchies and feedback mechanisms of plant metabolism.

Several TF families are involved in pathway-specific regulation. R2R3-MYB proteins act as important regulators in controlling the biosynthesis of flavonoid and phenylpropanoid pathways (Ma et al., 2018). In the biosynthesis of anthocyanins, MYB factors like PAP1, PAP2/MYB113, and MYB114 have been reported as strong inducers, while MYBL2 and CPC are repressors, thus allowing fine-tuning of the pathway flux. A comparable specificity is found in the biogenesis of glucosinolates; MYBs regulate genes that encode central enzymes for precursor generation (Xiao et al., 2021).

The bHLH family, including the MYC-type regulators, is another major regulatory network. MYC2 and its bHLH relatives are involved in combining jasmonate (JA) signals to induce the expression of terpenoid, sesquiterpene, nicotine, and alkaloid biosynthesis genes (Martin et al., 2021). In *Catharanthus roseus*, MYC2 acts as a regulator of AP2/ERF TFs in the JA signaling pathway that connects the JA perception with the activation of the terpenoid indole alkaloid (TIA) pathway (Table 3) (Pan et al., 2019).

**TABLE 3:** Major transcription factor families regulating the terpenoid pathway.

TF family	Reported size/abundance	Representative target genes/pathway nodes	Reported roles in abiotic stress	Key experimental evidence
AP2/ERF (DREB/ERF)	187 AP2/ERF-related transcripts identified in *W. somnifera*	HMGR, DXS, DXR, GGPPS, CAS (terpenoid backbone/sterol)	Induced by wounding, SA, MeJA, heat, and cold; links stress signals to terpenoid flux	WsAP2 overexpression upregulates terpenoid pathway genes; transcriptome mining and functional assays (Tripathi et al., 2020b)
WDR (WD40-repeat)	Most abundant TF family repertoire in leaf/root	Developmental regulators interfacing with secondary metabolism	Developmental integration: potential modulation of metabolite biosynthesis under stress	Overexpression of WsLWD1/WUSCHEL increased withanolide content (Tripathi et al., 2017)
WRKY	High abundance in the TF family repertoire in leaf/root	General phenylpropanoid/terpenoid regulation via JA/SA signaling	Canonical stress-responsive TFs; cross talk with JA/SA pathways	Transcriptomic prevalence; functional roles in regulation of withanolides (Tripathi et al., 2017)
MYB	Substantial representation across tissues	Phenylpropanoid/flavonoid; interaction with terpenoid genes	Light and stress-responsive regulation of secondary metabolism	Tissue-specific enrichment in berry transcriptome (Tripathi et al., 2020a)
bHLH	Present in TF repertoire	Often partners with MYB; affects terpenoid/phenylpropanoid branches	Integration of hormonal and stress cues	Transcriptomic presence; inferred roles from related species (Tripathi et al., 2017)
MADS/ELI (light-responsive)	Enriched in berry vs. leaf/root	Developmental and photomorphogenic control impacting downstream metabolism	Light/photoperiod coupling with stress responses; potential modulation of terpenoid flux	Differential TF distribution in berry transcriptome aligned with secondary metabolism (Tripathi et al., 2020a)

AP2/ERF family proteins are essential for JAs-responsive alkaloid biosynthesis (Tripathi et al., 2020a). ORCA1, ORCA2, and ORCA3 in *C. roseus* are rapidly upregulated by elicitors, and they induce early and late TIA pathway genes such as TDC and STR. Likewise, NIC2 locus ERFs are involved in tobacco nicotine synthesis together with bHLH proteins (Padmanabhan et al., 2025).

WRKY transcription factors mainly function in defense-related secondary metabolism. They function downstream of MAPK signaling cascades to induce biosynthesis of phytoalexins, such as camalexin and the specialized terpenoids, gossypol, and artemisinin. The ability of these sensors to couple stress sensing with metabolic productivity highlights their importance in inducible secondary metabolite pathways (Javed and Gao, 2023).

Other TF families, including NAC, DOF, SQUAMOSA promoter binding-like proteins (SPL), and bZIP proteins, participate in fine-tuning the pathway (Wang et al., 2025a; Bu et al., 2025; Xiong et al., 2025). ANAC042 controls camalexin production, OBP2 (DOF family) modulates the biosynthesis of glucosinolates, and SPL9 represses anthocyanin synthesis through its ability to disrupt complex formation by MYB–bHLH–WD40 (Qin et al., 2022). Alkaloid pathways are also regulated by bZIP proteins via transcriptional repression (Yamada and Sato, 2021).

### 6.1 Phenylpropanoid and flavonoid pathways

The phenylpropanoid pathway and its branch pathways involved in the production of flavonoids, anthocyanins, proanthocyanidins, and lignin are among the most well-known examples in which transcription regulation has been characterized in detail. Ma et al. (2018) established a full understanding of MYB regulation in the metabolism of phenylpropanoids, showing highly specific regulation of particular groups of MYB proteins to specific branches of the pathways (Ma et al., 2018). Flavonoid biosynthesis is regulated through control of the MBW complex, which is made up of R2R3-MYB, bHLH, and WD40 proteins (Xu et al., 2021; Zhang et al., 2019). The complex interacts and binds to the promoters of structural genes that encode enzymes like chalcone synthase (CHS), chalcone isomerase (CHI), flavanone 3-hydroxylase (F3H), and dihydroflavonol 4-reductase (DFR) (Kaur et al., 2020). The regulation of this MBW complex is variable and depends on developmental and environmental contexts (Xu et al., 2021). Specific combinations of MYB and bHLH regulate the late biosynthesis genes (LBGs) of the anthocyanin pathway, including dihydroflavonol 4-reductase (DFR), anthocyanidin synthase (ANS), and UDP-glucose:flavonoid 3-O-glucosyltransferase (UGFT) (Sharma et al., 2024). Reports showed anthocyanin pigment regulation in plants and introduced factors, such as light and temperature, regulate biosynthesis through transcription factors such as HY5 (Zhao et al., 2021).

In a recent study, the regulation of flavonoid biosynthesis has been viewed as a crucial means of unraveling plant adaptive mechanisms, pointing out that the patterns of flavonoid accumulation are a reflection of genetic programming and/or a response to the environment (Wang et al., 2025a). By integrating developmental and environmental information at the level of gene expression, it becomes possible for plants to optimize their flavonoid production (Wang et al., 2025a). Recent reports suggested the relevance of tissue-specific control of metabolic pathways by TFs. In *Quercus variabilis*, the activation of tannin production was reported by the activation of TCP3 TFs (Wang R. et al., 2024). This suggested that TFs can control metabolic pathways in a tissue-specific manner. Similarly, tissue-specific control of phenylphenalenone production by WRKY TFs was reported in *Musella lasiocarpa*, which showed that TFs have specialized control over metabolic pathways according to plant tissues (Huang et al., 2025).

### 6.2 Terpenoid biosynthesis

Terpenoids, also known as isoprenoids, are the largest class of plant secondary compounds. They have various roles in plants, ranging from stress regulation to defense (Xavier et al., 2023). The regulation of terpenoid biosynthesis by transcription has been well investigated in medicinal plants, where terpenoids are considered the major active compounds (Huang et al., 2025; Tholl et al., 2023). Chen et al. (2021) reported that the ERF TF PjERF1 directly binds to the promoters of central genes involved in the biosynthesis of triterpenoid saponins in *Panax japonicus* and controls the production of these compounds in the plant (Chen et al., 2021). This kind of regulation at multiple points ensures the simultaneous regulation of the expression of genes involved in the biosynthesis of saponins, such as β-amyrin synthase (βAS), cycloartenol synthase (CAS), and squalene epoxidase (SE), which stimulates the production of ginsenosides and saponins, and is an effective strategy in the regulation of a complex biosynthetic pathway (da Silva Magedans et al., 2021). A recent study emphasized the range of regulatory processes present among different terpenoids. The regulation of monoterpene biosynthesis pathways in grape plants, for example, is mediated by bHLH-type TFs to directly induce terpene synthase genes (Sun et al., 2025). Certain bHLH genes were later identified to express proportionally with monoterpene content in grape berries (Sun et al., 2025). *Salvia miltiorrhiza*, a medicinal herb known for tanshinone (diterpenoid quinone) production, revealed a complex regulation network orchestrated by several families of TF proteins, namely, MYB, bHLH, and WRKY, to control the expression levels of terpene synthases and cytochrome P450s, which participate in tanshinone biosynthesis pathways (Shao et al., 2025). Reviewing complex regulation patterns in secondary metabolism in medicinal plants, researchers explained the complex patterns in terpenoid metabolism regulation in medicinal plants (Wang et al., 2025a). Paclitaxel biosynthesis in *Taxus chinensis* is an example of extremely complex transcription regulation (Ren et al., 2025). A recent study reported that TFs are involved in paclitaxel biosynthesis, in which jasmonic acid-elicited responses trigger massive transcriptional reprogramming. This complexity reflects the nature of taxane biosynthesis, which involves enzymatic steps and cellular compartments (Song C. et al., 2022).

### 6.3 Alkaloid biosynthesis

Alkaloids are nitrogen-containing plant secondary metabolites. These compounds have various biological activities, ranging from drugs to plant defense compounds. Alkaloid biosynthesis is under transcriptional regulation and varies among plant groups (Faisal et al., 2024). Researchers studied the regulatory role of plant TFs in alkaloid biosynthesis (Godbole et al., 2022). The report emphasized the complexity of TF families involved in the process, which include MYB, bHLH, WRKY, AP2/ERF, and bZIP. Various alkaloid biosynthetic pathways follow different modes of regulation based on the individual evolution history and the resulting chemical properties (Yamada and Sato, 2021).

Tropane alkaloid biosynthesis, which has received intense scrutiny in Solanaceae species, is controlled by TFs and microRNAs. A recent study discussed TF and microRNA-mediated regulation of tropane alkaloid biosynthesis. This report makes it clear that both transcriptional and post-transcriptional regulation of tropane biosynthesis occur (Rabeh et al., 2025). TF-mediated regulation of biosynthesis is thought to play an important role in controlling tropane alkaloid biosynthesis. *Huperzia serrata* is a lycophyte that produces the acetylcholinesterase inhibitor huperzine A. A study suggested that 13 coordinately expressed TFs were key regulators in huperzine A biosynthesis. This case study demonstrates that, even though these organisms are non-flowering, there can be a complex transcriptional regulation of alkaloid biosynthesis in such organisms. These key regulators can be used to increase huperzine A production using a metabolic engineering strategy. The biosynthesis of isoflavones in *Trifolium pratense* (red clover) is controlled by transcriptional regulation that involves isoflavone synthase and other enzymes specific to the biosynthetic pathway (Cao et al., 2025).

### 6.4 TF complexes of higher orders and network combinations

Combinatorial interactions usually amplify the contribution of individual TFs. The MYB–bHLH–WD40 (MBW) complex is a typical case that was identified as a key regulatory module in the biosynthesis of flavonoid and anthocyanin (Qin et al., 2022). Its constitution defines tissue specificity of the pigmentation and developmental control, allowing for plastic pigmentation patterns to be set in motion under the influence of light, sucrose, or hormonal signals. In addition to transcriptional regulation, MBWs are also modulated by miRNAs, siRNA, and protein stability mediated through the ubiquitin–proteasome system (Liu et al., 2022).

Family-to-family interactions are also the basis of the alkaloid and terpenoid pathways. The bHLH-mediated cooperation with AP2/ERF TFs enhances the JA-responsive induction of TIA pathways, and the coordinated biosynthesis of phytoalexin is coordinately modulated by WRKY–MAPK modules during pathogen responses (Tripathi et al., 2017).

Secondary metabolites, usually induced under biotic or abiotic stress conditions, and TFs are key factors mediating these inducible metabolic responses (Tripathi et al., 2017). Stress-induced TFs induce defense pathways involved in the biosynthesis of secondary metabolites, including nicotine, camalexin, diterpenoid phytoalexins, and artemisinin. TF networks provide an adaptive interface that redirects metabolic flux toward defensive compounds by sensing environmental stress signals and transducing them into biochemical defense outputs.

### 6.5 Hormonal cross talk and transcription factor activation

Hormones such as abscisic acid (ABA), jasmonic acid (JA), salicylic acid (SA), ethylene (ET), auxin, and gibberellins are pivotal in transmitting the stress-induced responses by integrating various stress signals through controlling the expression of specific transcription factors (Singh and Roychoudhury, 2023). Several TF families are specifically engaged in this regulation: MYB regulates sesquiterpene biosynthesis and drought response; WRKY controls defense-related and secondary metabolism genes; bZIP participates in activation of ABA-responsive genes, ABRE-binding factors (ABFs); ERF/AP2 is a player of ethylene signaling for stress acclimation; NAC coordinates JA- and SA-mediated pathway often induced by drought. TFs bind to the promoter regions of terpenoid biosynthesis genes, including terpene synthases (TPSs), geranyl diphosphate synthase (GPPS), farnesyl pyrophosphate synthase (FPPS), and geranylgeranyl diphosphate synthase (GGPPS), affecting the transcription process and modulating metabolites in stress response as well as metabolism (Srivastava et al., 2022).

## 7 Nanotechnology interfaces with metabolomics: an integrated framework

### 7.1 Nanomaterials as enablers of advanced metabolomics

Nanotechnology is changing metabolomics by detecting metabolites in a very sensitive, spatially resolved, and real-time manner, thus changing our knowledge about cellular metabolism under normal and stressful conditions (Lin et al., 2025; Majumdar and Keller, 2021). The center of this collaboration is the application of engineered nanomaterials like gold and silver NPs, quantum dots, carbon nanotubes, graphene, and metal–organic frameworks (MOFs), in metabolomics platforms (Lin et al., 2025). Metabolites have high surface-area-to-volume ratios and tunable surface chemistry; their plasmonic effects and excellent electrical conductivity characteristics enhance their capture, separation, and signal transduction (Cho et al., 2025). Thus, the existing workflows in metabolomics gain much in terms of analytical sensitivity, selectivity, and throughput.

### 7.2 Nanostructured enhancements in mass spectrometry-based metabolomics

In mass spectrometry-based metabolomics, nanostructured matrices, and particularly the ones used in nanoparticle-assisted laser desorption/ionization (nano-LDI), have played an important role in improving analysis performance (Wang D. et al., 2024). The nano-LDI matrices not only provide a significant reduction of chemical background noise compared to traditional organic matrices but also yield better ionization efficiency. They make the sensitive and reproducible detection of low-molecular-weight and low-abundance metabolites, which include sugars, organic acids, amino acids, lipids, and phytohormones, possible (Jorge et al., 2016). These metabolites are often difficult to study using the conventional MALDI methods (Ponce-Rodríguez et al., 2020). In addition, the use of nanostructured coatings and nanoporous materials for the chromatographic columns and solid-phase extraction systems has resulted in enhancing the metabolite enrichment, decreasing the sample complexity, and elevating the analytical depth and reproducibility across the whole metabolomic datasets (Souza and Fernie, 2024).

### 7.3 Nanoengineered microfluidics and single-cell metabolomics

Nanotechnology has led to revolutionary changes in microfluidics and lab-on-a-chip-based metabolomics. Nanofabricated microfluidic platforms conduct rapid, fully automated sample processing with hardly any or unnoticeable metabolite degradation or loss, all the while working with volumes as small as nanoliters and picoliters. These devices provide high precision in fluidic control, low reagent consumption, and fast processing; thus, they are especially appropriate for time-resolved and large-scale metabolomic studies. If connected with mass spectrometry or optical detection systems, microfluidic units can carry out metabolic profiling on extremely small biological samples such as rare cell types, chosen areas within tissues, and even individual cells (Kumar and Katiyar, 2025). This fusion has played a key role in revealing the metabolic diversity of cells and the metabolism-related reactions that are lost in large-scale and bulk metabolomics techniques.

### 7.4 Nanobiosensors for real-time and *in vivo* metabolite monitoring

Nanobiosensors connect the fields of nanotechnology and metabolomics, facilitating the instant and continuous observation of metabolic processes not only during the normal functioning of organisms but also under stress conditions. The working of these sensors is based on the application of functionalized NPs like gold and silver NPs, quantum dots, carbon nanotubes, graphene-based materials, and metal oxide NPs, which are specially designed to selectively find, join, and capture certain metabolites (Silva et al., 2023). By changing the surface with enzymes, antibodies, aptamers, or molecularly imprinted polymers, the nanobiosensors attain outstanding specificity and sensitivity for the detection of key metabolic compounds, including sugars, amino acids, phytohormones, reactive oxygen species (ROS), and a variety of secondary metabolites (Sharma and Dhadly, 2023).

A key advantage of biosensors that utilize NPs is their capability to manufacture and change their optical, electrochemical, and physicochemical properties very finely. For example, the binding of a metabolite leads to changes in fluorescence, surface plasmon resonance, electrical conductivity, or redox signals, which result in rapid signal transduction with a low level of interference from the biological matrices (Naresh and Lee, 2021). The whole process results in very fast response times and high temporal resolution, which means that the transient metabolic fluctuations and dynamic metabolic fluxes that are usually missed by the conventional end-point metabolomics approaches can now be captured.

Crucially, the aforementioned nanosensors can be utilized *in vivo*, allowing for the monitoring of metabolic processes in plants, microbes, and animal systems to be done non-destructively and be conducted with high spatial resolution (Wurtzel and Kutchan, 2016). To illustrate, in the field of plant biology, nanobiosensors have been employed to track the occurrences of ROS signaling, sugar movement, and hormone distribution changes in real time in various conditions such as development and abiotic stress (Butnariu and Butu, 2019). Likewise, in both microbial and animal systems, sensors based on NPs grant knowledge of metabolic control, cell communication, and stress resistance, among other things. All in all, nanobiosensors link the static metabolite profiling to the dynamic metabolic regulation, thereby making a considerable contribution to the functional interpretation of the underlying data from the metabolomics process.

## 8 Abiotic stress mitigation through the application of nanoparticles

Nanoparticles (NPs) have become key players in protecting agricultural production from various abiotic stresses, including salinity, drought, heat, and heavy metals. They exert their influence mainly through modulating photosynthetic efficiency, redox homeostasis, and nutrient acquisition processes. Contemporary reviews and empirical studies vouch for the ability of the metal and metal oxide NPs, and NP–hormone synergies, to significantly increase the tolerance of stress in a wide range of crops (Singh et al., 2021) (Table 4).

**TABLE 4:** Abiotic stress mitigation by different nanoparticle strategies.

Abiotic stress	Monometallic NP	Bimetallic/advanced NP	NP + phytohormone	References
Drought	ZnO, TiO_2_, Fe_3_O_4_, and SiO_2_ NPs improve germination, root growth, RWC, photosynthesis, antioxidants, and osmolytes in cereals and legumes	Bimetallic/engineered NPs proposed to enhance ROS control and water-use efficiency	NPs + ABA/melatonin/SA/NO donors strongly improve water status, antioxidant system, and drought-responsive genes vs. single treatments	Khan et al. (2024); Dilnawaz et al. (2023); Al-Khayri et al. (2023); Alabdallah et al. (2021)
Salinity	ZnO, SiO_2_, CuO, and Se NPs reduce Na^+^ uptake, improve K^+^/Na^+^ ratio, chlorophyll, antioxidants, and SOS/ABA responses	Bimetallic NPs are suggested to boost ion homeostasis and redox buffering under salt stress	Co-application of NPs with cytokinins, GA, SA, and PGPR-derived signals enhances ion balance, osmolytes, and photosynthesis under salinity	Khan et al. (2024), Rajput et al. (2021); Dilnawaz et al. (2023)
Heavy metals (Cd, Pb, etc.)	SiO_2_, Fe_3_O_4_, ZnO, and Cu-NPs limit metal uptake/translocation, reduce ROS/MDA and enhance antioxidants and phytohormone balance	Bimetallic NPs proposed for stronger metal immobilization and ROS quenching	Melatonin + SiO_2_ NPs in Cd-stressed rice: higher growth, photosynthesis, antioxidant enzymes, lower ROS and lipid peroxidation; similar concepts with SA/JA/ethylene modulators	Khalid et al. (2025), Kaleem et al. (2024), Faisal et al. (2024), Rajput et al. (2021), Dilnawaz et al. (2023), El-Nasr et al. (2025)
Temperature extremes	ZnO, Ag, and Si-based NPs stabilize membranes, pigments, and antioxidants under heat/cold stress	Not well explored for temperature stress yet	NPs interacting with ABA, JA, and SA help regulate heat/cold-responsive signaling and ROS–hormone cross talk	Khalid et al. (2025), Rajput et al. (2021), Thabet and Alqudah (2024), El-Saadony et al. (2022), Al-Khayri et al. (2023)

## 8.1 Monometallic NPs and abiotic stress tolerance

Notably, the NPs of ZnO, TiO_2_, Fe_3_O_4_/Fe_2_O_3_, SiO_2_, Ag, CuO, and Au have been empirically shown to improve vegetative growth, biomass accumulation, chlorophyll concentration, photosynthetic performance, and activity of enzymes such as superoxide dismutase (SOD), catalase (CAT), ascorbate peroxidase (APX), and peroxidase (POD), which are important in antioxidant defense. These benefits have been attributed to the mitigation of reactive oxygen species (ROS), a reduction of H_2_O_2_ and malondialdehyde (MDA) levels, improved K^+^/Na^+^ ratios, and an enhancement of water status (El-Saadony et al., 2022; Sen et al., 2025; Ghani et al., 2021; Wang L. et al., 2022). While low to moderate concentrations of NPs offer beneficial results, increased dosages, especially of ZnO and CuO, can induce oxidative stress and inhibit plant growth (Shaghaleh et al., 2025; Alabdallah et al., 2021; Al-Khayri et al., 2023) (Table 4).

### 8.2 Bimetallic nanoparticles and increased mitigation

Bimetallic formulations like Ag/ZnO or Ni-Au tend to have better surface reactivity and catalytic proficiency than single-metal systems, leading to better redox regulation and electron transfer processes. Consequently, these tend to be superior to their monometallic counterparts in antioxidant activity, photocatalytic activity, and antimicrobial activity, highlighting the significance of their possible applicability in multi-stress plant situations (Dubey et al., 2024; Pavel et al., 2025; Zhang L. et al., 2025; Afzal et al., 2023).

### 8.3 Nanoparticles as an additive to phytohormones

Co-application of NPs with phytohormones (salicylic acid (SA), abscisic acid (ABA), jasmonic acid (JA), gibberellins (GA), and cytokinins) or alternative mitigating agents (plant growth-promoting rhizobacteria (PGPR), fungus, metal salts, and biochar) exhibits synergistic effects. These include increased antioxidant capacity, accumulation of osmolytes, better nutrient uptake, water-use efficiency, and the overexpression of stress-responsive genes, which outperform the individual responses to either intervention (Khan et al., 2024; Rehman et al., 2024).

#### 8.3.1 Genomic insights into terpenoid (withanolide) biosynthesis under abiotic stress

Plants respond to environmental extremities comprising abiotic stresses (e.g., drought, salinity, and temperature extremes) by controlling primary and secondary metabolism, usually by redirecting fluxes via terpenoid, phenylpropanoid, and flavonoid pathways (Sangwan et al., 2013). In medicinal/stress-resilient plants such as *Withania somnifera*, the steroidal lactones (withanolides) are synthesized through the terpenoid backbone synthesis (*via* the mevalonate [MVA] and 2-C-methyl-D-erythritol-4-phosphate [MEP] pathways) and downstream cyclization and functionalization steps (Sangwan et al., 2008; Srivastava and Sangwan, 2020). The genomic and transcriptomic surveys could provide key insights into how this biosynthesis is adapted under stress.

In a berry transcriptome study of *W. somnifera,* we studied a high-depth dataset of berry tissue (alongside leaf and root) and found almost all genes associated with withanolide biosynthesis. It was suggested that while the terpenoid/sterol backbone genes had broadly similar transcript abundances across tissues, the berry transcriptome was enriched in transcripts linked to steroid, phenylpropanoid, and flavonoid metabolism, as well as in cytochrome P450s, methyltransferases, and glycosyltransferases, which likely drive chemical diversification (Tripathi et al., 2020a). Importantly, the investigation identified distinct tissue-specific transcription factor (TF) distributions (e.g., MYB, ELI (early light-inducible protein), MADS, and WRKY) associated with developmental and secondary metabolism regulation (Tripathi et al., 2020a).

In the light of abiotic stress, the conjunction of developmental signals, light/photoperiod responses, and stress signaling can affect terpenoid metabolism. For example, the alignment of ELI (light-responsive) and related TF families against secondary metabolism-associated genes in berry speculates a coupling of developmental/photomorphogenic control with metabolite synthesis. During stress, plants often induce isoprenoid flux to strengthen defense, membrane integrity, or antioxidant capacity; therefore, the observed TF metabolite gene network in *W. somnifera* provides a genomic basis for stress adaptation via terpenoid metabolism.

Our study, comprising *in silico* mining and functional characterization of the AP2/ERF TF super-family in *W. somnifera*, provides a direct link between transcriptional regulation, terpenoid biosynthesis, and stress responses (Tripathi et al., 2020b). In this study, we identified 187 AP2/ERF gene-related transcripts (GRTs) in the transcriptome of *W. somnifera*, classified them into sub-families (AP2, ERF, DREB, etc.), and conducted cloning experiments followed by transient overexpression of one representative member (*WsAP2/ERF*) in leaf explants. The overexpression of *WsAP2* led to enhanced expression of key terpenoid pathway genes such as HMGR (3-hydroxy-3-methylglutaryl-CoA reductase), DXS (1-deoxy-D-xylulose-5-phosphate synthase), DXR (1-deoxy-D-xylulose-5-phosphate reductoisomerase), CAS (cycloartenol synthase), and GGPPS (geranylgeranyl diphosphate synthase) (Tripathi et al., 2020b). We further proposed that WsAP2 may directly or indirectly regulate the GGPPS gene, thereby channeling the flux toward the terpenoid backbone, probably increasing the accumulation of withanolides during stress conditions.

Taken together, these transcriptomic studies of *W. somnifera* presented important lines of evidence: first, for the terpenoid/withanolide pathway components that are broadly expressed but show tissue-specific modulation; second, TF families (WRKY, MYB, AP2/ERF, and WDR) are abundantly present and likely serve as nodes linking developmental/stress signals to metabolic gene expression, and lastly, the overexpression of specific TFs (such as WsAP2) can upregulate pathway genes, predicting a regulatory route that could be potentially exploited to enhance metabolite accumulation or stress resilience. In the view of abiotic stress adaptation, the increased flux through terpenoid/sterol pathways may provide protective functions in the form of membrane stability, antioxidation, and signaling. The TF metabolite gene analysis offers a mechanistic basis for such adaptation.

#### 8.3.2 Terpenoid pathway gene regulation

As demonstrated in the AP2/ERF study, the overexpression of WsAP2 visibly upregulated genes such as HMGR, DXS, DXR, CAS, and GGPPS. These are the expected key nodes in MVA/MEP/sterol pathways, which channelize toward withanolide biosynthesis. Therefore, TFs might shift the metabolic flux toward terpenoid skeleton production, possibly enhancing specialized metabolite accumulation (Tripathi et al., 2020b).

#### 8.3.3 Tissue and developmental specificity

The study related to the berry transcriptome reported that TFs such as ELI, MYB, MADS, and WRKY are expressed differentially across various tissue transcriptomes, such as berry, leaf, and root, and likely are under developmental regulation (Tripathi et al., 2020a). Because terpenoid metabolites usually accumulate in specific tissues or different developmental stages, e.g., fruit, berry, root, etc., the combinatorial effect of TF networks suggests spatiotemporal fine-tuning of pathway gene expression (Tripathi et al., 2020a).

##### 8.3.3.1 Cross talk with stress signaling

The stress-oriented accumulation of terpenoids and other secondary metabolites is well recognized. TFs such as AP2/ERF and WRKY relate stress signaling (e.g., via JA/SA/ethylene) to the activation of biosynthetic pathways. As in the case of *W. somnifera*, WsAP2 is induced by abiotic stress, and its overexpression enhances terpenoid pathway gene expression, suggesting that plants may direct such TF metabolite modules as part of the adaptive response (Tripathi et al., 2020b).

From a genomics perspective, this implies that the regulatory pattern for terpenoid metabolism is expected to possess both constitutive developmental TFs (WDR, MYB, bHLH, and MADS) and stress-responsive TFs (AP2/ERF and WRKY). In combination, these facilitate plastic adaptation, that is, under normal growth, they maintain baseline terpenoid synthesis, whereas under abiotic stress, they shoot up flux via induced TFs, thus reinforcing secondary metabolite accumulation and thereby contributing to stress adaptation (Tripathi et al., 2020a; b).

## 9 Tools and techniques of metabolomics

### 9.1 Preparation of samples

In metabolomics, plant sample preparation involves collecting the sample. After that, metabolites are extracted and measured. Various steps are involved in sample preparation, like harvesting, quenching, extraction, and derivatization, as shown in Figure 3.

### 9.2 Key analytical tools in agricultural metabolomics

There are two types of metabolomics analysis, targeted and non-targeted. Non-targeted analysis involves the separation of many metabolites on pattern-based global and unbiased screening (Saini et al., 2024; Naz et al., 2014). It is used more frequently to answer the discovery-based queries, such as characterization of the most significant metabolic alterations under stress conditions. The advantages of non-targeted metabolomics include high unbiased coverage; however, it has difficulties with relative quantification, complex data processing, and unknown identification. In contrast, the aim of targeted metabolomics is the identification, quantification, and interpretation of specific responses. Targeted metabolomics focuses only on known metabolites and can provide absolute quantification. Metabolite analysis could be carried out based on mass spectrometry (MS), which entails the identification and measurement of plant secondary compounds in a sample. Time-of-flight (TOF) and quadruple or ion trap analyzers may be used in a precise manner to obtain information on the compound nature formed via ionization using positive and negative ion modes (Isah, 2019). Nuclear magnetic resonance (NMR) is commonly used for the identification of metabolites. One benefit of NMR is that it does not require sample separation, allowing samples to be retrieved for further study (Powers et al., 2024). Most MS methods are, however, intrinsically more sensitive than NMR; it can be challenging to analyze a complex mixture of NMR spectra. Gas chromatography-mass spectrometry (GC-MS) allows the identification of metabolites without the need for standard compounds. While GC-MS is recognized for its high capacity, excellent retention time repeatability, and readily available compound libraries, it does necessitate that the samples be heated until they become gaseous. As a result, GC-MS is often employed to identify primary metabolites and volatile compounds. In contrast, liquid chromatography-mass spectrometry (LC-MS) can detect a wide range of metabolites. In LC-MS, the chemical derivatization of nonvolatile metabolites is not required (Aszyk et al., 2018). The development of ultra-performance liquid chromatography (UPLC) significantly boosted the speed and sensitivity of detection. Therefore, techniques like GC-MS, UPLC, and LC-MS are widely utilized in metabolomics studies. Additionally, metabolomics studies frequently involve the use of matrix-assisted laser desorption/ionization-mass spectrometry (MALDI-MS), direct infusion-mass spectrometry (DI-MS), and capillary electrophoresis-mass spectrometry (CS-MS) due to their different functions (Ren et al., 2018) (Table 5). Due to the complex nature of metabolites, it is essential to select the most suitable detection techniques based on cost, sensitivity, throughput, and resolution (Feng et al., 2020).

**TABLE 5:** Different techniques in plant metabolite identification under stress conditions.

S. No.	Technique	Principle	Plant	Application	Advantage	Limitation	References
1	Gas chromatography-mass spectrometry (GC-MS)	Separates metabolites based on volatility and mass-to-charge ratio (m/z)	*Arabidopsis thaliana* *Triticum aestivum* (wheat) *Hordeum vulgare* *Cucumis sativus* *Helianthus annuus* *Glycine max* (soybean)	Primary metabolites like sugars, amino acids, and organic acids are affected by drought, salt, and temperature stress	High sensitivity, well-established libraries (NIST and FiehnLib)	Requires derivatization for nonvolatile metabolites and time-consuming	Baek et al. (2021); Ammar et al. (2023); Derakhshani et al. (2020); Andrade et al. (2021); Nam et al. (2019)
2	Liquid chromatography-mass spectrometry (LC-MS)	Separates metabolites in the liquid phase, coupled with MS for identification	*Oryza sativa* (rice) *Solanum lycopersicum* (tomato)	Used for secondary metabolites (flavonoids, phenolics, and alkaloids) in response to heavy metal, drought, and UV stress	High sensitivity, covers a broad range of metabolites	Complex data analysis and variable ionization efficiency	Zhao Y. et al. (2024); Sudiro et al. (2022)
3	Nuclear magnetic resonance (NMR) spectroscopy	Identifies metabolites based on the magnetic properties of nuclei	*Glycine max* (soybean) *Brassica napus* (rapeseed) *Medicago sativa* (alfalfa)	Used for metabolic fingerprinting of plants under salinity, drought, and oxidative stress	Non-destructive, quantitative, and requires minimal sample preparation	Lower sensitivity than MS-based methods and expensive equipment	Msomi et al. (2024); Raza et al. (2023)
4	Fourier transform infrared spectroscopy (FTIR)	Measures infrared absorption to identify functional groups of metabolites	*Sesuvium portulacastrum* (sea purslane)	Used for analyzing lipid and carbohydrate composition under drought and salinity stress	Rapid, non-destructive, and minimal sample preparation	Limited metabolite specificity and overlapping peaks	Nikalje et al. (2019)
5	Capillary electrophoresis-mass spectrometry (CE-MS)	Identifies metabolites based on the charge-to-size ratio; metabolites are separated under an electric field, detected via mass spectrometry	*Arabidopsis thaliana* (*Arabidopsis*)	Analysis of amino acids and organic acids under stress	High sensitivity and suitable for ionic and polar metabolites	Limited to polar metabolites and complex sample preparation	Ohnishi et al. (2018)
6	Gas chromatography-time-of-flight mass spectrometry (GC-TOF-MS)	Measures the time it takes for ions to reach the detector, allowing for high-throughput metabolite identification	*Hordeum vulgare* (barley)	Identifies unknown metabolites and secondary metabolites under stress	High mass accuracy, broad metabolite coverage, and detection of unknown compounds	Expensive instrumentation and requires advanced data analysis	Chmielewska et al. (2016)

### 9.3 Data analysis and interpretation in metabolomics

Analyzing and interpreting the huge amounts of information produced by metabolomics data is complex and necessitates the use of advanced statistical methods and computational tools. The data analysis procedure consists of several essential steps.

#### 9.3.1 Data pre-processing

Data received from instruments, such as NMR and MS, undergo processing to remove noise and correct the differences between samples. The step involves peak detection, alignment, and normalization to ensure accurate comparison (Amberg et al., 2017). NMR-based metabolomics analysis is a non-destructive method that effectively reveals the structural information of metabolites (Deborde et al., 2017). MS is often paired with chromatographic separation techniques, like LC-MS and GC-MS. In order to quantify metabolites directly without requiring prior chromatographic separation, the direct injection mass spectrometry (DI-MS) technique has been upgraded to involve rapid, high-throughput fingerprinting techniques with the use of high-resolution mass spectrometers like Fourier transform ion cyclotron resonance (FT-ICR). This method enables the separation of complex biological samples comprising a mixture of metabolites before ion detection, which aids in differentiating isobaric compounds with similar masses.

#### 9.3.2 Multivariate analysis

Univariate, multivariate, supervised, and unsupervised analyses can be employed to perform various statistical techniques (Blaise et al., 2021). Univariate statistics are used in the targeted metabolomics to evaluate hypotheses related to each metabolite of interest and determine their significance (Xu et al., 2024). In contrast, multivariate statistics are often used in the untargeted metabolomics to analyze a global matrix dataset (Alonso et al., 2015). This includes unsupervised techniques like principal component analysis (PCA), along with supervised multivariate techniques like partial least squares-discriminant analysis (PLS-DA), and orthogonal projections to latent structures discriminant analysis (OPLS-DA). Unsupervised methods are useful for sample clustering, which helps to identify trends, outliers, and groups of observations. The identification of potential biomarkers in OPLS-DA is often carried out using variable importance in projection (VIP) scores (Table 5). After processing, the spectrum databases can be employed to identify interesting substances, which can then be confirmed using reference standards.

#### 9.3.3 Targeted metabolomics

In wheat, fatty acid abundance plays a significant role in drought stress tolerance, which was identified through targeted metabolomics (Ullah et al., 2022). Similarly, a number of metabolites and the differences among them were analyzed by using target metabolomics analysis in licorice (Zhang et al., 2023). Zhang et al. (2021) observed that some metabolic changes take place in two cotton varieties during drought stress by using a targeted metabolomics approach. In broomcorn millet grains, a widely targeted metabolomics analysis revealed the impact of alkaline stress on metabolites (Ma et al., 2023). A study of the metabolic profile of chestnut (*Castanea mollissima*) using targeted metabolomics provided a new understanding of the chestnut calcification process (Xiao et al., 2021). Targeted metabolomics was used to determine the mechanism of flue-cured tobacco quality improvement (Meng et al., 2022). In maize, tryptophan enhances drought stress tolerance through IAA signaling, and SA and nicotinamide adenine dinucleotide (NAD) subsequently scavenge ROS. Three key genes involved in the production of tryptophan are flavin-containing monooxygenase 6 (ZmYUC6), catalase 1 (ZmCAT1), and indole-3-acetaldehyde oxidase 1 (ZmAO1). The high expression of these genes has a major impact on the regulation of drought tolerance (Li S. et al., 2024). In *Pinus taeda* seedling roots, the amino acids glycine and asparagine and the phytohormone gibberelline (GA4) were found, while in needles, the phytohormones abscisic acid and salicylic acid were significantly increased (Wu et al., 2023). Similarly, under molybdenum (MO), the hormone salicylic acids, jasmonic acids, and methyl jasmonate, amino acids like 2-oxoarginine and l-phenylalanine, and lipids like lysophosphatidylcholine, monoglyceride, phosphatidyl glycerol, and diacylglycerol were increased (Feng et al., 2020).

#### 9.3.4 Untargeted metabolomics

Untargeted metabolomics was used to analyze the variations in the metabolites of two genotypes of *Glycyrrhiza uralensis* under drought stress (Zhang et al., 2022a). In *P. taeda* seedlings, untargeted metabolomics found that sugars, flavonoids, long-chain lipids, and terpenoids are increased under drought stress (Wu et al., 2023). Under salinity stress conditions, metabolic pathways and salt-responsive metabolites in *Haloxylon salicornicum* were analyzed by a non-targeted metabolomics approach (Panda et al., 2021). Under drought stress, 523, 406, 301, and 272 metabolites were recognized using untargeted metabolomics analysis in two genotypes of quinoa (Zhu et al., 2023). Similarly, in *Quercus ilex* L. leaf seedlings, a number of putative compounds, which are related to plant tolerance under drought stress, were identified through the use of untargeted MS-based metabolomics analysis (Tienda-Parrilla et al., 2022). In *Aphanamixis polystachya* seedlings, the regulatory pathways were determined by using untargeted metabolomics in response to salicylic acid-mediated stress conditions. A study using LC-MS-based untargeted metabolomics analysis found that bioactive compounds of *Punica granatum* juice increased in drought stress (Gómez-Bellot et al., 2023). In cotyledons and roots of *Ricinus communis* seedlings, untargeted LC-MS-based metabolomics analysis identified metabolic changes under salinity stress (Wang et al., 2021). In the edible and medicinal plant *Saliva miltiorrhiza*, untargeted LC-MS/MS-based metabolomics profiling was done to analyze a number of metabolites under cadmium stress (Yuan et al., 2022). In *Pinus pinaster*, the essential biochemical rearrangement was identified by using untargeted metabolomics under heat and drought stress (López-Hidalgo et al., 2023). The adaptation response of *Gliricidia sepium* was shown under salinity stress by using untargeted metabolomics analysis (Braga et al., 2022).

##### 9.3.4.1 Single-cell and spatial metabolomics for tissue-level resolution of stress responses

The research on the effect of abiotic stress on plants has been significantly and positively impacted by single-cell and spatial metabolomics, which have affected the metabolic reprogramming of the plant tissues to the specific resolution of the cell types, thus performing bulk metabolomics averaging. The single-cell metabolomics studies that have been done using *A. thaliana* have shown the specific accumulation of proline, malate, and soluble sugars in the guard cells under drought stress conditions, thereby establishing a direct link between the dynamics of osmolytes and stomatal regulation as well as water-use efficiency (Katam et al., 2022). In the same way, cell-resolved metabolomics in rice and maize roots has shown spatially distinct accumulation of amino acids, organic acids, and compatible solutes between epidermal, cortical, and vascular tissues under salinity stress, thus pointing out coordinated and at the same time heterogeneous metabolic adaptation (Deng et al., 2020).

NPs such as fluorescent quantum dots, plasmonic gold nanoparticles, and carbon-based materials, have been used successfully in plant metabolomics to visualize the stress-induced changes in redox metabolites, gradients of hormones (like abscisic acid and auxins), and ROS signaling at the tissue and cellular levels (Song Y. et al., 2022). These methods reveal the metabolic reprogramming during abiotic stress conditions, such as drought, salinity, temperature extremes, and heavy metal exposure, spatially resolved in a way that has never been seen before. Nanotechnology-enabled metabolomics brings together the physiological outcomes with the nanoscale metabolic signals, thus bridging the gap between molecular metabolism, stress adaptation, and phenotype expression (Zainal-Abidin et al., 2024).

Mass spectrometry imaging techniques like MALDI-MSI and DESI-MSI have boosted the power of spatial metabolomics and provided an opportunity to see the gradients of metabolites caused by stress throughout the tissue (Ma and Fernández, 2024). For instance, in drought and heat stress studies, it was confirmed that the epidermis of the leaves was the main site for the accumulation of flavonoids and phenolic compounds, which in turn, led to ROS scavenging and photoprotection (Zhang et al., 2022b). In a significant development, the technique of spatial lipidomics has uncovered the localized transformation of chloroplast galactolipids, that is, MGDG and DGDG, in the mesophyll tissues, which played the role of stabilizing the thylakoid membranes and maintaining the photosynthetic efficiency during drought and salinity stress (Hölzl and Dörmann, 2019). When considering this from the applied point of view, the merging of single-cell and spatial metabolomics with transcriptomics and phenotyping provides a very high opportunity for spotting tissue-specific metabolic hubs, hence speeding precision breeding as well as the targeting of metabolic engineering for resistance against stress in crops (Feussner and Polle, 2015; Zhang S. et al., 2025).

##### 9.3.4.2 Stable isotope tracing and fluxomics for resolving pathway directionality under abiotic stress

Stable isotope tracing together with metabolic flux analysis (fluxomics) is an effective method of identifying the direction of the pathway and measuring metabolic reprogramming in plants under abiotic stress (Koley et al., 2025). In contrast, steady-state metabolomics, which shows the sizes of metabolite pools by labeling isotopes with ^13^C, ^15^N, or ^2^H tracers, monitors the flow of carbon and nitrogen through interconnected metabolic networks and reveals the exact conditions under which pathways are activated, suppressed, or rerouted under stress (Srivastava et al., 2016).

For instance, research using ^13^CO_2_ and ^13^C-glucose labeling in *Arabidopsis* and some crops verified the rerouting of carbon from the Calvin–Benson cycle to glycolysis, the oxidative pentose phosphate pathway (OPPP), and amino acid production during drought and salt stress thus, leading to increased NADPH generation for redox equilibrium (Jang et al., 2018; Alseekh et al., 2018). Likewise, the use of ^15^N-labeling has determined the path of nitrogen movement toward proline, glutamate, and polyamine production during osmotic stress, putting an end to the argument of whether proline increase means active synthesis or reduces respiration (Limami et al., 2023). Flow studies have additionally brought to light the stress-related alterations at the TCA cycle branch points, among which the increased flow into the organic acid pools that are connected with ion chelation and pH regulation during the stress of heavy metals and salt.

Using isotope tracing in combination with spatial or cell-type-specific sampling has made it possible to analyze tissue-resolved fluxes, thereby exposing different metabolic patterns in roots and shoots or in mesophyll and vascular tissues (Jang et al., 2018). The detection of stress-adaptive metabolic bottlenecks through fluxomics is highly advantageous as it gives a logical foundation for the usage of metabolic engineering and breeding. These kinds of approaches will both increase the factor of a plant’s stress resistance and maintain the metabolic costs at a low level (Koley et al., 2024).

##### 9.3.4.3 AI-driven multi-omics integration for predictive modeling of abiotic stress responses

The rapid accumulation of vast omics datasets in multiple dimensions has considerably changed the area of plant stress biology; nevertheless, obtaining predictive and mechanistic insight from these layers of complicated data is still a very difficult challenge (Roychowdhury et al., 2023). By identifying nonlinear, hierarchical, and context-dependent interactions between the various omics categories, including genomics, transcriptomics, proteomics, metabolomics, lipidomics, and fluxomics, the application of artificial intelligence (AI) in multi-omics integration has been a groundbreaking technique in the modeling of plant responses to abiotic stress (Wu and Xie, 2025). Unlike traditional statistical techniques, AI frameworks may simultaneously incorporate many data types and environmental elements, allowing for the prediction of phenotypic outcomes in dynamic stress settings (Walsh et al., 2024).

Supervised machine learning algorithms, including random forests, support vector machines, and gradient boosting, have been extensively utilized for stress-responsive biomarker identification and for predicting tolerance traits (Gou et al., 2024). One remarkable example is the integrative models that combined genomic markers with transcriptomic and metabolomic features, which led to a substantial increase in the accuracy of drought and salinity tolerance predictions in rice, wheat, and chickpea when compared to single-omics genomic prediction models (Washburn et al., 2019; Westhues et al., 2021; Souza and Fernie, 2024). These methods have recognized the existence of key metabolic signatures like osmolytes, antioxidants, and lipid species and proved them to be secure predictors of stress performance.

In the past few years, deep learning architectures have made it possible to perform network-level inference and mechanistic modeling simultaneously. Dimensionality reduction and extraction of features from large-scale multi-omics datasets have been accomplished via autoencoders and variational autoencoders, leading to the discovery of underlying stress-responsive modules. In addition, the use of graph neural networks (GNNs) and attention-based models has further contributed to the reconstruction of regulatory and metabolic interaction networks by identifying gene–protein–metabolite relationships explicitly (Vrahatis et al., 2024). A case in point, GNN-based multi-omics integration has been successfully utilized to forecast the plant’s response to abiotic stresses and the corresponding regulatory modules and miRNAs, marking the crucial points of control in signaling networks during stress (Subramanian et al., 2020).

In the pursuit of more precise forecasts, it is noteworthy that AI-driven methods are increasingly being combined with spatial metabolomics and fluxomics. Models can infer the directionality of pathways, the presence of metabolic bottlenecks, and the trade-offs between growth and stress defense by combining isotope-based flux data with the layers of metabolomics and transcriptomics. Compared to using metabolite abundance alone, this type of modeling has made it possible to predict tolerance outcomes more accurately by revealing the diversion of carbon and nitrogen fluxes caused by stress toward redox maintenance and osmoprotection (Arrivault et al., 2019; Pérez-de-Souza et al., 2020).

In practical terms, the AI-powered integration of all omics offers a robust and comprehensive platform for mapping genotype to phenotype, conducting *in silico* stress studies, and choosing the optimal genes or pathways for genome editing and breeding (Gou et al., 2024). Predictive models can, in fact, lead to precise gene editing, climate-resilient crop design, metabolomics-assisted breeding, and more by integrating omics features with phenotypic and environmental data (Zenda et al., 2021). The strength of AI in predictive modeling will likely be the primary driver behind a new era of plant stress biology and the sustainable agriculture industry, with the development of explainable AI techniques and the standardization of multi-omics pipelines (Harfouche et al., 2019).

### 9.4 Analysis of metabolic pathways

Advanced bioinformatics tools are used to align metabolite data with metabolic pathways, offering a functional understanding of the metabolic changes observed (Cambiaghi et al., 2017). This analysis can reveal pathways that are either upregulated or disrupted, which are linked to particular conditions such as environmental stresses. Therefore, molecular networking, pathway analysis, biological modeling, and correction-based analysis can be used to link the biological interruption of metabolites identified by a metabolic network. In recent years, metabolomics and its integration with other omics techniques, such as viewers, route database, and molecular networking tools like BioCyc, GNP, KEGG, and Reactome, have been significantly developed.

## 10 Integration of metabolomics with genomics, transcriptomics, and proteomics

Metabolomics enhances genomic studies by linking genetic variations to specific metabolic traits. Genome-wide association studies (GWAS), along with metabolomics, have pinpointed specific loci that regulate metabolite accumulation in crops like rice and maize (Chen et al., 2016). In maize, a combination of GWAS and metabolomics has pinpointed loci that regulate the levels of essential amino acids and secondary metabolites, which is beneficial for increasing crop nutrition. Genomic information can forecast genes that code for enzymes, while metabolomics confirms the functionality of these enzymes by identifying intermediates and end products in metabolic pathways. In rice, the merging of genomic and metabolomics data has revealed novel pathways that show an important role in drought stress tolerance.

Combining metabolomics with transcriptomics aids in understanding the regulatory networks that govern metabolic pathways (Sanches et al., 2024). For instance, combining these methods in *Arabidopsis* showed how transcriptional regulation affects the secondary metabolites production under stress conditions. Studies examining gene expression and metabolite profiles during stress conditions highlight the timing and coordination of metabolic pathways. In tomatoes, a combined analysis of transcriptomics and metabolomics clarified how secondary metabolites are regulated throughout the fruit ripening process (Liu et al., 2023). Proteomics helps identify the enzymes that play a role in metabolic pathways, whereas metabolomics measures the intermediates in these pathways to confirm enzyme activity. For instance, combining proteomics and metabolomics in barley has uncovered enzymes that are involved in the metabolic changes triggered by drought (Chmielewska et al., 2016).

Proteomics identifies post-translational modifications (PTMs) like phosphorylation and acetylation that influence enzyme activity, whereas metabolomics uncovers the subsequent metabolic effects of these changes. By combining proteomics and metabolomics, researchers can build metabolic networks that aid in forecasting how systems respond to environmental shifts. By combining metabolomics with other technologies like genomics and proteomics, researchers can develop a comprehensive understanding of entire plant systems and their responses to shifting environmental conditions (Yang et al., 2021).

In tomato, various omics-based techniques, like metabolomics, proteomics, transcriptomics, etc., have been used to understand the plant’s response against abiotic stress (Naik et al., 2023). Under Cd stress in *Pistia stratiotes*, eight metabolic pathways and 3,107 differently expressed genes involved in various metabolic pathways were identified (Wei et al., 2023). In soybean, transcriptomics analysis shows significant changes in transcription factors and in some signaling pathways. In maize, the leaf responses during drought, salinity, and heat stress were analyzed at the metabolome and transcriptome levels. Reduction of essential glycolysis and tricarboxylic acid cycle intermediates, along with a noticeable buildup of amino acids like proline, arginine, and γ-amino butyrate, suggested a change in the equilibrium of carbon and nitrogen metabolism in leaves (Joshi et al., 2021). According to proteomic data, phosphopyruvate hydrase was enriched after salt stress and exhibited salt-induced DNA methylation. Additionally, it interacts with various metabolic pathway enzymes. The production of several metabolites was revealed using metabolomics data (Fu et al., 2024).

### 10.1 Integration of omics in stress-tolerant crop breeding

Abiotic stress induces drastic metabolic alterations in plants, which can lead to reduced yields and quality. To overcome this stress, the integration of omics into the crop breeding process has emerged as a viable strategy for the development of stress-tolerant crop varieties (Figure 4). Metabolomics provides information on the biochemical changes triggered by stress and is useful for identifying metabolic traits related to resilience (Satrio et al., 2024). It also connects phenotype and genotype by identifying the end products of cellular processes (Isah, 2019).

## 11 Metabolomics in crop improvement

Metabolomics is a comprehensive understanding of the biochemical conditions of the plant, with the ability of researchers to identify metabolic pathways and metabolites showing a response toward stress that can be utilized for an improvement in the crop quality. It provides high-throughput phenotyping through which genotypes tolerant to the stress can be identified by targeting metabolites responsible for stress tolerance (Raza et al., 2023). In rice, the metabolic profiling has detected important biomarkers, including sugars, flavonoids, and polyamines associated with drought tolerance (Ghorbanzadeh et al., 2023). Similarly, under drought stress in maize, metabolomics revealed the accumulation of amino acids, organic acids, and sugars, indicating their function in maintaining ROS detoxification and osmotic balance (Wang R. et al., 2024). Metabolomics has a vital role in the development of nutritionally enhanced crops by identifying the important metabolic pathways that contribute to the production of vitamins, amino acids, and bioactive compounds (Gandhi and Singh, 2024).

## 12 Conclusion

This review highlights the emerging evidence that plants’ resistance to abiotic stresses is the result of a well-organized metabolic reprogramming, which includes the production of osmolytes, the strengthening of antioxidant defense systems, and the changing of lipids on a large scale. The storage of compatible solutes such as soluble sugars, proline, glycine betaine, and raffinose family oligosaccharides is a conserved adaptive strategy across drought, salinity, cold, and heat stresses, helping with osmotic adjustment, cellular protection, and the preservation of metabolic homeostasis. The increased production of antioxidants like ascorbic acid, flavonoids, carotenoids, and polyphenols is of paramount importance in the prevention of oxidative damage and maintaining photosynthetic process efficiency, which are both caused by stress. Lipid remodeling, mainly the changes in fatty acid saturation and membrane lipid composition, also supports the plants to stay intact and functional in terms of membrane under severe heat and salt stress.

In rice, for example, elevated levels of proline, soluble sugars, and anaerobic metabolism-associated organic acids correlate with enhanced submergence and drought tolerance, while in wheat, accumulation of osmoprotectants and phenylpropanoid-derived antioxidants has been linked to improved performance under water deficit and temperature extremes. Similarly, maize genotypes exhibiting increased organic acid flux and secondary metabolite accumulation display superior tolerance to heat and drought stress.

In addition to improving mechanistic knowledge, the responses centered on metabolites compiled in the current review provide obvious and practical chances for the enhancement of crops in the case of important cereals and staple crops. Amongst the metabolites produced due to stress that comprise proline, glycine betaine, raffinose family oligosaccharides, malate, GABA, ascorbate, flavonoids, and unsaturated fatty acids, rice (*Oryza sativa*), wheat (*T. aestivum*), and maize (*Zea mays*) have been repeatedly linked with their stress tolerance, indicating their potential to serve as reliable metabolic biomarkers. Metabolomics-assisted breeding programs can make use of targeted metabolomics platform integration for fast screening of different germplasm panels and breeding populations, which eventually leads to early stress-resilient genotype identification under drought, salinity, heat, and waterlogging conditions. For instance, in rice, high amounts of proline, soluble sugars, and organic acids are related to anaerobic metabolism and are directly related to better tolerance to flooding and drought. Alternatively, in wheat, the accumulation of osmoprotectants and phenolic antioxidants has been associated with better performance under water deficit and heat extremes. Likewise, corn varieties that show increased organic acid flow and secondary metabolite buildup have a higher tolerance to heat and drought stress.

Genetic engineering has made significant progress, which allows for direct interventions at crucial metabolic and regulatory sites that cause this manipulation of various adaptive traits. In a case study of rice, the specific adjustment of osmolyte biosynthesis enzymes like pyrroline-5-carboxylate synthase and betaine aldehyde dehydrogenase has led to the improvement of the plant’s resistance to drought and salinity, while the alteration of fatty acid desaturases results in the enhancement of membrane stability at high temperatures. The engineering of antioxidant enzyme genes and transcription factors from the NAC and WRKY families in wheat has led to better redox balance and yield stability under adverse environmental conditions. In the case of maize, the regulation of enzymes involved in the metabolism of organic acids and the production of secondary metabolites, including those associated with the TCA cycle and the phenylpropanoid pathway, is showing great promise for the cultivation of multi-stress-tolerant maize varieties. It is critical to target the upstream stress-responsive transcription factors such as NAC, MYB, WRKY, and bHLH proteins, as they provide a synchronized approach to reprogramming the whole metabolic networks rather than only the isolated pathways. Such pathway-level interventions, when accurately adjusted to minimize the trade-offs between growth and stress tolerance, have very good potential in the production of high-yield and multi-stress resistant crop varieties that will be able to grow in different and changing agro-climatic environments.

The integration of high-resolution metabolomics, CRISPR/Cas-mediated genome editing, and AI-powered predictive modeling will change how we design stress-resilient crops. It is possible to establish systematic connections between pathway-level metabolic states, the underlying genetic variation, and the environmental complexity. Changes in mass spectrometry- and NMR-based metabolomics have now made it possible to do a thorough, spatially resolved, and time-dependent analysis of metabolites that are stress-responsive, thus helping to uncover the metabolic nodes and steps that are controlling the fluxes and that are associated with tolerance phenotypes. The metabolomic signatures, when correlated with the genome-wide association studies (mGWAS) and quantitative trait locus (QTL) mapping, can be linked to stress-related genes and regulatory elements directly. CRISPR/Cas genome editing offers an accurate and large-scale platform for target validation and engineering that enables the slight alteration of the activities of enzymes, transcription factors, and the points of metabolic branching within pathways like the TCA cycle, GABA shunt, and shikimate pathway. Simultaneously, processes that are based on AI and machine learning can be incorporated with multi-omics datasets along with environmental and phenotypic data to represent the nonlinear stress reactions of complexity, anticipating the interplay between the genotype and the environment, and giving preference to the best metabolic configurations for the particular agro-climatic conditions. This integrative framework makes it possible to transition from empirical selection to predictive selection based on pathways, thus speeding the development of climate-resilient varieties that can maintain their productivity even under the highly variable and multifactorial stress environments of the future (Yang X. et al., 2024).

## 13 Future perspectives and emerging directions

A crucial aspect of future research on plant stress biology is to study the metabolism of plants facing combined and sequential stress conditions (Afzal et al., 2021; Ahmed et al., 2022; Saini et al., 2024). In nature, plants face combined and/or sequential stresses, instead of a single stress. They must cope with combined stress interactions such as drought-heat, salt-nutrient deficit, or heavy metals-oxidative conditions (Amjad et al., 2024). There is growing evidence that metabolism under stress interactions is often non-additive and cannot be predicted by studying single-stress responses (Bharath et al., 2021; Bueno and Lopes, 2020). The specifics of each stress interaction must be investigated in view of metabolic conditions and signaling interactions (Chmielewska et al., 2016; Carrera et al., 2021; Chen Y. et al., 2023). Further research on plant stress biology along these lines, especially relating to time-course analyses on plant metabolism, would be most critical for understanding universal versus specific plant stress signatures.

Another emerging and important investigation is the fusion of metabolomics with predictive and computational modeling approaches (Meng et al., 2022; Chong and Xia, 2017; Kar and Leszczynski, 2017; Ghosh et al., 2020). As metabolomics provides high-dimensional datasets characterizing stress-induced biochemistries, the predictability and potential within these data have been largely untapped. Utilization of metabolomics data for predictive modeling with genome-resolved metabolic models, flux balance analysis, and isotope tracing may offer a way for making quantitative predictions regarding pathway orientation, metabolic bottlenecks, and resource allocation under stress. Collectively, artificial intelligence (AI) and machine learning-based multi-omics fusion analysis are being increasingly employed for the elucidation of nonlinear correlations between metabolite data, gene expression data, environmental parameters, and phenotypes for the diagnosis and prediction of stress and yield (Baishya et al., 2024). Major translational opportunities exist within the context of constructing metabolomics-assisted breeding platforms. Stress traits for plants tend to be polygenic and show large genotype × environment interactions; consequently, traditional plant breeding systems tend to be less efficient (Mahmood et al., 2022). Metabolomics bridges this gap by offering an approach that helps detect metabolic biomarkers, mQTL, and genome-wide association signals related to stress traits and plant quality (Wang et al., 2025b). The integration of metabolite markers with genome selection platforms may improve plant selection efficiency during earlier stages of plant development or when faced with varied environmental factors.

In future breeding programs, attention could be given to tissue and development stage-specific metabolism responses that are usually overlooked in whole plant metabolism calculations and might obscure localized stress responses associated with tissue-level metabolism responses (Taylor et al., 2021). Recent advances in spatial and single-cell metabolomics are foreseen to bring a paradigm shift in understanding tissue-level metabolism differences and hence improve trait discovery and selection (Taylor et al., 2021; Petrova and Guler, 2024). Taken together, “multi-stress metabolomics,” “predictive modeling,” and “metabolomics-assisted breeding” represent a major emerging direction in crop science. Embedding metabolomic data within breeding pipelines and decision-support systems will enable a shift from reactive stress mitigation toward “predictive, design-based crop resilience,” a condition under which agricultural productivity and food security can be sustained in the face of accelerating climate change.

## References

[B1] Acidri R. Sawai Y. Sugimoto Y. Handa T. Sasagawa D. Masunaga T. (2020). Exogenous kinetin promotes the nonenzymatic antioxidant system and photosynthetic activity of coffee (coffea arabica l.) plants under cold stress conditions. Plants 9 (2), 281. 10.3390/plants9020281 32098166 PMC7076472

[B3] Afzal S. Chaudhary N. Singh N. K. (2021). Role of soluble sugars in metabolism and sensing under abiotic stress. arXiv, 305–334. 10.1007/978-3-030-61153-8_14

[B4] Afzal M. Javed M. Aroob S. Javed T. Alnoman M. Alelwani W. (2023). The biogenic synthesis of bimetallic Ag/ZnO nanoparticles: A multifunctional for methyl violet photocatalytic degradation and the assessment of antibacterial, antioxidant, and cytotoxicity properties. Nanomaterials 13, 2079. 10.3390/nano13142079 37513090 PMC10385465

[B7] Ahlawat Y. K. Singh M. Manorama K. Lakra N. Zaid A. Zulfiqar F. (2024). Plant phenolics: Neglected secondary metabolites in plant stress tolerance. Braz. J. Bot. 47, 703–721. 10.1007/s40415-023-00949-x

[B8] Ahmad I. Song X. Hussein Ibrahim M. E. Jamal Y. Younas M. U. Zhu G. (2023). The role of melatonin in plant growth and metabolism, and its interplay with nitric oxide and auxin in plants under different types of abiotic stress. Front. Plant Sci. 14, 1108507. 10.3389/fpls.2023.1108507 36866369 PMC9971941

[B9] Ahmed M. Decsi K. Tóth Z. (2022). Different tactics of synthesized zinc oxide nanoparticles, homeostasis ions, and phytohormones as regulators and adaptatively parameters to alleviate the adverse effects of salinity stress on plants. Life 12, 1–19. 10.3390/life13010019 PMC9867113 36676021

[B10] Akram M. Sajid Z. Farooq A. B. U. Ahmad I. Jamal A. Rizwana H. (2024). Characterization of physiological and biochemical attributes of neem (azadirachta indica a. Juss) under salinity stress. Horticulturae 10, 702. 10.3390/horticulturae10070702

[B11] Al-Khayri J. Rashmi R. Ulhas R. Sudheer W. Banadka A. Nagella P. (2023). The role of nanoparticles in response of plants to abiotic stress at physiological, biochemical, and molecular levels. Plants 12. 10.3390/plants12020292 PMC9865530 36679005

[B12] Alabdallah N. Hasan M. Hammami I. Alghamdi A. Alshehri D. Alatawi H. (2021). Green synthesized metal oxide nanoparticles mediate growth regulation and physiology of crop plants under drought stress. Plants 10, 1730. 10.3390/plants10081730 34451775 PMC8399390

[B13] Ali A. Santoro P. Mori J. Ferrante A. Cocetta G. (2024). Effect of UV-B elicitation on spearmint (mentha spicata l.) morpho-physiological traits and secondary metabolites production. Plant Growth Regul. 104, 63–76. 10.1007/s10725-023-01028-7

[B14] Alonso A. Marsal S. Julià A. (2015). Analytical methods in untargeted metabolomics: State of the art in 2015. Front. Bioeng. Biotechnol. 3, 23. 10.3389/fbioe.2015.00023 25798438 PMC4350445

[B15] Alseekh S. Bermudez L. Haro L. A. Fernie A. R. Carrari F. (2018). Crop metabolomics: From diagnostics to assisted breeding. Metabolomics 14, 148. 10.1007/s11306-018-1446-5 30830402

[B16] Amberg A. Riefke B. Schlotterbeck G. Ross A. Senn H. Dieterle F. (2017). NMR and MS methods for metabolomics. Methods Mol. Biol. 1641, 229–258. 10.1007/978-1-4939-7172-5_13 28748468

[B228] Amiri R. Nikbakht A. Etemadi N. (2015). Alleviation of drought stress on rose geranium [Pelargonium graveolens (L.) Herit.] in terms of antioxidant activity and secondary metabolites by mycorrhizal inoculation. Sci. Hortic. 197, 373–380.

[B17] Amiripour A. Jahromi M. G. Soori M. K. Torkashvand A. M. (2021). Changes in essential oil composition and fatty acid profile of coriander (*Coriandrum sativum* l.) leaves under salinity and foliar-applied silicon. Indust Crops Prod. 168, 113599. 10.1016/j.indcrop.2021.113599

[B18] Amjad E. Sokouti B. Asnaashari S. Dastmalchi S. (2024). A systematic review on the role of SnRK2 gene in arabidopsis thaliana growth stages under abiotic stresses. OBM Genet. 8, 1–26. 10.21926/obm.genet.2404275

[B19] Ammar M. K. Hanafi R. S. Choucry M. A. Handoussa H. (2023). Structural, functional, nutritional composition and analytical profiling of *Triticum aestivum* l. Appl. Biol. Chem. 66, 48. 10.1186/s13765-023-00804-3

[B20] Andrade A. Boero A. Escalante M. Llanes A. Arbona V. Gómez-Cádenas A. (2021). Comparative hormonal and metabolic profile analysis based on mass spectrometry provides information on the regulation of water-deficit stress response of sunflower (*Helianthus annuus* l.) inbred lines with different water-deficit stress sensitivity. Plant Physiol. Biochem. 168, 432–446. 10.1016/j.plaphy.2021.10.015 34715568

[B21] Anzano A. Bonanomi G. Mazzoleni S. Lanzotti V. (2022). Plant metabolomics in biotic and abiotic stress: a critical overview. Phytochem. Rev. 21, 503–524. 10.1007/s11101-021-09786-w

[B22] Arrivault S. Alexandre Moraes T. Obata T. Medeiros D. B. Fernie A. R. Boulouis A. (2019). Metabolite profiles reveal interspecific variation in operation of the Calvin–Benson cycle in both C4 and C3 plants. J. Experimental Botany 70 (6), 1843–1858. 10.1093/jxb/erz051 30773587 PMC6436152

[B23] Ashapkin V. V. Kutueva L. I. Aleksandrushkina N. I. Vanyushin B. F. (2020). Epigenetic mechanisms of plant adaptation to biotic and abiotic stresses. Int. J. Mol. Sci. 21, 7457. 10.3390/ijms21207457 33050358 PMC7589735

[B24] Aszyk J. Byliński H. Namieśnik J. Kot-Wasik A. (2018). Main strategies, analytical trends and challenges in LC-MS and ambient mass Spectrometry–Based metabolomics. TrAC Trends Anal. Chem. 108, 278–295. 10.1016/j.trac.2018.09.010

[B25] Baek S.-A. Kim K. W. Kim J. O. Kim T. J. Ahn S. K. Choi J. (2021). Metabolic profiling reveals an increase in stress-related metabolites in *Arabidopsis thaliana* exposed to honeybees. J. Appl. Biol. Chem. 64, 141–151. 10.3839/jabc.2021.021

[B26] Baishya S. Nath D. Nath R. Nahar L. Sarker S. D. Talukdar A. D. (2024). Mathematical models and computation in plant metabolomics: An update. Elsevier, 301–320.

[B28] Bertamini M. Grando M. S. Zocca P. Pedrotti M. Lorenzi S. Cappellin L. (2019). Linking monoterpenes and abiotic stress resistance in grapevines. Les Ulis, France: EDP Sciences. 10.1051/bioconf/20191301003

[B29] Bhambhani S. Lakhwani D. Gupta P. Pandey A. Dhar Y. V. Kumar Bag S. (2017). Transcriptome and metabolite analyses in Azadirachta indica: identification of genes involved in biosynthesis of bioactive triterpenoids. Sci. Reports 7 (1), 5043. 10.1038/s41598-017-05291-3 28698613 PMC5505991

[B30] Bharadwaj B. Sanathanam S. K. Pham T. Cantrell C. L. Wang M. Lee J. (2024). Physiological and biochemical responses of turmeric (*Curcuma longa* l.) under drought stress. J. Med. Act. Plants 13, 2–3. 10.7275/jmap.2315

[B31] Bharath P. Gahir S. Raghavendra A. S. (2021). Abscisic acid-induced stomatal closure: An important component of plant defense against abiotic and biotic stress. Front. Plant Sci. 12, 615114. 10.3389/fpls.2021.615114 33746999 PMC7969522

[B33] Blaise B. J. Correia G. D. Haggart G. A. Surowiec I. Sands C. Lewis M. R. (2021). Statistical analysis in metabolic phenotyping. Nat. Protoc. 16, 4299–4326. 10.1038/s41596-021-00579-1 34321638

[B34] Bocso N. S. Butnariu M. (2022). The biological role of primary and secondary plants metabolites. J. Nutr. Food Process. 5, 1–7. 10.31579/2637-8914/094

[B35] Braga O. Silva T. L. C. Silva V. N. B. Neto J. C. R. Ribeiro A. Abdelnur P. V. (2022). Deep untargeted metabolomics analysis to further characterize the adaptation response of *Gliricidia sepium* to very high salinity stress. Front. Plant Sci. 13, 869105. 10.3389/fpls.2022.869105 35665181 PMC9161747

[B36] Brillante L. Martínez-Lüscher J. Kurtural S. K. (2018). Applied water and mechanical canopy management affect berry and wine phenolic and aroma composition of grapevine (*Vitis vinifera* l., cv. Syrah) in central California. Sci. Hortic. 227, 261–271. 10.1016/j.scienta.2017.09.048

[B37] Bu R. Qiu Z. Dong J. Chen L. Zhou Y. Wang H. (2025). The role of SQUAMOSA-PROMOTER BINDING PROTEIN-Like (SPL) transcription factors in plant growth and environmental stress response: A comprehensive review of recent advances. Horticulturae 11, 584. 10.3390/horticulturae11060584

[B38] Bueno P. C. Lopes N. P. (2020). Metabolomics to characterize adaptive and signaling responses in legume crops under abiotic stresses. ACS Omega 5, 1752–1763. 10.1021/acsomega.9b03668 32039310 PMC7003242

[B39] Butnariu M. Butu A. (2019). Plant nanobionics: Application of nanobiosensors in plant biology. Springer International Publishing, 337–376.

[B40] Caliskan O. Kurt D. Temizel K. E. Odabas M. S. (2017). Effect of salt stress and irrigation water on growth and development of sweet basil (*Ocimum basilicum* L.). Open Agric. 2, 589–594. 10.1515/opag-2017-0062

[B41] Cambiaghi A. Ferrario M. Masseroli M. (2017). Analysis of metabolomic data: Tools, current strategies and future challenges for omics data integration. Briefings Bioinforma. 18, 498–510. 10.1093/bib/bbw031 27075479

[B43] Cao K. Wang S. Zhang H. Ma Y. Wu Q. Huang F. (2025). Transcriptomic Regulatory Mechanisms of Isoflavone Biosynthesis in *Trifolium pratense* . Agronomy 15, 1061. 10.3390/agronomy15051061

[B44] Carrera F. P. Noceda C. Maridueña-Zavala M. G. Cevallos-Cevallos J. M. (2021). Metabolomics, a powerful tool for understanding plant abiotic stress. Agronomy 11, 824. 10.3390/agronomy11050824

[B45] Chalecka M. Kazberuk A. Palka J. Surazynski A. (2021). P5C as an interface of proline interconvertible amino acids and its role in regulation of cell survival and apoptosis. Int. Journal Molecular Sciences 22, 11763. 10.3390/ijms222111763 34769188 PMC8584052

[B47] Chauhan J. Prathibha M. D. Singh P. Choyal P. Mishra U. N. Saha D. (2023). Plant photosynthesis under abiotic stresses: Damages, adaptive, and signaling mechanisms. Plant Stress 10, 100296. 10.1016/j.stress.2023.100296

[B48] Chen Y. C. Yuan R. S. Ao P. Xu M. J. Zhu X. M. (2016). Towards stable kinetics of large metabolic networks: Nonequilibrium potential function approach. Phys. Rev. E 93 (6), 062409. 10.1103/PhysRevE.93.062409 27415300

[B49] Chen Q. Yu Y. Zhang X. Zhao R. Zhang J. Liu D. (2021). The transcription factor PjERF1 enhances the biosynthesis of triterpenoid saponins in Panax japonicus. Plant Biotechnol. Rep. 15 (5), 597–607. 10.1007/s11816-021-00698-x

[B50] Chen D. Mubeen B. Hasnain A. Rizwan M. Adrees M. Naqvi S. A. H. (2022). Role of promising secondary metabolites to confer resistance against environmental stresses in crop plants: Current scenario and future perspectives. Front. Plant Sci. 13, 881032. 10.3389/fpls.2022.881032 35615133 PMC9126561

[B51] Chen Y. Liu M. Wen J. Yang Z. Li G. Cao Y. (2023). Panax japonicus CA Meyer: a comprehensive review on botany, phytochemistry, pharmacology, pharmacokinetics and authentication. Chin. Med. 18, 148. 10.1186/s13020-023-00857-y 37950271 PMC10636818

[B52] Chen L. Meng Y. Bai Y. Yu H. Qian Y. Zhang D. (2023). Starch and sucrose metabolism and plant hormone signaling pathways play crucial roles in aquilegia salt stress adaption. Int. J. Mol. Sci. 24, 3948. 10.3390/ijms24043948 36835360 PMC9966690

[B53] Chen Z. Dong Y. Huang X. (2022). Plant responses to UV-B radiation: Signaling, acclimation and stress tolerance. Stress Biol. 2, 51. 10.1007/s44154-022-00076-9 37676395 PMC10441900

[B421] Chen Q. Li N. Cui X. Ge F. (2025). AP2/ERF transcription factors regulate the biosynthesis of terpenoids, phenolics, and alkaloids in plants. Horticult. Res., uhaf280. 10.1093/hr/uhaf280 PMC12871079 41647723

[B54] Chmielewska K. Rodziewicz P. Swarcewicz B. Sawikowska A. Krajewski P. Marczak Ł. (2016). Analysis of drought-induced proteomic and metabolomic changes in barley (hordeum vulgare l.) leaves and roots unravels biochemical mechanisms involved in drought tolerance. Front. Plant Sci. 7, 1108. 10.3389/fpls.2016.01108 27512399 PMC4962459

[B55] Cho Y. Choi Y. Jang Y. Seong H. (2025). Nanomaterial‐Enhanced biosensing: Mechanisms and emerging applications. Adv. Healthc. Mater. 14, 2500189. 10.1002/adhm.202500189 40167299 PMC12683224

[B56] Chong J. Xia J. (2017). Computational approaches for integrative analysis of the metabolome and microbiome. Metabolites 7 (4), 62. 10.3390/metabo7040062 29156542 PMC5746742

[B418] Choudhary K. K. Agrawal S. B. (2014). Ultraviolet-B induced changes in morphological, physiological and biochemical parameters of two cultivars of pea (Pisum sativum L.). Ecotoxicol. Environ. Safety. 100, 178–187. 10.1016/j.ecoenv.2013.10.032 24268741

[B57] Choudhary S. Wani K. I. Naeem M. Khan M. M. A. Aftab T. (2023). Cellular responses, osmotic adjustments, and role of osmolytes in providing salt stress resilience in higher plants: Polyamines and nitric oxide crosstalk. J. Plant Growth Regul. 42, 539–553. 10.1007/s00344-022-10584-7

[B58] Chrysargyris A. Panayiotou C. Tzortzakis N. (2016). Nitrogen and phosphorus levels affected plant growth, essential oil composition and antioxidant status of lavender plant (*Lavandula angustifolia* mill.). Industrial Crops Prod. 83, 577–586. 10.1016/j.indcrop.2015.12.067

[B60] Coniglio S. Shumskaya M. Vassiliou E. (2023). Unsaturated fatty acids and their immunomodulatory properties. Biology 12, 279. 10.3390/biology12020279 36829556 PMC9953405

[B61] Costine B. Zhang M. Pearson B. Nadakuduti S. S. (2022). Impact of blue light on plant growth, flowering and accumulation of medicinal flavones in scutellaria baicalensis and s. lateriflora. Horticulturae 8, 1141. 10.3390/horticulturae8121141

[B62] Couillaud J. Leydet L. Duquesne K. Iacazio G. (2021). The Terpene Mini-Path, a New Promising Alternative for Terpenoids Bio-Production. Genes 12, 1974. 10.3390/genes12121974 34946923 PMC8701039

[B189] da Silva Magedans Y. V. Phillips M. A. Fett-Neto A. G. (2021). Production of plant bioactive triterpenoid saponins: from metabolites to genes and back. Phytochem. Rev. 20 (2), 461–482. 10.1007/s11101-020-09722-4

[B63] Dawid C. Hille K. (2018). Functional metabolomics—a useful tool to characterize stress-induced metabolome alterations opening new avenues towards tailoring food crop quality. Agronomy 8, 138. 10.3390/agronomy8080138

[B64] Deborde C. Moing A. Roch L. Jacob D. Rolin D. Giraudeau P. (2017). Plant metabolism as studied by NMR spectroscopy. Prog. Nuclear Magnetic Resonance Spectroscopy 102, 61–97. 10.1016/j.pnmrs.2017.05.001 29157494

[B65] Deng M. Zhang X. Luo J. Liu H. Wen W. Luo H. (2020). Metabolomics analysis reveals differences in evolution between maize and rice. Plant J. 103, 1710–1722. 10.1111/tpj.14856 32445406

[B66] Derakhshani Z. Bhave M. Shah R. M. (2020). Metabolic contribution to salinity stress response in grains of two barley cultivars with contrasting salt tolerance. Environ. Exp. Bot. 179, 104229. 10.1016/j.envexpbot.2020.104229

[B67] Dilnawaz F. Misra A. Apostolova E. (2023). Involvement of nanoparticles in mitigating plant’s abiotic stress. Plant Stress 10, 100280. 10.1016/j.stress.2023.100280

[B68] Dinakar C. Vishwakarma A. Raghavendra A. S. Padmasree K. (2016). Alternative oxidase pathway optimizes photosynthesis during osmotic and temperature stress. Front. Plant Sci. 7, 68. 10.3389/fpls.2016.00068 26904045 PMC4747084

[B69] Dong X. M. Pu X. J. Zhou S. Z. Li P. Luo T. Chen Z. X. (2022). Orphan gene PpARDT positively involved in drought tolerance potentially by enhancing ABA response in physcomitrium patens. Plant Sci. 319, 111222. 10.1016/j.plantsci.2022.111222 35487672

[B230] Dubey K. K. D. Yadav J. Upadhyaya K. C. Kumar A. (2020). Accumulation of ricinoleic acid in developing seeds of castor (Ricinus communis) from India. Indian J. Agric. Sci. 90 (11), 2145–2149. 10.56093/ijas.v90i11.108579

[B70] Dubey S. Virmani T. Yadav S. Sharma A. Kumar G. Alhalmi A. (2024). Breaking barriers in eco-friendly synthesis of plant-mediated Metal/Metal Oxide/Bimetallic nanoparticles: Antibacterial, anticancer, mechanism elucidation, and versatile utilizations. J. Nanomater. 2024, 1–48. 10.1155/2024/9914079

[B420] Ejaz S. Fahad S. Anjum M. A. Nawaz A. Naz S. Hussain S. Ahmad S. (2020). “Role of osmolytes in the mechanisms of antioxidant defense of plants,” in Sus. Agricul. Rev. (Cham: Springer International Publishing) 39, 95–117. 10.1007/978-3-030-38881-2_4

[B71] El Amerany F. (2024). “The role of terpenoids in plant development and stress tolerance,” in Molecular and physiological insights into plant stress tolerance and applications in agriculture—Part 2 (Sharjah, United Arab Emirates: Bentham Science Publishers), 71–98.

[B72] El-Nasr A. Hassan K. Abd-Elhalim B. Kucher D. Rebouh N. Ansabayeva A. (2025). The emerging roles of nanoparticles in managing the environmental stressors in horticulture Crops—A review. Plants 14. 10.3390/plants14142192 PMC12298009 40733428

[B73] Elango D. Rajendran K. Laan L. Sebastiar S. Raigne J. Thaiparambil N. A. (2022). Raffinose family oligosaccharides: Friend or foe for human and plant health? Front. Plant Sci. 13, 829118. 10.3389/fpls.2022.829118 35251100 PMC8891438

[B74] El-Saadony M. Saad A. Soliman S. Salem H. Desoky E. Babalghith A. (2022). Role of Nanoparticles in Enhancing Crop Tolerance to Abiotic Stress: A Comprehensive Review. Front. Plant Sci. 13, 946717. 10.3389/fpls.2022.946717 36407622 PMC9670308

[B75] Faisal M. Faizan M. Soysal S. Alatar A. (2024). Synergistic application of melatonin and silicon oxide nanoparticles modulates reactive oxygen species generation and the antioxidant defense system: a strategy for cadmium tolerance in rice. Front. Plant Sci. 15, 1484600. 10.3389/fpls.2024.1484600 39474214 PMC11518792

[B76] Feller U. Vaseva I. I. (2014). Extreme climatic events: impacts of drought and high temperature on physiological processes in agronomically important plants. Front. Environ. Sci. 2, 39. 10.3389/fenvs.2014.00039

[B77] Feng Z. Ding C. Li W. Wang D. Cui D. (2020). Applications of metabolomics in soybean under abiotic stress. Food Chem. 310, 125914. 10.1016/j.foodchem.2019.125914 31835223

[B78] Feng K. Yan Y. J. Sun N. Yang Z. Y. Zhao S. P. Wu P. (2024). Exogenous methyl jasmonate treatment induced the transcriptional responses and accumulation of volatile terpenoids in oenanthe javanica (blume) DC. Int. J. Biol. Macromol. 265, 131017. 10.1016/j.ijbiomac.2024.131017 38513909

[B79] Feng Y. Li Z. Kong X. Khan A. Ullah N. Zhang X. (2025). Plant coping with cold stress: Molecular and physiological adaptive mechanisms with future perspectives. Cells 14, 110. 10.3390/cells14020110 39851537 PMC11764090

[B80] Feussner I. Polle A. (2015). What the transcriptome does not tell—proteomics and metabolomics are closer to the plants’ patho-phenotype. Curr. Opinion Plant Biology 26, 26–31. 10.1016/j.pbi.2015.05.023 26051215

[B81] Fitoussi N. Borrego E. Kolomiets M. V. Qing X. Bucki P. Sela N. (2021). Oxylipins as communication signals in Tomato–Root-Knot nematode interaction. Sci. Rep. 11, 326. 10.1038/s41598-020-79432-6 33431951 PMC7801703

[B82] Folgado R. Sergeant K. Renaut J. Swennen R. Hausman J. F. Panis B. (2014). Changes in sugar content and proteome of potato under cold and dehydration stress. J. Proteomics 98, 99–111. 10.1016/j.jprot.2013.11.027 24333155

[B83] Fu S. Wang L. Li C. Zhao Y. Zhang N. Yan L. (2024). Integrated transcriptomic, proteomic, and metabolomic analyses reveal mechanisms for salt resistance in soybean seedlings. Int. J. Mol. Sci. 25, 13559. 10.3390/ijms252413559 39769326 PMC11678865

[B84] Furnell H. Wenger J. Wingler A. Kilcawley K. N. Mannion D. T. Skibinska I. (2024). Identification of volatile organic compounds emitted by sitka spruce and determination of their emission pathways and fluxes. EGUsphere 2024, 1–37. 10.5194/egusphere-2024-154

[B85] Gajewska E. Witusińska A. Bernat P. (2024). Nickel-induced oxidative stress and phospholipid remodeling in cucumber leaves. Plant Sci. 348, 112229. 10.1016/j.plantsci.2024.112229 39151803

[B86] Gandhi N. Singh A. P. (2024). Molecular Approaches for Improving Nutritional Quality in Crops. Nonthermal Food Process. Saf. Preserv., 449–477. 10.1002/9781394186631.ch22

[B87] Garcia-Caparros P. Filippis L. Gul A. Hasanuzzaman M. Ozturk M. Altay V. (2021). Oxidative stress and antioxidant metabolism under adverse environmental conditions: A review. Botanical Rev. 87, 421–466. 10.1007/s12229-020-09231-1

[B88] Ghani M. Saleem S. Rather S. Rehmani M. Alamri S. Rajput V. (2021). Foliar application of zinc oxide nanoparticles: An effective strategy to mitigate drought stress in cucumber seedling by modulating antioxidant defense system and osmolytes accumulation. Chemosphere 289, 133202. 10.1016/j.chemosphere.2021.133202 34890613

[B89] Ghasemi S. Kumleh H. H. Kordrostami M. Rezadoost M. H. (2023). Drought stress-mediated alterations in secondary metabolites and biosynthetic gene expression in cumin plants: Insights from gene-specific and metabolite-level analyses. Plant Stress 10, 100241. 10.1016/j.stress.2023.100241

[B90] Ghatak A. Chaturvedi P. Weckwerth W. (2018). Metabolomics in plant stress physiology. Adv. Biochem. Eng. Biotechnol. 164, 187–236. 10.1007/10_2017_55 29470599

[B91] Ghazagh F. Bagherieh-Najjar M. B. Nezamdoost T. (2023). Unraveling the interaction of copper, cadmium, calcium, and nitrate on phenolics, flavonoids, and shikonin contents of onosma dichroantha calli by statistical modeling. Environ. Sci. Pollut. Res. 30, 43804–43816. 10.1007/s11356-023-25187-2 36662436

[B92] Ghorbanzadeh Z. Hamid R. Jacob F. Zeinalabedini M. Salekdeh G. H. Ghaffari M. R. (2023). Comparative Metabolomics of Root Tips Reveals Pathways Conferring Drought Tolerance in Rice Genotypes. BMC Genomics 24 (1), 152. 10.1186/s12864-023-09246-z 36973662 PMC10044761

[B93] Ghosh T. Zhang W. Ghosh D. Kechris K. Ghorbanzadeh Z. Hamid R. (2020). Predictive modeling for metabolomics data. In Computational methods data analysis metabolomics 24, 313–336. 10.1007/978-1-0716-0239-3_16 PMC7423323 31953824

[B94] Giebułtowicz J. Ślusarczyk J. Wyderska S. Wroczyński P. (2024). The Impact of Organic Selenium (IV) on *Hypericum perforatum* L. under Cadmium Stress and Non-Stress Conditions. Plants 13, 2099. 10.3390/plants13152099 39124217 PMC11314003

[B95] Głuchowska A. Zieniuk B. Pawełkowicz M. (2025). Unlocking plant resilience: Metabolomic insights into abiotic stress tolerance in crops. Metabolites 15, 384. 10.3390/metabo15060384 40559408 PMC12195149

[B96] Godbole R. C. Pable A. A. Singh S. Barvkar V. T. (2022). Interplay of transcription factors orchestrating the biosynthesis of plant alkaloids. 3 Biotech. 12, 250. 10.1007/s13205-022-03316-x 36051988 PMC9424429

[B97] Godoy F. Olivos-Hernández K. Stange C. Handford M. (2021). Abiotic stress in crop species: Improving tolerance by applying plant metabolites. Plants 10, 186. 10.3390/plants10020186 33498148 PMC7908993

[B98] Gómez-Bellot M. J. Garcia C. J. Parra A. Vallejo F. Ortuño M. F. (2023). Influence of drought stress on increasing bioactive compounds of pomegranate juice: An untargeted metabolomics study. Eur. Food Res. Technol. 249, 2947–2956. 10.1007/s00217-023-04340-8

[B99] Gong M. Jiang D. Liu R. Tian S. Xing H. Chen Z. (2023). Influence of high-temperature and intense light on the enzymatic antioxidant system in ginger (*Zingiber officinale* roscoe) plantlets. Metabolites 13, 992. 10.3390/metabo13090992 37755272 PMC10534589

[B100] González-Mendoza D. Troncoso-Rojas R. Gonzalez-Soto T. Grimaldo-Juarez O. Cecena-Duran C. Duran-Hernandez D. (2018). Changes in the phenylalanine ammonia lyase activity, total phenolic compounds, and flavonoids in *Prosopis glandulosa* treated with cadmium and copper. An. Acad. Bras. Ciências 90, 1465–1472. 10.1590/0001-3765201820170622 29898105

[B101] Gou C. Zafar S. Hasnain Z. Aslam N. Iqbal N. Abbas S. (2024). Machine and deep learning: artificial intelligence application in biotic and abiotic stress management in plants. Front. Bioscience-Landmark 29, 20. 10.31083/j.fbl2901020 38287813

[B102] Guo M. Wang X. S. Guo H. D. Bai S. Y. Khan A. Wang X. M. (2022). Tomato salt tolerance mechanisms and their potential applications. Front. Plant Sci. 13, 949541. 10.3389/fpls.2022.949541 36186008 PMC9515470

[B103] Guo L. Tan J. Deng X. Mo R. Pan Y. Cao Y. (2023). Integrated metabolome and transcriptome analysis reveals genes regulating flavonoid biosynthesis in pinellia ternata under heat stress. J. Plant Res. 136, 359–369. 10.1007/s10265-023-01446-8 36881276 PMC10126072

[B104] Gupta B. Pathak G. C. (2025). Role of nickel in plants: A review. Indian J. Appl. Pure Bio 40, 2175–2181.

[B105] Hamany Djande C. Y. Pretorius C. Tugizimana F. Piater L. A. Dubery I. A. (2020). Metabolomics: A tool for cultivar phenotyping and investigation of grain crops. Agronomy 10 (6), 831. 10.3390/agronomy10060831

[B106] Hanif S. Saleem M. F. Sarwar M. Irshad M. Shakoor A. Wahid M. A. (2021). Biochemically triggered heat and drought stress tolerance in rice by proline application. J. Plant Growth Regul. 40, 305–312. 10.1007/s00344-020-10095-3

[B107] Harfouche A. L. Jacobson D. A. Kainer D. Romero J. C. Harfouche A. H. Mugnozza G. S. (2019). Accelerating climate resilient plant breeding by applying next-generation artificial intelligence. Trends Biotechnology 37, 1217–1235. 10.1016/j.tibtech.2019.05.007 31235329

[B108] Hashemifar Z. Sanjarian F. Naghdi Badi H. Mehrafarin A. (2025). Impact of varying light intensities on morphology, phytochemistry, volatile compounds, and gene expression in Thymus vulgaris L. Plos One 20 (2), e0317840. 10.1371/journal.pone.0317840 40009615 PMC11864514

[B109] Hassan M. A. Xiang C. Farooq M. Muhammad N. Yan Z. Hui X. (2021). Cold stress in wheat: Plant acclimation responses and management strategies. Front. Plant Sci. 12, 676884. 10.3389/fpls.2021.676884 34305976 PMC8299469

[B110] Hazrati S. Farahbakhsh M. Cerdà A. Heydarpoor G. (2021). Functionalization of ultrasound enhanced sewage sludge-derived biochar: Physicochemical improvement and its effects on soil enzyme activities and heavy metals availability. Chemosphere 269, 128767. 10.1016/j.chemosphere.2020.128767 33131739

[B111] He J. Yao L. Pecoraro L. Liu C. Wang J. Huang L. (2023). Cold stress regulates accumulation of flavonoids and terpenoids in plants by phytohormone, transcription process, functional enzyme, and epigenetics. Crit. Rev. Biotechnol. 43, 680–697. 10.1080/07388551.2022.2053056 35848841

[B112] Hendricks K. (2021). Signalling molecule “calcium” improves germination and growth of Sorghum bicolor seedlings under salt stress. 10.3390/plants9060730 PMC7356090 32531914

[B113] Henschel J. M. Andrade A. N. D. Santos J. B. L. Silva R. R. Mata D. A. Souza T. (2024). Lipidomics in plants under abiotic stress conditions: An overview. Agronomy 14, 1670. 10.3390/agronomy14081670

[B114] Hnilickova H. Kraus K. Vachova P. Hnilicka F. (2021). Salinity stress affects photosynthesis, malondialdehyde formation, and proline content in portulaca oleracea l. Plants 10, 845. 10.3390/plants10050845 33922210 PMC8145623

[B115] Holopainen J. K. Himanen S. J. Yuan J. S. Chen F. Jr S. (2025). Ecological functions of terpenoids in changing climates. Springer, 1–44.

[B116] Hölzl G. Dörmann P. (2019). Chloroplast lipids and their biosynthesis. Annu. Rev. Plant Biol. 70, 51–81. 10.1146/annurev-arplant-050718-100202 30786236

[B117] Hosseinifard M. Stefaniak S. Javid M. G. Soltani E. Wojtyla Ł. Garnczarska M. (2022). Contribution of exogenous proline to abiotic stresses tolerance in plants: A review. Int. J. Mol. Sci. 23, 5186. 10.3390/ijms23095186 35563577 PMC9101538

[B118] Hou Q. Ufer G. Bartels D. (2016). Lipid signaling in plant responses to abiotic stress. Plant, Cell and Environ. 39, 1029–1048. 10.1111/pce.12666 26510494

[B119] Huan X. Li L. Liu Y. Kong Z. Liu Y. Wang Q. (2022). Integrating transcriptomics and metabolomics to analyze quinoa (*Chenopodium quinoa* willd.) responses to drought stress and rewatering. Front. Plant Sci. 13, 988861. 10.3389/fpls.2022.988861 36388589 PMC9645111

[B120] Huang L. Li P. Tian M. Feng X. Chen Y. Feng B. (2025). Comprehensive characterization of the WRKY gene family and their potential roles in regulation phenylphenalenone biosynthesis in *Musella lasiocarpa* . Front. Plant Sci. 16, 1570758. 10.3389/fpls.2025.1570758 40144758 PMC11936918

[B121] Ighodaro O. M. Akinloye O. A. (2018). First line defence antioxidants-superoxide dismutase (SOD), catalase (CAT) and glutathione peroxidase (GPX): Their fundamental role in the entire antioxidant defence grid. Alexandria Journal Medicine 54 (4), 287–293. 10.1016/j.ajme.2017.09.001

[B122] Innes S. N. Arve L. E. Zimmermann B. Nybakken L. Melby T. I. Solhaug K. A. (2019). Elevated air humidity increases UV-Mediated leaf and DNA damage in pea (pisum sativum) due to reduced flavonoid content and antioxidant power. Photochem. and Photobiological Sci. 18, 387–399. 10.1039/c8pp00401c 30480699

[B123] Isah T. (2019). Stress and defense responses in plant secondary metabolites production. Biol. Res. 52, 39. 10.1186/s40659-019-0246-3 31358053 PMC6661828

[B124] Jadaun J. S. Sangwan N. S. Narnoliya L. K. Singh N. Bansal S. Mishra B. (2017). Over‐expression of DXS gene enhances terpenoidal secondary metabolite accumulation in rose‐scented geranium and Withania somnifera: active involvement of plastid isoprenogenic pathway in their biosynthesis. Physiol. Plantarum 159 (4), 381–400. 10.1111/ppl.12507 27580641

[B125] Jadaun J. S. Kushwaha A. K. Sangwan N. S. Narnoliya L. K. Mishra S. Sangwan R. S. (2020). WRKY1-mediated regulation of tryptophan decarboxylase in tryptamine generation for withanamide production in Withania somnifera (Ashwagandha). Plant Cell Rep. 39, 1443–1465. 10.1007/s00299-020-02574-4 32789542

[B126] Jan R. Asaf S. Numan M. Lubna and Kim K. M. (2021). Plant secondary metabolite biosynthesis and transcriptional regulation in response to biotic and abiotic stress conditions. Agronomy 11, 968. 10.3390/agronomy11050968

[B127] Jang C. Chen L. Rabinowitz J. D. (2018). Metabolomics and isotope tracing. Cell 173, 822–837. 10.1016/j.cell.2018.03.055 29727671 PMC6034115

[B128] Javed T. Gao S. J. (2023). WRKY transcription factors in plant defense. Trends Genet. 39, 787–801. 10.1016/j.tig.2023.07.001 37633768

[B131] Jia H. Wang L. Li J. Sun P. Lu M. Hu J. (2020). Physiological and metabolic responses of *Salix sinopurpurea* and *Salix suchowensis* to drought stress. Trees 34, 563–577. 10.1007/s00468-019-01937-z

[B132] Jia C. Guo B. Wang B. Li X. Yang T. Li N. (2022). Integrated metabolomic and transcriptomic analysis reveals the role of phenylpropanoid biosynthesis pathway in tomato roots during salt stress. Front. Plant Sci. 13, 1023696. 10.3389/fpls.2022.1023696 36570882 PMC9773889

[B133] Jianing G. Yuhong Y. Yijun G. Rasheed A. Qian Z. Zhiming X. (2022). Improvement of heat stress tolerance in soybean (glycine max l.) by using conventional and molecular tools. Front. Plant Sci. 13, 993189. 10.3389/fpls.2022.993189 36226280 PMC9549248

[B134] Jorge T. F. Rodrigues J. A. Caldana C. Schmidt R. Dongen J. T. Thomas‐Oates J. (2016). Mass spectrometry‐based plant metabolomics: Metabolite responses to abiotic stress. Mass Spectrometry Reviews 35, 620–649. 10.1002/mas.21449 25589422

[B135] Joshi J. Hasnain G. Logue T. Lynch M. Wu S. Guan J. C. (2021). A core metabolome response of maize leaves subjected to long-duration abiotic stresses. Metabolites 11, 797. 10.3390/metabo11110797 34822455 PMC8625080

[B136] Kaleem Z. Xu W. Ulhassan Z. Shahbaz H. He D. Naeem S. (2024). Harnessing the potential of copper-based nanoparticles in mitigating abiotic and biotic stresses in crops. Environ. Sci. Pollut. Res. 31, 59727–59748. 10.1007/s11356-024-35174-w 39373837

[B137] Kamal K. Y. Khodaeiaminjan M. Yahya G. El-Tantawy A. A. El-Moneim D. A. El-Esawi M. A. (2021). Modulation of cell cycle progression and chromatin dynamic as tolerance mechanisms to salinity and drought stress in maize. Physiol. Plant. 172, 684–695. 10.1111/ppl.13260 33159351

[B138] Kar S. Leszczynski J. (2017). Recent advances of computational modeling for predicting drug metabolism: a perspective. Curr. Drug Metab. 18, 1106–1122. 10.2174/1389200218666170607102104 28595533

[B139] Kariya K. Mori H. Ueno M. Yoshikawa T. Teraishi M. Yabuta Y. (2024). Identification and evolution of a diterpenoid phytoalexin oryzalactone biosynthetic gene in the genus oryza. Plant J. 118, 358–372. 10.1111/tpj.16608 38194491

[B140] Katam R. Lin C. Grant K. Katam C. S. Chen S. (2022). Advances in plant metabolomics and its applications in stress and single-cell biology. Int. Journal Molecular Sciences 23, 6985. 10.3390/ijms23136985 35805979 PMC9266571

[B141] Kaur R. Aslam L. Kapoor N. Mahajan R. (2020). Identification and comparative expression analysis of chalcone synthase, flavanone 3-hydroxylase and dihydroflavonol 4-reductase genes in wild pomegranate (Punica granatum L.) organs. Braz. J. Bot. 43, 883–896. 10.1007/s40415-020-00648-x

[B142] Kebert M. Kostić S. Vuksanović V. Gavranović Markić A. Kiprovski B. Zorić M. (2022). Metal-and organ-specific response to heavy metal-induced stress mediated by antioxidant enzymes’ activities, polyamines, and plant hormones levels in Populus deltoides. Plants 11 (23), 3246. 10.3390/plants11233246 36501286 PMC9741192

[B145] Khalid F. Rasheed Y. Ashraf H. Asif K. Maqsood M. Shahbaz M. (2025). Nanoparticle-mediated phytohormone interplay: Advancing plant resilience to abiotic stresses. J. Crop Health 77, 56. 10.1007/s10343-025-01121-z

[B146] Khan N. (2025). Exploring plant resilience through secondary metabolite profiling: Advances in stress response and crop improvement. Plant, Cell and Environ. 48, 4823–4837. 10.1111/pce.15473 40091600

[B147] Khan N. Ali S. Shahid M. A. Kharabian-Masouleh A. (2017). Advances in detection of stress tolerance in plants through metabolomics approaches. Plant Omics 10, 153–163. 10.21475/poj.10.03.17.pne600

[B148] Khan A. Salam A. Li G. Iqbal B. Ulhassan Z. Liu Q. (2024). Nanoparticles and their crosstalk with stress mitigators: A novel approach towards abiotic stress tolerance in agricultural systems. Crop J. 12, 1280–1298. 10.1016/j.cj.2024.09.010

[B149] Khare S. Singh N. B. Singh A. Hussain I. Niharika K. M. Yadav V. (2020). Plant secondary metabolites synthesis and their regulations under biotic and abiotic constraints. J. Plant Biol. 63 (3), 203–216. 10.1007/s12374-020-09245-7

[B150] Kholiya S. Rawat A. Auli A. Kurmi A. Tripathi A. Chauhan A. (2026). The effect of growth stages on yield, chemical composition and antimicrobial activity of the essential oil of Ocimum gratissimum var. CIM-Akshay. J. Essent. Oil Res. 38 (1), 109–117. 10.1080/10412905.2025.2604751

[B152] Kivimäenpää M. Virjamo V. Nissinen K. Pikkarainen L. Ghimire R. P. Julkunen-Tiitto R. (2024). Responses of needle terpene concentrations and characteristics of resin canals to different warming treatments in Scots pine and Norway spruce seedlings grown in a field experiment. Can. J. For. Res. 55, 1–9. 10.1139/cjfr-2024-0153

[B153] Klassen A. Faccio A. T. Canuto G. A. B. Cruz P. L. R. Ribeiro H. C. Tavares M. F. M. (2017). Metabolomics: Definitions and significance in systems biology. Adv. Exp. Med. Biol. 965, 3–17. 10.1007/978-3-319-47656-8_1 28132174

[B154] Koley S. Jyoti P. Lingwan M. Allen D. K. (2024). Isotopically nonstationary metabolic flux analysis of plants: recent progress and future opportunities. New Phytol. 242, 1911–1918. 10.1111/nph.19734 38628036

[B155] Koley R. Mandal M. Mondal A. Debnath P. Mondal A. Mondal N. K. (2025). Synthesis of calcium carbonate nanoparticles from mollusc shell waste and its efficacy towards plant growth and development. Sustain. Chem. One World 6, 100056. 10.1016/j.scowo.2025.100056

[B156] Kolodziejczyk-Czepas J. Bijak M. Saluk J. Ponczek M. B. Zbikowska H. M. Nowak P. (2015). Radical scavenging and antioxidant effects of matricaria chamomilla Polyphenolic–Polysaccharide conjugates. Int. J. Biol. Macromol. 72, 1152–1158. 10.1016/j.ijbiomac.2014.09.032 25285848

[B158] Korte P. Unzner A. Damm T. Berger S. Krischke M. Mueller M. J. (2023). High triacylglycerol turnover is required for efficient opening of stomata during heat stress in arabidopsis. Plant J. 115, 81–96. 10.1111/tpj.16210 36976526

[B159] Kotzamani A. Vasilakoglou I. Dhima K. Moulas A. N. Vaiou M. Stefanou S. (2021). Impact of soil salinity on barley allelopathic potential and main secondary metabolites gramine and hordenine. J. Plant Growth Regul. 40, 137–146. 10.1007/s00344-020-10084-6

[B160] Król-Grzymała A. Amarowicz R. (2020). Phenolic compounds of soybean seeds from two European countries and their antioxidant properties. Molecules 25 (9), 2075. 10.3390/molecules25092075 32365546 PMC7249021

[B161] Kumar A. Katiyar J. K. (2025). Micro- and biofluidics: Structure, properties, technology and spotlight into the future. Boca Raton, FL: CRC Press.

[B163] Kumar P. Yadav S. Singh M. P. (2020). Possible involvement of xanthophyll cycle pigments in heat tolerance of chickpea (*Cicer arietinum* L.). Physiology Mol. Biol. Plants 26, 1773–1785. 10.1007/s12298-020-00870-7 32943815 PMC7468050

[B164] Kumar D. Singh R. Upadhyay S. Verma K. Tripathi R. Liu H. (2023). Review on interactions between nanomaterials and phytohormones: Novel perspectives and opportunities for mitigating environmental challenges. Plant Science An International Journal Experimental Plant Biology 340, 111964. 10.1016/j.plantsci.2023.111964 38159611

[B165] Kusvuran S. (2021). Microalgae (chlorella vulgaris beijerinck) alleviates drought stress of broccoli plants by improving nutrient uptake, secondary metabolites, and antioxidative defense system. Hortic. Plant J. 7, 221–231. 10.1016/j.hpj.2021.03.007

[B166] Laddomada B. Blanco A. Mita G. D’Amico L. Singh R. P. Ammar K. (2021). Drought and heat stress impacts on phenolic acids accumulation in durum wheat cultivars. Foods 10, 2142. 10.3390/foods10092142 34574252 PMC8468590

[B167] Lattanzio V. (2013). Phenolic compounds: Introduction 50. arXiv, 1543–1580. 10.1007/978-3-642-22144-6_57

[B168] Lee H. Choi B. Oh S. Park H. Popova E. Paik M. J. (2023). Dynamics of organic acids during the Droplet‐Vitrification cryopreservation procedure can be a signature of oxidative stress in pogostemon yatabeanus. Plants 12, 3489. 10.3390/plants12193489 37836228 PMC10575133

[B169] Leopold I. Günther D. Schmidt J. Neumann D. (1999). Phytochelatins and heavy metal tolerance. Phytochemistry 50 (8), 1323–1328. 10.1016/s0031-9422(98)00347-1

[B170] Lesk C. Anderson W. Rigden A. Coast O. Jägermeyr J. McDermid S. (2022). Compound heat and moisture extreme impacts on global crop yields under climate change. Nat. Rev. Earth and Environ. 3, 872–889. 10.1038/s43017-022-00368-8

[B171] Li C. Zha W. Li W. Wang J. You A. (2023). Advances in the biosynthesis of terpenoids and their ecological functions in plant resistance. Int. J. Mol. Sci. 24, 11561. 10.3390/ijms241411561 37511319 PMC10380271

[B172] Li Y. Su Z. Lin Y. Xu Z. Bao H. Wang F. (2024). Utilizing transcriptomics and metabolomics to unravel key genes and metabolites of maize seedlings in response to drought stress. BMC Plant Biol. 24, 34. 10.1186/s12870-023-04712-y 38185653 PMC10773024

[B173] Li S. Khoso M. A. Xu H. Zhang C. Liu Z. Wagan S. (2024). WRKY transcription factors (TFs) as key regulators of plant resilience to environmental stresses: Current perspective. Agronomy 14, 2421. 10.3390/agronomy14102421

[B174] Limami A. M. Cukier C. Hirel B. (2023). 15N-labelling of leaves combined with GC-MS analysis as a tool for monitoring the dynamics of nitrogen incorporation into amino acids. Springer U. S., 151–161. 10.1007/978-1-0716-3044-0_8 36944877

[B175] Lin Y. Hou J. Li B. Shu W. Wan J. (2025). Advancements in Nanomaterials and Molecular Probes for Spatial Omics. ACS Nano 19, 11604–11624. 10.1021/acsnano.4c18470 40125910

[B176] Liu Q. Luo L. Zheng L. (2018). Lignins: biosynthesis and biological functions in plants. Int. Journal Molecular Sciences 19 (2), 335. 10.3390/ijms19020335 29364145 PMC5855557

[B177] Liu B. Peng X. Han L. Hou L. Li B. (2020). Effects of exogenous spermidine on root metabolism of cucumber seedlings under salt stress by GC–MS. Agronomy 10, 459. 10.3390/agronomy10040459

[B178] Liu X. Yu Y. Huang S. Xu C. Wang X. Gao J. (2022). The impact of drought and heat stress at flowering on maize kernel filling: Insights from the field and laboratory. Agric. For. Meteorology 312, 108733. 10.1016/j.agrformet.2021.108733

[B179] Liu J. Wang J. Zhang T. Li M. Yan H. Liu Q. (2023). Exogenous melatonin positively regulates rice root growth through promoting the antioxidant system and mediating the auxin signaling under Root‐Zone hypoxia stress. Agronomy 13, 386. 10.3390/agronomy13020386

[B180] Liu X. Zhang C. Lamlom S. F. Zhao K. Abdelghany A. M. Wang X. (2024). Genetic adaptations of soybean to cold stress reveal key insights through transcriptomic analysis. Biology 13, 856. 10.3390/biology13110856Liu 39596811 PMC11591561

[B181] López-Hidalgo C. Lamelas L. Cañal M. J. Valledor L. Meijón M. (2023). Untargeted metabolomics revealed essential biochemical rearrangements towards combined heat and drought stress acclimatization in pinus pinaster. Environ. Exp. Bot. 208, 105261. 10.1016/j.envexpbot.2023.105261

[B182] López-Villamor A. Zas R. Pérez A. Cáceres Y. Silva M. N. Vasconcelos M. (2021). Traumatic resin ducts induced by methyl jasmonate in pinus spp. Trees 35, 557–567. 10.1007/s00468-020-02057-9

[B183] Loreto F. Schnitzler J. P. (2010). Abiotic stresses and induced BVOCs. Trends Plant Sci. 15, 154–166. 10.1016/j.tplants.2009.12.006 20133178

[B184] Lucini L. Borgognone D. Rouphael Y. Cardarelli M. Bernardi J. Colla G. (2016). Mild potassium chloride stress alters the mineral composition, hormone network, and phenolic profile in artichoke leaves. Front. Plant Sci. 7, 948. 10.3389/fpls.2016.00948 27446175 PMC4923119

[B185] Lv Z. Y. Sun W. J. Jiang R. Chen J. F. Ying X. Zhang L. (2021). Phytohormones Jasmonic Acid, Salicylic Acid, Gibberellins, and Abscisic Acid Are Key Mediators of Plant Secondary Metabolites. World J. Traditional Chin. Med. 7, 307–325. 10.4103/wjtcm.wjtcm_20_21

[B186] Ma X. Fernández F. M. (2024). Advances in mass spectrometry imaging for spatial cancer metabolomics. Mass Spectrometry Reviews 43, 235–268. 10.1002/mas.21804 36065601 PMC9986357

[B187] Ma D. Reichelt M. Yoshida K. Gershenzon J. Constabel C. P. (2018). Two R2R3-MYB proteins are broad repressors of flavonoid and phenylpropanoid metabolism in poplar. Plant J. 96, 949–965. 10.1111/tpj.14081 30176084

[B188] Ma Q. Wang H. Wu E. Zhang H. Feng Y. Feng B. (2023). Widely targeted metabolomic analysis revealed the effects of alkaline stress on nonvolatile and volatile metabolites in broomcorn millet grains. Food Res. Int. 171, 113066. 10.1016/j.foodres.2023.113066 37330826

[B190] Magwanga R. O. Lu P. Kirungu J. N. Cai X. Zhou Z. Wang X. (2018). Whole genome analysis of cyclin dependent kinase (CDK) gene family in cotton and functional evaluation of the role of CDKF4 gene in drought and salt stress tolerance in plants. Int. J. Mol. Sci. 19, 2625. 10.3390/ijms19092625 30189594 PMC6164816

[B191] Mahmood U. Li X. Fan Y. Chang W. Niu Y. Li J. (2022). Multi-omics revolution to promote plant breeding efficiency. Front. Plant Sci. 7, 62. 10.3389/fpls.2022.1062952 PMC9773847 36570904

[B192] Majumdar S. Keller A. A. (2021). Omics to address the opportunities and challenges of nanotechnology in agriculture. Crit. Rev Environ Sci. Technol 51, 2595–2636. 10.1080/10643389.2020.1785264

[B194] Malone S. C. Simonpietri A. Knighton W. B. Trowbridge A. M. (2023). Drought impairs herbivore-induced volatile terpene emissions by ponderosa pine but not through constraints on newly assimilated carbon. Tree Physiol. 43, 938–951. 10.1093/treephys/tpad016 36762917

[B195] Manasa S. L. Panigrahy M. Panigrahi K. C. Rout G. R. (2022). Overview of cold stress regulation in plants. Botanical Rev. 88 (3), 359–387. 10.1007/s12229-021-09267-x

[B197] Marček T. Hamow K. A. Végh B. Janda T. Darko E. (2019). Metabolic response to drought in six winter wheat genotypes. PLoS One 14, 0212411. 10.1371/journal.pone.0212411 30779775 PMC6380608

[B198] Martin X. Sodaei R. Santpere G. (2021). Mechanisms of binding specificity among bHLH transcription factors. Int. J. Mol. Sci. 22, 9150. 10.3390/ijms22179150 34502060 PMC8431614

[B199] Meng L. Song W. Chen S. Hu F. Pang B. Cheng J. (2022). Widely targeted metabolomics analysis reveals the mechanism of quality improvement of flue-cured tobacco. Front. Plant Sci. 13, 1074029. 10.3389/fpls.2022.1074029 36523627 PMC9746875

[B200] Mishra J. Singh R. Arora N. K. (2017). Alleviation of heavy metal stress in plants and remediation of soil by rhizosphere microorganisms. Front. Microbiology 8, 1706. 10.3389/fmicb.2017.01706 28932218 PMC5592232

[B203] Mishra B. Bansal S. Tripathi S. Mishra S. Yadav R. K. Sangwan N. S. (2024). Differential regulation of key triterpene synthase gene under abiotic stress in withania somnifera and its co-relation to sterols and withanolides. Plant Physiology Biochem. 208, 108419. 10.1016/j.plaphy.2024.108419 38377888

[B205] Moellering E. R. Benning C. (2011). Galactoglycerolipid metabolism under stress: a time for remodeling. Trends Plant Science 16 (2), 98–107. 10.1016/j.tplants.2010.11.004 21145779

[B206] Msomi M. N. Prinsloo G. Nogemane N. (2024). 1H-NMR-Based metabolomics profiling and proteomic analysis of soybean (glycine max l.) in response to dicarboxylic acids (photon) application as a stress priming agent. Heliyon 10. 10.1016/j.heliyon.2024.e37466 PMC11414495 39309962

[B208] Mushtaq N. U. Saleem S. Rasool A. Shah W. H. Tahir I. Seth C. S. (2025). Proline tagging for stress tolerance in plants. Int. J. Genomics 2025 (1), 9348557. 10.1155/ijog/9348557 40207093 PMC11981710

[B209] Mustafavi S. H. Shekari F. Maleki H. H. (2016). Influence of exogenous polyamines on antioxidant defence and essential oil production in valerian (Valeriana officinalis L.) plants under drought stress. Acta Agriculturae Slov. 107 (1), 81–91. 10.14720/aas.2016.107.1.09

[B210] Muthusamy M. Lee S. I. (2024). Abiotic stress-induced secondary metabolite production in brassica: Opportunities and challenges. Front. Plant Sci. 14, 1323085. 10.3389/fpls.2023.1323085 38239210 PMC10794482

[B211] Naik B. Kumar V. Rizwanuddin S. Chauhan M. Choudhary M. Gupta A. K. (2023). Genomics, proteomics, and metabolomics approaches to improve abiotic stress tolerance in tomato plant. Int. J. Mol. Sci. 24 (3), 3025. 10.3390/ijms24033025 36769343 PMC9918255

[B212] Naliwajski M. Skłodowska M. (2021). The relationship between the antioxidant system and proline metabolism in the leaves of cucumber plants acclimated to salt stress. Cells 10, 609. 10.3390/cells10030609 33801884 PMC7998282

[B213] Nam K. H. Kim D. Y. Kim H. J. Pack I. S. Kim H. J. Chung Y. S. (2019). Global metabolite profiling based on GC–MS and LC–MS/MS analyses in ABF3-Overexpressing soybean with enhanced drought tolerance. Appl. Biol. Chem. 62, 1–9. 10.1186/s13765-019-0425-5

[B214] Nand S. Neeraj A. Patel A. Shukla S. Srivastava P. K. (2025). “Plant and Environment Interaction: Special Reference to Phytoremediation of Heavy Metals Using Ricinus communis L,” in Ricinus Communis: A Climate Resilient Commercial Crop for Sustainable Environment (Singapore: Springer Nature Singapore), 1–18.

[B215] Naresh V. Lee N. (2021). A review on biosensors and recent development of nanostructured materials-enabled biosensors. Sensors 21, 1109. 10.3390/s21041109 33562639 PMC7915135

[B216] Naz S. Vallejo M. García A. Barbas C. (2014). Method validation strategies involved in non-targeted metabolomics. J. Chromatogr. A 1353, 99–105. 10.1016/j.chroma.2014.04.071 24811151

[B217] Nikalje G. C. Kumar J. Nikam T. D. Suprasanna P. (2019). FT-IR profiling reveals differential response of roots and leaves to salt stress in a halophyte sesuvium portulacastrum (l.) l. Biotechnol. Rep. 23, 00352. 10.1016/j.btre.2019.e00352 31293906 PMC6595135

[B218] Ninkuu V. Zhang L. Yan J. Fu Z. Yang T. Zeng H. (2021). Biochemistry of terpenes and recent advances in plant protection. Int. J. Mol. Sci. 22, 5710. 10.3390/ijms22115710 34071919 PMC8199371

[B219] Nishanth M. J. Sheshadri S. A. Rathore S. S. Srinidhi S. Simon B. (2018). Expression analysis of cell wall invertase under abiotic stress conditions influencing specialized metabolism in catharanthus roseus. Sci. Rep. 8, 15059. 10.1038/s41598-018-33415-w 30305670 PMC6180051

[B220] Noman A. Ali Q. Naseem J. Javed M. T. Kanwal H. Islam W. (2018). Sugar beet extract acts as a natural bio-stimulant for physio-biochemical attributes in water stressed wheat (*Triticum aestivum* L.). Acta Physiol. Plant. 40 (6), 110. 10.1007/s11738-018-2681-0

[B221] Noushahi H. A. Khan A. H. Noushahi U. F. Hussain M. Javed T. Zafar M. (2022). Biosynthetic pathways of triterpenoids and strategies to improve their biosynthetic efficiency. Plant Growth Regul. 97 (3), 439–454. 10.1007/s10725-022-00818-9 35382096 PMC8969394

[B222] Ohnishi M. Anegawa A. Sugiyama Y. Harada K. Oikawa A. Nakayama Y. (2018). Molecular components of arabidopsis intact vacuoles clarified with metabolomic and proteomic analyses. Plant Cell Physiology 59, 1353–1362. 10.1093/pcp/pcy069 29660082

[B223] Okazaki Y. Saito K. (2014). Roles of lipids as signaling molecules and mitigators during stress response in plants. Plant J. 79 (4), 584–596. 10.1111/tpj.12556 24844563

[B224] Olanrewaju O. S. Glick B. R. Babalola O. O. (2024). Metabolomics-guided utilization of beneficial microbes for climate-resilient crops. Curr. Opin. Chem. Biol. 79, 102427. 10.1016/j.cbpa.2024.102427 38290195

[B225] Padmanabhan D. Manimekalai R. Senthil-Nathan S. Suganthi M. Palanisamy S. (2025). Biosynthesis, therapeutic characteristics, origin and strategies to improve the yield of vasicine in plants. Vegetos, 1–15. 10.1007/s42535-025-01161-w

[B226] Pan Q. Wang C. Xiong Z. Wang H. Fu X. Shen Q. (2019). CrERF5, an AP2/ERF transcription factor, positively regulates the biosynthesis of bisindole alkaloids and their precursors in *Catharanthus roseus* . Front. Plant Sci. 10, 931. 10.3389/fpls.2019.00931 31379908 PMC6657538

[B227] Panda A. Rangani J. Parida A. K. (2021). Unravelling salt responsive metabolites and metabolic pathways using non-targeted metabolomics approach and elucidation of salt tolerance mechanisms in the xero-halophyte *Haloxylon salicornicum* . Plant Physiology Biochem. 158, 284–296. 10.1016/j.plaphy.2020.11.012 33239222

[B229] Papafotiou M. Martini A. N. Papanikolaou E. Stylias E. G. Kalantzis A. (2021). Hybrids development between greek salvia species and their drought resistance evaluation under attapulgite-amended substrate. Agronomy 11, 2401. 10.3390/agronomy11122401

[B231] Pavel M. Cretu L. Negrila C. Culiță D. Vasile A. State R. (2025). Mono-(ni, au) and bimetallic (ni-au) nanoparticles-loaded ZnAlO mixed oxides as sunlight-driven photocatalysts for environmental remediation. Molecules 30, 3249. 10.3390/molecules30153249 40807424 PMC12348654

[B232] Perez De Souza L. Alseekh S. Brotman Y. Fernie A. R. (2020). Network-based strategies in metabolomics data analysis and interpretation: from molecular networking to biological interpretation. Expert Rev. Proteomics 17 (4), 243–255. 10.1080/14789450.2020.1766975 32380880

[B233] Petrova B. Guler A. T. (2024). Recent developments in single-cell metabolomics by mass Spectrometry─ A perspective. J. Proteome Res. 24, 1493–1518. 10.1021/acs.jproteome.4c00646 39437423 PMC11976873

[B234] Ponce-Rodríguez H. D. Verdú-Andrés J. Herráez-Hernández R. Campíns-Falcó P. (2020). Innovations in extractive phases for in-tube solid-phase microextraction coupled to miniaturized liquid chromatography: A critical review. Molecules 25, 2460. 10.3390/molecules25102460 32466305 PMC7287690

[B235] Pottosin I. Velarde-Buendía A. M. Bose J. Fuglsang A. T. Shabala S. (2014). Polyamines cause plasma membrane depolarization, activate Ca2+-and modulate H+-ATPase pump activity in pea roots. J. Exp. Bot. 65 (9), 2463–2472. 10.1093/jxb/eru133 24723394

[B236] Powers R. Andersson E. R. Bayless A. L. Brua R. B. Chang M. C. Cheng L. L. (2024). Best practices in NMR metabolomics: Current state. TrAC Trends Anal. Chem. 171, 117478. 10.1016/j.trac.2023.117478 PMC11999570 40237011

[B238] Qaderi M. M. Martel A. B. Strugnell C. A. (2023). Environmental factors regulate plant secondary metabolites. Plants 12, 447. 10.3390/plants12030447 36771531 PMC9920071

[B239] Qian Z. He L. Li F. (2024). Understanding cold stress response mechanisms in plants: An overview. Front. Plant Sci. 15, 1443317. 10.3389/fpls.2024.1443317 39568458 PMC11576170

[B240] Qin J. Zhao C. Wang S. Gao N. Wang X. Na X. (2022). PIF4-PAP1 interaction affects MYB-bHLH-WD40 complex formation and anthocyanin accumulation in arabidopsis. J. Plant Physiology 268, 153558. 10.1016/j.jplph.2021.153558 34798465

[B241] Qu M. Chen G. Bunce J. A. Zhu X. Sicher R. C. (2018). Systematic biology analysis on photosynthetic carbon metabolism of maize leaf following sudden heat shock under elevated CO2. Sci. Rep. 8, 7849. 10.1038/s41598-018-26283-x 29777170 PMC5959914

[B242] Rabeh K. Hnini M. Oubohssaine M. (2025). A comprehensive review of transcription factor-mediated regulation of secondary metabolites in plants under environmental stress. Stress Biol. 5, 15. 10.1007/s44154-024-00201-w

[B243] Rahman M. A. Song Y. Hasan M. M. Jahan M. S. Siddiqui M. H. Park H. S. (2024). Mechanistic Basis of Silicon-Mediated Cold Stress Tolerance in Alfalfa (*Medicago sativa* L.). Silicon 16, 1057–1069. 10.1007/s12633-023-02697-9

[B244] Rahnama A. Salehi F. Meskarbashee M. Khanlou K. M. Ghorbanpour M. Harrison M. T. (2024). High temperature perturbs physicochemical parameters and fatty acids composition of safflower (*Carthamus tinctorius* L.). BMC Plant Biol. 24, 1080. 10.1186/s12870-024-05781-3 39543469 PMC11566086

[B246] Rainio M. J. Margus A. Virtanen V. Lindström L. Salminen J. P. Saikkonen K. (2020). Glyphosate-based herbicide has soil-mediated effects on potato glycoalkaloids and oxidative status of a potato pest. Chemosphere 258, 127254. 10.1016/j.chemosphere.2020.127254 32559492

[B247] Raja M. M. Vijayalakshmi G. Naik M. L. Basha P. O. Sergeant K. Hausman J. F. (2019). Pollen development and function under heat stress: from effects to responses. Acta Physiol. Plant. 41, 47. 10.1007/s11738-019-2835-8

[B249] Rajput V. Minkina T. Kumari A. H. Singh V. Verma K. Mandzhieva S. (2021). Coping with the challenges of abiotic stress in plants: New dimensions in the field application of nanoparticles. Plants 10. 1221. 10.3390/plants10061221 34203954 PMC8232821

[B419] Ramakrishna R. (2021). Dual role of phenolic bioactives in improving functional health benefits and abiotic stress resilience in barley (Doctoral dissertation, North Dakota State University).

[B250] Rao M. J. Duan M. Zhou C. Jiao J. Cheng P. Yang L. (2025). Antioxidant defense system in plants: reactive oxygen species production, signaling, and scavenging during abiotic stress-induced oxidative damage. Horticulturae 11 (5), 477. 10.3390/horticulturae11050477

[B251] Rasmus P. Kozłowska E. (2023). Antioxidant and anti-inflammatory effects of carotenoids in mood disorders: An overview. Antioxidants 12, 676. 10.3390/antiox12030676 36978923 PMC10045512

[B254] Raza A. Charagh S. Najafi-Kakavand S. Abbas S. Shoaib Y. Anwar S. (2023). Role of phytohormones in regulating cold stress tolerance: Physiological and molecular approaches. Plant Stress 8, 100152. 10.1016/j.stress.2023.100152

[B256] Rehman A. Khan S. Sun F. Peng Z. Feng K. Wang N. (2024). Exploring the nano-wonders: unveiling the role of Nanoparticles in enhancing salinity and drought tolerance in plants. Front. Plant Sci. 14, 1324176. 10.3389/fpls.2023.1324176 38304455 PMC10831664

[B257] Ren J. L. Zhang A. H. Kong L. Wang X. J. (2018). Advances in mass spectrometry-based metabolomics for investigation of metabolites. RSC Advances 8 (40), 22335–22350. 10.1039/c8ra01574k 35539746 PMC9081429

[B258] Ren S. Y L. D Z. W W. X C. X. Wan W. (2025). TcMYB73, a salicylic acid-responsive R2R3-MYB transcription factor, positively regulates paclitaxel biosynthesis in Taxus chinensis in direct and indirect ways. BMC Plant Biol. 25, 723. 10.1186/s12870-025-06755-9 40437362 PMC12117831

[B259] Renzetti M. Bertolini E. Trovato M. (2024). Proline metabolism genes in transgenic plants: meta-analysis under drought and salt stress. Plants 13, 1913. 10.3390/plants13141913 39065440 PMC11280441

[B260] Rocha Júnior D. S. Barbosa A. C. O. Batista I. A. Camillo L. R. Lopes N. S. Costa M. G. (2023). Impact of moderate water deficit at the fruit development stage of tomato (Solanum lycopersicum L.): effects on plant growth, physiology, fruit yield and quality and expression of carotenoid biosynthesis genes. Acta Physiol. Plant. 45 (5), 65. 10.1007/s11738-023-03549-0

[B261] Rosenkranz M. Chen Y. Zhu P. Vlot A. C. (2021). Volatile Terpenes—Mediators of plant-to-plant communication. Plant J. 108, 617–631. 10.1111/tpj.15453 34369010

[B262] Rosier A. Medeiros F. H. Bais H. P. (2018). Defining plant growth promoting rhizobacteria molecular and biochemical networks in beneficial plant-microbe interactions. Plant Soil 428 (1), 35–55. 10.1007/s11104-018-3679-5

[B263] Roychowdhury R. Das S. P. Gupta A. Parihar P. Chandrasekhar K. Sarker U. (2023). Multi-omics pipeline and omics-integration approach to decipher plant’s abiotic stress tolerance responses. Genes 14, 1281. 10.3390/genes14061281 37372461 PMC10298225

[B265] Sabir F. Sangwan R. S. Kumar R. Sangwan N. S. (2012). Salt stress-induced responses in growth and metabolism in callus cultures and differentiating *in vitro* shoots of Indian ginseng (Withania somnifera Dunal). J. Plant Growth Regulation 31 (4), 537–548. 10.1007/s00344-012-9264-x

[B266] Sachdev S. Ansari S. A. Ansari M. I. Fujita M. Hasanuzzaman M. (2021). Abiotic stress and reactive oxygen species: Generation, signaling, and defense mechanisms. Antioxidants 10, 277. 10.3390/antiox10020277 33670123 PMC7916865

[B267] Saidi I. Guesmi F. Kharbech O. Hfaiedh N. Djebali W. (2021). Gallic acid improves the antioxidant ability against cadmium toxicity: Impact on leaf lipid composition of sunflower (Helianthus annuus) seedlings. Ecotoxicol. Environ. Saf. 210, 111906. 10.1016/j.ecoenv.2021.111906 33429318

[B268] Saini N. Modolo L. V. Deswal R. Sehrawat A. Yadav N. Ns S. (2024). Expanding roles of crosstalk between hydrogen sulfide and nitric oxide under abiotic stress in plants. Plant Physiology Biochem. 214, 108852. 10.1016/j.plaphy.2024.108852 38943878

[B450] Saini N. Kumari S. Kumari H. Kumar A. Sangwan N. S. (2025). Metabolomics: towards understanding. Plant-Microbiome Interactions for Climate-Resilient Agriculture, 229. 10.1007/978-981-96-3534-4_11

[B269] Salam U. Ullah S. Tang Z. H. Elateeq A. A. Khan Y. Khan J. (2023). Plant metabolomics: an overview of the role of primary and secondary metabolites against different environmental stress factors. Life 13 (3), 706. 10.3390/life13030706 36983860 PMC10051737

[B270] Saleem M. H. Wang X. Parveen A. Perveen S. Mehmood S. Fiaz S. (2022). Alleviation of drought stress by root-applied thiourea is related to elevated photosynthetic pigments, osmoprotectants, antioxidant enzymes, and tubers yield and suppressed oxidative stress in potatoes cultivars. PeerJ 10, e13121. 10.7717/peerj.13121 35415014 PMC8995019

[B272] Sanches A. G. Ferreira Feitosa E. M. Schwob A. C. (2024). Metabolic response of vitamin C and phenolic compounds in fresh‐cut pineapple induced by pulsed electric fields. Future Postharvest Food 1 (4), 454–465. 10.1002/fpf2.12045

[B273] Sánchez-Ramos M. Berman-Bahena S. Alvarez L. Sánchez-Carranza J. N. Bernabé-Antonio A. Román-Guerrero A. (2022). Effect of plant growth regulators on different explants of *Artemisia ludoviciana* and their influence on achillin production. Processes 10, 1439. 10.3390/pr10081439

[B275] Sangwan N. S. Abad Farooqi A. H. Singh Sangwan R. (1994). Effect of drought stress on growth and essential oil metabolism in lemongrasses. New Phytol. 128 (1), 173–179. 10.1111/j.14698137.1994.tb04000.x 33874545

[B276] Sangwan R. S. Chaurasiya N. D. Lal P. Misra L. Tuli R. Sangwan N. S. (2008). Withanolide a is inherently de novo biosynthesized in roots of the medicinal plant ashwagandha (*Withania somnifera*). Physiol. Plant. 133, 278–287. 10.1111/j.1399-3054.2008.01076.x 18312497

[B277] Sangwan R. S. Tripathi S. Singh J. Narnoliya L. K. Sangwan N. S. (2013). De novo sequencing and assembly of centella asiatica leaf transcriptome for mapping structural, functional and regulatory genes with reference to secondary metabolism. Gene 525, 58–76. 10.1016/j.gene.2013.04.057 23644021

[B278] Sangwan N. S. Jha S. Mitra A. (2024). Unlocking nature’s treasure trove: Biosynthesis and elicitation of secondary metabolites from plants. Plant Growth Regul. 104, 1–4. 10.1007/s10725-024-01184-4

[B279] Sarmoum R. Haid S. Biche M. Djazouli Z. Zebib B. Merah O. (2019). Effect of salinity and water stress on the essential oil components of rosemary (*Rosmarinus officinalis* L.). Agronomy 9, 214. 10.3390/agronomy9050214

[B280] Satrio R. D. Fendiyanto M. H. Miftahudin M. (2024). “Tools and techniques used at global scale through genomics, transcriptomics, proteomics, and metabolomics to investigate plant stress responses at the molecular level,” in Molecular dynamics of plant stress and its management (Singapore: Springer Nature Singapore), 555–607.

[B281] Seleiman M. F. Al-Suhaibani N. Ali N. Akmal M. Alotaibi M. Refay Y. (2021). Drought stress impacts on plants and different approaches to alleviate its adverse effects. Plants 10 (2), 259. 10.3390/plants10020259 33525688 PMC7911879

[B282] Selwal N. Rahayu F. Herwati A. Latifah E. Suhara C. Suastika I. B. K. (2023). Enhancing secondary metabolite production in plants: Exploring traditional and modern strategies. J. Agriculture Food Research 14, 100702. 10.1016/j.jafr.2023.100702

[B283] Semmar N. (2024). Secondary Metabolites in Plant Stress Adaptation. Springer Nature.

[B284] Sen B. Roy D. Narayan M. Sarma H. (2025). Nanoparticle-driven stress alleviation: exploring the roles of metal and metal oxide nanoparticles in plant abiotic stress management. Discov. Plants 2, 74. 10.1007/s44372-025-00153-z

[B416] Shafi A. Zahoor I. Mushtaq U. (2019). “Proline accumulation and oxidative stress: diverse roles and mechanism of tolerance and adaptation under salinity stress,” in Salt Stress, Microbes, and Plant Interactions: Mechanisms and Molecular Approaches. (Singapore: Springer Singapore) 2, 269–300. 10.1007/978-981-13-8805-7_13

[B285] Shaghaleh H. Hamoud Y. Saleem M. Elansary H. Mahmoud E. Sheteiwy M. (2025). Metallic oxide nanoparticles enhance lead stress tolerance in Pearl millet (Pennisetum glaucum L.) by improving morpho–physio–biochemical traits. Sci. Rep. 15. 10.1038/s41598-025-11722-3 PMC12297504 40715277

[B286] Shahzad A. Ullah S. Dar A. A. Sardar M. F. Mehmood T. Tufail M. A. (2021). Nexus on climate change: Agriculture and possible solution to cope future climate change stresses. Environ. Sci. Pollut. Res. 28 (12), 14211–14232. 10.1007/s11356-021-12649-8 33515149

[B287] Shan Y. Jin S. (2025). Biosynthetic machinery to abiotic stress-driven emission: Decoding multilayer regulation of volatile terpenoids in plants. Antioxidants 14, 673. 10.3390/antiox14060673 40563307 PMC12190140

[B288] Shang J. Feng D. Liu H. Niu L. Li R. Li Y. (2024). Evolution of the biosynthetic pathways of terpene scent compounds in roses. Curr. Biol. 34, 3550–3563. 10.1016/j.cub.2024.06.075 39043188

[B289] Shao S. Lv B. Wang M. Zeng S. Wang S. Yang Z. (2025). Biosynthesis and regulatory mechanism of tanshinones and phenolic acids in Salvia miltiorrhiza. Plant J. 123, 70358. 10.1111/tpj.70358 40720694

[B290] Sharma S. Dhadly D. K. (2023). Nano-inspired biosensors and plant diseases: recent advances and challenges. Nanoparticles Plant-Microbe Interact., 135–162. 10.1016/B978-0-323-90619-7.00002-3

[B291] Sharma H. Sharma P. Kumar A. Chawla N. Dhatt A. S. (2024). Multifaceted regulation of anthocyanin biosynthesis in plants: a comprehensive review. J. Plant Growth Regul. 43, 3048–3062. 10.1007/s00344-024-11306-x

[B292] Sharma N. Negi N. Kaur H. Sharma V. Kumari V. Thakur N. (2025). Metabolic adaptations in plants: navigating heavy metal stress for sustainable plant growth. Discov. Appl. Sci. 7 (10), 1041. 10.1007/s42452-025-06715-w

[B293] Shelyakin M. Malyshev R. Silina E. Zakhozhiy I. Golovko T. (2022). UV-B induced changes in respiration and antioxidant enzyme activity in the foliose lichen (*Peltigera aphthosa* L.) willd. Acta Physiol. Plant. 10.1007/s11738-022-03457-9

[B294] Shen C. Ma F. Gao S. Zhou J. Huang D. Huang R. (2025). Concentration-dependent bioactivity profiles of hesperetin and naringenin: insights into antibacterial, antioxidant, and anti-inflammatory effects. ACS Food Sci. and Technol. 5 (2), 492–501. 10.1021/acsfoodscitech.4c00538

[B295] Shiade S. R. G. Zand-Silakhoor A. Fathi A. Rahimi R. Minkina T. Rajput V. D. (2024). Plant metabolites and signaling pathways in response to biotic and abiotic stresses: Exploring bio stimulant applications. Plant Stress 12, 100454. 10.1016/j.stress.2024.100454

[B296] Shrestha A. Khan A. Dey N. (2018). Cis–trans engineering: advances and perspectives on customized transcriptional regulation in plants. Mol. Plant 11, 886–898. 10.1016/j.molp.2018.05.008 29859265

[B297] Siddique A. Kandpal G. Kumar P. (2018). Proline accumulation and its defensive role under diverse stress condition in plants: An overview. J. Pure Appl. Microbiol. 12, 1655–1659. 10.22207/jpam.12.3.73

[B298] Siemińska-Kuczer A. Szymańska-Chargot M. Zdunek A. (2022). Recent advances in interactions between polyphenols and plant cell wall polysaccharides as studied using an adsorption technique. Food Chem. 373, 131487. 10.1016/j.foodchem.2021.131487 34741970

[B299] Silva S. Dias M. C. Pinto D. C. Silva A. M. (2023). Metabolomics as a tool to understand nano-plant interactions: The case study of metal-based nanoparticles. Plants 12, 491. 10.3390/plants12030491 36771576 PMC9921902

[B300] Singh H. (2024). Exploring adaptive modulation in plant functional traits and its impact on the productivity of acacia auriculiformis under CO2-Enriched environment. Industrial Crops Prod. 210, 118186. 10.1016/j.indcrop.2024.118186

[B301] Singh A. Roychoudhury A. (2023). Abscisic acid in plants under abiotic stress: crosstalk with major phytohormones. Plant Cell Reports 42 (6), 961–974. 10.1007/s00299-023-03013-w 37079058

[B302] Singh K. B. Malhotra R. S. Halila M. H. Knights E. J. Verma M. M. (1993). Current status and future strategy in breeding chickpea for resistance to biotic and abiotic stresses. Euphytica 73 (1), 137–149. 10.1007/bf00027190

[B303] Singh A. K. Dhanapal S. Yadav B. S. (2020). The dynamic responses of plant physiology and metabolism during environmental stress progression. Mol. Biol. Rep. 47, 1459–1470. 10.1007/s11033-019-05198-4 31823123

[B304] Singh P. Arif Y. Siddiqui H. Sami F. Zaidi R. Azam A. (2021). Nanoparticles enhances the salinity toxicity tolerance in *Linum usitatissimum* L. by modulating the antioxidative enzymes, photosynthetic efficiency, redox status and cellular damage. Ecotoxicol. Environmental Safety 213, 112020. 10.1016/j.ecoenv.2021.112020 33592373

[B305] Singh A. A. Ghosh A. Agrawal M. Agrawal S. B. (2023). Secondary metabolites responses of plants exposed to ozone: An update. Environ. Sci. Pollut. Res. 30, 88281–88312. 10.1007/s11356-023-28634-2 37440135

[B306] Sobrino-Plata J. Cano F. J. Aranda I. Simón M. B. F. Rodríguez-Calcerrada J. (2024). The impact of drought on plant metabolism in quercus Species—From initial response to recovery. arXiv, 267–313.

[B307] Song C. Cao Y. Dai J. Li G. Manzoor M. A. Chen C. (2022). The multifaceted roles of MYC2 in plants: toward transcriptional reprogramming and stress tolerance by jasmonate signaling. Front. Plant Sci. 13, 868874. 10.3389/fpls.2022.868874 35548315 PMC9082941

[B308] Song Y. Zheng C. Basnet R. Li S. Chen J. Jiang M. (2022). Astaxanthin-synthesized gold nanoparticles enhance salt stress tolerance in rice by enhancing tetrapyrrole biosynthesis and scavenging reactive oxygen species *in vitro* . Plant Stress 6, 100120. 10.1016/j.stress.2022.100120

[B309] Souza L. Fernie A. R. (2024). Computational methods for processing and interpreting mass spectrometry-based metabolomics. Essays Biochemistry 68, 5–13. 10.1042/EBC20230019 PMC11065554 37999335

[B310] Souza V. F. Robin M. Rasulov B. Talts E. Alves E. G. Almutairi B. O. (2025). High temperature acclimation of isoprene emission in date palm is associated with enhanced substrate availability and reduction in synthase activity. Physiol. Plant. 177, 70256. 10.1111/ppl.70256 40325600

[B311] Spicher L. Glauser G. Kessler F. (2016). Lipid antioxidant and galactolipid remodeling under temperature stress in tomato plants. Front. Plant Sci. 7, 167. 10.3389/fpls.2016.00167 26925083 PMC4756161

[B312] Srikanth P. Maxton A. Masih S. A. Sofo A. Khan N. A. (2024). Isoprene: An antioxidant to guard plants against stress. Int J. Plant Biol. 15, 161–174. 10.3390/ijpb15010013

[B313] Srivastava Y. Sangwan N. S. (2020). “Improving medicinal crops through phytochemical perspective: Withania somnifera (Ashwagandha),” in Advancement in crop improvement techniques (Cambridge, United Kingdom: Woodhead Publishing), 275–295.

[B316] Srivastava Y. Tripathi S. Mishra B. Sangwan N. S. (2022). Cloning and homologous characterization of geranylgeranyl pyrophosphate synthase (GGPPS) from Withania somnifera revealed alterations in metabolic flux towards gibberellic acid biosynthesis. Planta 256 (1), 4. 10.1007/s00425-022-03912-4 35648276

[B317] Subramanian I. Verma S. Kumar S. Jere A. Anamika K. (2020). Multi-omics data integration, interpretation, and its application. Bioinforma. Biology Insights 14, 1177932219899051. 10.1177/1177932219899051 32076369 PMC7003173

[B318] Sudiro C. Guglielmi F. Hochart M. Senizza B. Zhang L. Lucini L. (2022). A phenomics and metabolomics investigation on the modulation of drought stress by a biostimulant plant extract in tomato (*Solanum lycopersicum*). Agronomy 12, 764. 10.3390/agronomy12040764

[B319] Sun T. Rao S. Zhou X. Li L. (2022). Plant carotenoids: Recent advances and future perspectives. Mol. Hortic. 2, 3. 10.1186/s43897-022-00023-2 37789426 PMC10515021

[B320] Sun L. Wang J. Cui Y. Cui R. Kang R. Zhang Y. (2023). Changes in terpene biosynthesis and submergence tolerance in cotton. BMC Plant Biol. 23, 330. 10.1186/s12870-023-04334-4 37344795 PMC10283293

[B321] Sun Q. Liu Z. Wang X. Yan A. Ren J. Wang H. (2025). Whole-genome identification and functional analysis of grape bHLH transcription factors: new insights into the regulation of monoterpene biosynthesis. Front. Plant Sci. 16, 1653650. 10.3389/fpls.2025.1653650 41036401 PMC12479481

[B322] Taylor M. J. Lukowski J. K. Anderton C. R. (2021). Spatially resolved mass spectrometry at the single-cell: recent innovations in proteomics and metabolomics. J. Am. Soc. Mass Spectrom. 32, 872–894. 10.1021/jasms.0c00439 33656885 PMC8033567

[B323] Testerink C. Munnik T. (2011). Molecular, cellular, and physiological responses to phosphatidic acid formation in plants. J. Experimental Botany 62 (7), 2349–2361. 10.1093/jxb/err079 21430291

[B324] Thabet S. Alqudah A. (2024). Unraveling the role of nanoparticles in improving plant resilience under environmental stress condition. Plant Soil 503, 313–330. 10.1007/s11104-024-06581-2

[B325] Thakur P. Nayyar H. (2012). “Facing the cold stress by plants in the changing environment: sensing, signaling, and defending mechanisms,” in Plant acclimation to environmental stress (New York, NY: Springer New York), 29–69.

[B326] Thakur M. Sharma S. (2024). “Endophytic secondary metabolites, a novel source for antiviral,” in Promising Antiviral Herbal and Medicinal Plants (Boca Raton, FL: CRC Press), 51–61.

[B327] Tholl D. Rebholz Z. Morozov A. V. O’Maille P. E. (2023). Terpene synthases and pathways in animals: Enzymology and structural evolution in the biosynthesis of volatile infochemicals. Nat. Product. Rep. 40, 766–793. 10.1039/d2np00076h 36880348

[B328] Tienda-Parrilla M. López-Hidalgo C. Guerrero-Sanchez V. M. Infantes-González Á. Valderrama-Fernández R. Castillejo M. Á. (2022). Untargeted MS-Based metabolomics analysis of the responses to drought stress in quercus ilex l. Leaf seedlings. Forests 13, 551. 10.3390/f13040551

[B329] Tinte M. M. Chele K. H. van Der Hooft J. J. Tugizimana F. (2021). Metabolomics-guided elucidation of plant abiotic stress responses in the 4IR era: An Overview. Metabolites 11 (7), 445. 10.3390/metabo11070445 34357339 PMC8305945

[B330] Tiwari Y. K. (2024). Proline as a key player in heat stress tolerance: Insights from maize. Discov. Agric. 2, 121. 10.1007/s44279-024-00084-5

[B331] Toscano S. Trivellini A. Cocetta G. Bulgari R. Francini A. Romano D. (2019). Effect of preharvest abiotic stresses on the accumulation of bioactive compounds in horticultural produce. Front. Plant Sci. 10, 1212. 10.3389/fpls.2019.01212 31636647 PMC6788460

[B333] Tripathi S. Jadaun J. S. Chandra M. Sangwan N. S. (2016). Medicinal plant transcriptomes: the new gateways for accelerated understanding of plant secondary metabolism. Plant Genet. Resour. 14 (4), 256–269. 10.1017/s1479262116000162

[B334] Tripathi S. Sangwan R. S. Narnoliya L. K. Srivastava Y. Mishra B. Sangwan N. S. (2017). Transcription factor repertoire in ashwagandha (withania somnifera) through analytics of transcriptomic resources: Insights into regulation of development and withanolide metabolism. Sci. Rep. 7, 16649. 10.1038/s41598-017-14657-6 29192149 PMC5709440

[B335] Tripathi S. Sangwan R. S. Mishra B. Jadaun J. S. Sangwan N. S. (2020a). Berry transcriptome: Insights into a novel resource to understand development-dependent secondary metabolism in *Withania somnifera* (ashwagandha). Physiol. Plant 168, 148–173. 10.1111/ppl.12943 30767228

[B336] Tripathi S. Srivastava Y. Sangwan R. S. Sangwan N. S. (2020b). In silico mining and functional analysis of AP2/ERF gene in withania somnifera. Sci. Rep. 10, 4877. 10.1038/s41598-020-60090-7 32184405 PMC7078187

[B337] Tripathi G. Dutta S. Mishra A. Basu S. Gupta V. Kamaraj C. (2025). Nanomaterials impact in phytohormone signaling networks of plants− a critical review. Plant Sci. 352, 112373. 10.1016/j.plantsci.2024.112373 39725164

[B338] Trovato M. Forlani G. Signorelli S. Funck D. (2019). Proline metabolism and its functions in development and stress tolerance. Springer International Publishing, 41–72.

[B339] Tyagi P. Singh D. Mathur S. Singh A. Ranjan R. (2022). Upcoming progress of transcriptomics studies on plants: An overview. Front. Plant Sci. 13, 1030890. 10.3389/fpls.2022.1030890 36589087 PMC9798009

[B340] Ullah S. Khan M. N. Lodhi S. S. Ahmed I. Tayyab M. Mehmood T. (2022). Targeted metabolomics reveals fatty acid abundance adjustments in drought-stress response and post-drought recovery in wheat. Front. Genet. 13, 972696. 10.3389/fgene.2022.972696 36437965 PMC9691424

[B341] Upadhyay S. Srivastava Y. (2019). Retrograde response by reactive oxygen/nitrogen species in plants involving different cellular organelles. Biol. Chem. 400 (8), 979–989. 10.1515/hsz-2018-0463 31004559

[B343] Vaughan M. M. Block A. Christensen S. A. Allen L. H. Schmelz E. A. (2018). The effects of climate change associated abiotic stresses on maize phytochemical defenses. Phytochem. Rev. 17, 37–49. 10.1007/s11101-017-9508-2

[B344] Velikova V. Várkonyi Z. Szabó M. Maslenkova L. Nogues I. Kovács L. (2011). Increased thermostability of thylakoid membranes in isoprene-emitting leaves probed with three biophysical techniques. Plant Physiol. 157, 905–916. 10.1104/pp.111.182519 21807886 PMC3192565

[B347] Vrahatis A. G. Lazaros K. Kotsiantis S. (2024). Graph attention networks: a comprehensive review of methods and applications. Future Internet 16, 318. 10.3390/fi16090318

[B348] Walsh J. J. Mangina E. Negrão S. (2024). Advancements in imaging sensors and AI for plant stress detection: A systematic literature review. Plant Phenomics 6, 0153. 10.34133/plantphenomics.0153 38435466 PMC10905704

[B350] Wang Y. Zhang X. Huang G. Feng F. Liu X. Guo R. (2020). Dynamic changes in membrane lipid composition of leaves of winter wheat seedlings in response to PEG-Induced water stress. BMC Plant Biol. 20, 2257. 10.1186/s12870-020-2257-1 32085729 PMC7035713

[B351] Wang Y. Li J. Chen Y. Yu Z. Liu P. Li G. (2021). Genome-wide identification of TCP transcription factors and their potential roles in hydrolyzable tannin production in Quercus variabilis cupule. Front. Plant Sci. 15, 1444081. 10.3389/fpls.2024.1444081 PMC11333348 39166255

[B352] Wang D. Song F. Zhou Y. Zhong T. Zhang Y. Deng Q. (2024). Effects of alkaline salt stress on growth, physiological properties and medicinal components of clonal glechoma longituba (nakai) kupr. BMC Plant Biol. 24, 965. 10.1186/s12870-024-05668-3 39402458 PMC11475845

[B353] Wang R. Hastings W. J. Saliba J. G. Bao D. Huang Y. Maity S. (2024). Applications of nanotechnology for spatial omics: biological structures and functions at nanoscale resolution. ACS Nano 19, 73–100. 10.1021/acsnano.4c11505 39704725 PMC11752498

[B354] Wang Y. Chen J. He G. Yin L. Liao Y. (2025a). Unlocking the potential of flavonoid biosynthesis through integrated metabolic engineering. Front. Plant Sci. 16, 1597007. 10.3389/fpls.2025.1597007 40510168 PMC12158926

[B355] Wang Y. Zhou W. Wang Z. Gao S. Zhang R. (2025b). Integrated metabolome, transcriptome and physiological analyses of melatonin-induced drought responses in maize roots and leaves. Plant Growth Regul. 105, 229–244. 10.1007/s10725-024-01272-5

[B349] Wang C. Hao N. Li Y. Sun N. Wang L. Ye Y. (2025c). Cold-tolerance candidate gene identification in maize germination using BSA. Transcr. Metabolome Profiling. Agron. 15, 1067. 10.3390/agronomy15051067

[B356] Wang H. Zhang Y. Norris A. Jiang C. Z. (2022). S1-bzip transcription factors play important roles in the regulation of fruit quality and stress response. Front. Plant Sci. 12, 802802. 10.3389/fpls.2021.802802 35095974 PMC8795868

[B357] Wang L. Ning C. Pan T. Cai K. (2022). Role of silica nanoparticles in abiotic and biotic stress tolerance in plants: A review. Int. J. Mol. Sci. 23, 1947. 10.3390/ijms23041947 35216062 PMC8872483

[B358] Wang Y. Liu J. Yang F. Zhou W. Mao S. Lin J. (2024). Untargeted LC–MS-Based metabolomics revealed specific metabolic changes in cotyledons and roots of ricinus communis during early seedling establishment under salt stress. Plant Physiology Biochem. 163, 108–118. 10.1016/j.plaphy.2021.03.019 33826995

[B359] Washburn J. D. Mejia-Guerra M. K. Ramstein G. Kremling K. A. Valluru R. Buckler E. S. (2019). Evolutionarily informed deep learning methods for predicting relative transcript abundance from DNA sequence. Proc. Natl. Acad. Sci. 116, 5542–5549. 10.1073/pnas.1814551116 30842277 PMC6431157

[B360] Wei J. Wang A. Li R. Qu H. Jia Z. (2018). Metabolome-wide association studies for agronomic traits of rice. Heredity 120, 342–355. 10.1038/s41437-017-0032-3 29225351 PMC5842221

[B361] Wei Z. Zhongbing C. Xiuqin Y. Luying S. Huan M. Sixi Z. (2023). Integrated transcriptomics and metabolomics reveal key metabolic pathway responses in pistia stratiotes under cd stress. J. Hazard. Mater. 452, 131214. 10.1016/j.jhazmat.2023.131214 36989786

[B362] Westhues C. C. Mahone G. S. Silva S. Thorwarth P. Schmidt M. Richter J. C. (2021). Prediction of maize phenotypic traits with genomic and environmental predictors using gradient boosting frameworks. Front. Plant Science 12, 699589. 10.3389/fpls.2021.699589 PMC8647909 34880880

[B364] Wu Y. Xie L. (2025). AI-driven multi-omics integration for multi-scale predictive modeling of genotype-environment-phenotype relationships. Comput. Struct. Biotechnol. J. 27 (2025), 265–277. 10.1016/j.csbj.2024.12.030 39886532 PMC11779603

[B365] Wu C. Wang Y. Sun H. (2023). Targeted and untargeted metabolomics reveals deep analysis of drought stress responses in needles and roots of Pinus taeda seedlings. Front. Plant Sci. 13, 1031466. 10.3389/fpls.2022.1031466 36798806 PMC9927248

[B367] Wu W. Wu H. Liang R. Huang S. Meng L. Zhang M. (2025). Light regulates the synthesis and accumulation of plant secondary metabolites. Front. Plant Sci. 16, 1644472. 10.3389/fpls.2025.1644472 40831721 PMC12358498

[B368] Wurtzel E. T. Kutchan T. M. (2016). Plant metabolism, the diverse chemistry set of the future. Science 353, 1232–1236. 10.1126/science.aad2062 27634523

[B369] Xavier V. Spréa R. Finimundy T. C. Heleno S. A. Amaral J. S. Barros L. (2023). Terpenes. Springer International Publishing, 107–156.

[B370] Xiang N. Li C. Li G. Yu Y. Hu J. Guo X. (2019). Comparative evaluation on vitamin e and carotenoid accumulation in sweet corn (*Zea mays* l.) seedlings under temperature stress. J. Agric. Food Chem. 67, 9772–9781. 10.1021/acs.jafc.9b04452 31398019

[B371] Xiao J. Gu C. He S. Zhu D. Huang Y. Zhou Q. (2021). Widely targeted metabolomics analysis reveals new biomarkers and mechanistic insights on chestnut (*Castanea mollissima*) calcification process. Food Res. Int. 141, 110128. 10.1016/j.foodres.2021.110128 33641995

[B372] Xiong H. He H. Chang Y. Miao B. Liu Z. Wang Q. (2025). Multiple roles of NAC transcription factors in plant development and stress responses. J. Integr. Plant Biol. 67, 510–538. 10.1111/jipb.13854 39950532

[B373] Xu J. Chen Z. Wang F. Jia W. Xu Z. (2020). Combined transcriptomic and metabolomic analyses uncover rearranged gene expression and metabolite metabolism in tobacco during cold acclimation. Sci. Rep. 10, 5242. 10.1038/s41598-020-62111-x 32251321 PMC7090041

[B374] Xu P. Wu L. Cao M. Ma C. Xiao K. Li Y. (2021). Identification of MBW complex components implicated in the biosynthesis of flavonoids in woodland strawberry. Front. Plant Sci. 12, 774943. 10.3389/fpls.2021.774943 34819941 PMC8606683

[B376] Xu S. Bai C. Chen Y. Yu L. Wu W. Hu K. (2024). Comparing univariate filtration preceding and succeeding PLS-DA for untargeted LC–MS metabolomics. Anal. Chim. Acta 1287, 342103. 10.1016/j.aca.2023.342103 38182346

[B377] Yadav R. K. Sangwan R. S. Sabir F. Srivastava A. K. Sangwan N. S. (2014). Effect of prolonged water stress on specialized secondary metabolites, peltate glandular trichomes, and pathway gene expression in artemisia annua l. Plant Physiol. &Biochemistry 74, 70–83. 10.1016/j.plaphy.2013.10.023 24269871

[B378] Yadav R. K. Sangwan R. S. Srivastava A. K. Sangwan N. S. (2017). Prolonged exposure to salt stress affects specialized metabolites-artemisinin and essential oil accumulation in Artemisia annua L.: metabolic acclimation in preferential favour of enhanced terpenoid accumulation accompanying vegetative to reproductive phase transition. Protoplasma 254 (1), 505–522. 10.1007/s00709-016-0971-1 27263081

[B379] Yaginuma S. Kawana H. Aoki J. (2022). Current knowledge on mammalian phospholipase A1, brief history, structures, biochemical and pathophysiological roles. Molecules 27 (8), 2487. 10.3390/molecules27082487 35458682 PMC9031518

[B380] Yamada Y. Sato F. (2021). Transcription factors in alkaloid engineering. Biomolecules 11, 1719. 10.3390/biom11111719 34827717 PMC8615522

[B381] Yang C. Hu Z. Lu M. Li P. Tan J. Chen M. (2018). Application of metabolomics profiling in the analysis of metabolites and taste quality in different subtypes of white tea. Food Res. Int. 106, 909–919. 10.1016/j.foodres.2018.01.069 29580004

[B382] Yang X. Lu M. Wang Y. Wang Y. Liu Z. Chen S. (2021). Response mechanism of plants to drought stress. Horticulturae 7 (3), 50. 10.3390/horticulturae7030050

[B383] Yang J. Qu X. Ji L. Li G. Wang C. Wang C. (2022). PIF4 promotes expression of HSFA2 to enhance basal thermotolerance in arabidopsis. Int. J. Mol. Sci. 23, 6017. 10.3390/ijms23116017 35682701 PMC9181434

[B384] Yang Y. Saand M. A. Huang L. Abdelaal W. B. Zhang J. Wu Y. (2024). Applications of multi-omics technologies for crop improvement. Front. Plant Sci. 12, 563953. 10.3389/fpls.2021.563953 PMC8446515 34539683

[B387] Yeshi K. Crayn D. Ritmejerytė E. Wangchuk P. (2022). Plant secondary metabolites produced in response to abiotic stresses has potential application in pharmaceutical product development. Molecules 27 (1), 313. 10.3390/molecules27010313 35011546 PMC8746929

[B385] Yang Y. Tilman D. Jin Z. Smith P. Barrett C. B. Zhu Y.-G. (2024b). Climate change exacerbates the environmental impacts of agriculture. Science 385, 3747. 10.1126/science.adn3747 39236181

[B386] Yang X. Feng K. Wang G. Zhang S. Zhao J. Yuan X. (2024). Titanium dioxide nanoparticles alleviate polystyrene nanoplastics-induced growth inhibition by modulating carbon and nitrogen metabolism via melatonin signaling in maize. J. Nanobiotechnology 22, 262. 10.1186/s12951-024-02537-x 38760823 PMC11100085

[B388] Yuan J. Liu R. Sheng S. Fu H. Wang X. (2022). Untargeted LC–MS/MS-Based metabolomic profiling for salvia miltiorrhiza under cadmium stress. Front. Plant Sci. 13, 889370. 10.3389/fpls.2022.889370 35968141 PMC9366474

[B389] Zainal-Abidin R. A. Khalid K. I S. Balfagón D. Gómez-Cadenas A. Mittler R. (2024). “Nanotechnologies and omics: a way forward,” in Innovative methods in horticultural crop improvement. Al-Khayri J Alnaddaf L. M. Jain S. M. Penna S. (Cham, Switzerland: Springer). 1. 10.1007/978-3-031-61081-3_1

[B390] Zandalinas S. I. Balfagón D. Gómez-Cadenas A. Mittler R. (2022). Plant Responses to Climate Change: Metabolic Changes under Combined Abiotic Stresses. J. Exp. Bot. 73 (11), 3339–3354. 10.1093/jxb/erac073 35192700

[B391] Zenda T. Liu S. Dong A. Li J. Duan H. Liu X. (2021). Omics-facilitated crop improvement for climate resilience and superior nutritive value. Front. Plant Sci. 12, 774994. 10.3389/fpls.2021.774994 34925418 PMC8672198

[B392] Zeng J. Zhang Y. Zhang H. Song W. Wu Z. Wang X. (2021). Design and characterization of a semi-open dynamic chamber for measuring biogenic volatile organic compounds (BVOCs) emissions from plants. Atmos. Meas. Tech. Discuss. 2021, 1–37. 10.5194/amt-15-79-2022

[B393] Zhang C. Shi S. (2018). Physiological and proteomic responses of contrasting alfalfa varieties to PEG-Induced osmotic stress. Front. Plant Sci. 9, 242. 10.3389/fpls.2018.00242 29541085 PMC5835757

[B394] Zhang B. Chopra D. Schrader A. Hülskamp M. (2019). Evolutionary comparison of competitive protein-complex formation of MYB, bHLH, and WDR proteins in plants. J. Exp. Bot. 70 (12), 3197–3209. 10.1093/jxb/erz155 31071215 PMC6598095

[B417] Zhang H. Zhao Y. Zhu J. K. (2020). Thriving under stress: how plants balance growth and the stress response. Develop. Cell. 55 (5), 529–543. 10.1016/j.devcel.2020.10.012 33290694

[B396] Zhang Q. Li B. Chen Q. Su Y. Wang R. Liu Z. (2022a). Non-targeted metabolomic analysis of the variations in the metabolites of two genotypes of *Glycyrrhiza uralensis* fisch. under drought stress. Industrial Crops Prod. 176, 114402. 10.1016/j.indcrop.2021.114402

[B397] Zhang Q. Li C. Jiao Z. Ruan J. Liu M. Y. (2022b). Integration of metabolomics and transcriptomics reveal the mechanism underlying accumulation of flavonols in albino tea leaves. Molecules 27, 5792. 10.3390/molecules27185792 36144526 PMC9501457

[B400] Zhang D. Liu Y. Yang Z. Song X. Ma Y. Zhao J. (2023). Widely targeted metabolomics analysis of metabolite differences of licorice under drought stress. Industrial Crops Prod. 202, 117071. 10.1016/j.indcrop.2023.117071

[B401] Zhang X. Han C. Wang Y. Liu T. Liang Y. Cao Y. (2024a). Integrated analysis of transcriptomics and metabolomics of garden asparagus (*Asparagus officinalis* l.) under drought stress. BMC Plant Biol. 24, 563. 10.1186/s12870-024-05286-z 38879466 PMC11179350

[B402] Zhang X. Yang H. Du T. (2024b). Coupled mechanisms of water deficit and soil salinity affecting tomato fruit growth. Agric. Water Manag. 295, 108747. 10.1016/j.agwat.2024.108747

[B403] Zhang B. Lam T. K. Y. Chen L. Zhang C. Zhu L. Zhang H. (2025). Single-cell transcriptomics and time-series metabolite profiling reveal the spatiotemporal regulation of flavonoid biosynthesis genes and phytohormone homeostasis by PAP1 in Arabidopsis. BMC Biology 23, 191. 10.1186/s12915-025-02297-6 40598113 PMC12220432

[B404] Zhang L. Li Y. Zhang L. Liu Q. Zheng S. Lou H. (2025). Terpene synthase TgHPTPS2 from torreya grandis modulates terpenoid profiles to balance ROS and confer drought tolerance in plants. Int. J. Biol. Macromol. 314, 144402. 10.1016/j.ijbiomac.2025.144402 40398781

[B405] Zhang S. Chen J. Ma Y. Zhao Q. Jing B. Yu M. (2025). Green synthesis, biomedical effects, and future trends of Ag/ZnO bimetallic nanoparticles: An update. Nanotechnol. Rev. 14 (1), 20250186. 10.1515/ntrev-2025-0186

[B407] Zhao Y. Min T. Chen M. Wang H. Zhu C. Jin R. (2021). The photomorphogenic transcription factor PpHY5 regulates anthocyanin accumulation in response to UVA and UVB irradiation. Front. Plant Sci. 11, 603178. 10.3389/fpls.2020.603178 33537042 PMC7847898

[B408] Zhao M. Huang J. Ren J. Xiao X. Li Y. Zhai L. (2024). Metabolomic insights into primary and secondary metabolites variation in common and glutinous rice (*Oryza sativa* L.). Agronomy 14, 1383. 10.3390/agronomy14071383

[B409] Zhao Y. Li S. Wu J. Liu H. Xu L. (2024). Insights into membrane lipids modification in barley leaves as an adaptation mechanism to cold stress. Plant Growth Regul. 103, 369–388. 10.1007/s10725-023-01114-w

[B410] Zheljazkov V. D. Astatkie T. Shiwakoti S. Poudyal S. Horgan T. Kovatcheva N. (2014). Essential oil yield and composition of garden sage as a function of different steam distillation times. HortScience 49 (6), 785–790. 10.21273/hortsci.49.6.785

[B411] Zhou X. Li J. Wang Y. Liang X. Zhang M. Lu M. (2022). The classical SOS pathway confers natural variation of salt tolerance in maize. New Phytol. 236, 479–494. 10.1111/nph.18278 35633114

[B412] Zhou Y. Nie K. Geng L. Wang Y. Li L. Cheng H. (2024). Selenium’s role in plant secondary metabolism: regulation and mechanistic insights. Agronomy 15, 54. 10.3390/agronomy15010054

[B413] Zhu X. Zhang M. Wang B. Song X. Wang X. Wei X. (2023). Non-targeted metabolomics analysis of metabolite changes in two quinoa genotypes under drought stress. BMC Plant Biol. 23, 503. 10.1186/s12870-023-04467-6 37858063 PMC10588040

[B414] Zulfiqar F. Ashraf M. (2023). Proline alleviates abiotic stress induced oxidative stress in plants. J. Plant Growth Regul. 42, 4629–4651. 10.1007/s00344-022-10839-3

[B415] Zuo Z. Wang B. Ying B. Zhou L. Zhang R. (2017). Monoterpene emissions contribute to thermotolerance in cinnamomum camphora. Trees 31, 1759–1771. 10.1007/s00468-017-1582-y

